# Molecular-scale features that govern the effects of *O*-glycosylation on a carbohydrate-binding module†
†This manuscript is dedicated to Professor Samuel J. Danishefsky.
‡
‡Electronic supplementary information (ESI) available. See DOI: 10.1039/c5sc02636a



**DOI:** 10.1039/c5sc02636a

**Published:** 2015-09-21

**Authors:** Xiaoyang Guan, Patrick K. Chaffey, Chen Zeng, Eric R. Greene, Liqun Chen, Matthew R. Drake, Claire Chen, Ari Groobman, Michael G. Resch, Michael E. Himmel, Gregg T. Beckham, Zhongping Tan

**Affiliations:** a Department of Chemistry and Biochemistry , BioFrontiers Institute , University of Colorado , Boulder , CO 80303 , USA . Email: zhongping.tan@colorado.edu; b National Bioenergy Center , National Renewable Energy Laboratory , Golden , CO 80401 , USA . Email: gregg.beckham@nrel.gov; c Biosciences Center , National Renewable Energy Laboratory , Golden , CO 80401 , USA

## Abstract

Protein glycosylation is a ubiquitous post-translational modification in all kingdoms of life. Despite its importance in molecular and cellular biology, the molecular-level ramifications of *O*-glycosylation on biomolecular structure and function remain elusive. Here, we took a small model glycoprotein and changed the glycan structure and size, amino acid residues near the glycosylation site, and glycosidic linkage while monitoring any corresponding changes to physical stability and cellulose binding affinity. The results of this study reveal the collective importance of all the studied features in controlling the most pronounced effects of *O*-glycosylation in this system. Going forward, this study suggests the possibility of designing proteins with multiple improved properties by simultaneously varying the structures of *O*-glycans and amino acids local to the glycosylation site.

## Abstract 2

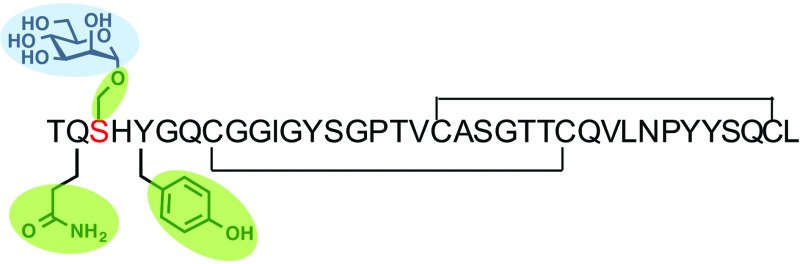
The importance of the glycan structure and size, amino acid residues near the glycosylation site, and glycosidic linkage in controlling the effects of CBM *O*-glycosylation is shown.

## 

The capability of glycans to affect protein properties opens the possibility of custom-designed glycan motifs that can be introduced to produce proteins with desirable properties.^1,2^ Regrettably, due to the current lack of quantitative knowledge about the effects of protein glycosylation, such glycoengineering approaches are still largely empirical, which makes research in this area challenging, time-consuming, and costly.^3^ A detailed, molecular-level understanding of the features and factors associated with the effects of natural glycosylation of proteins would facilitate the process. Recent studies of protein *N*-glycosylation have clearly demonstrated that such information is useful in guiding the glycoengineering of proteins.^4–7^ Unfortunately, unlike *N*-glycosylation, no universal consensus sequence has been identified for *O*-glycosylation, which seriously limits access to glyco-variants and hampers the detailed study and application of *O*-glycosylation.^8–11^

In the present study, we have chosen to investigate the molecular features that control the effects of *O*-glycosylation at a specific site, Ser3, in the Family 1 carbohydrate-binding module (CBM) of the glycoside hydrolase Family 7 cellobiohydrolase from the cellulolytic fungus, *Trichoderma reesei* (*Tr*Cel7A), a key enzyme in the cellulosic biofuels industry (Fig. 1). Family 1 CBMs are small, natively glycosylated, synthetically tractable, and their glycosylation poses interesting stability and functional questions, making them excellent model systems to study *O*-glycosylation.^14,15^ The amino acid Ser3 was chosen for in depth study after we established that, for the CBM, glycosylation at this position is responsible for the most significant enhancements in desirable enzyme properties: proteolytic stability against thermolysin degradation, thermostability, and binding affinity towards bacterial microcrystalline cellulose (BMCC).^16^ The fact that glycosylation at this site caused the largest, and hence most detectable, changes makes glycosylation at Ser3 an ideal choice for identifying the molecular determinants of natural *O*-glycosylation's observed effects in this system.

**Fig. 1:**
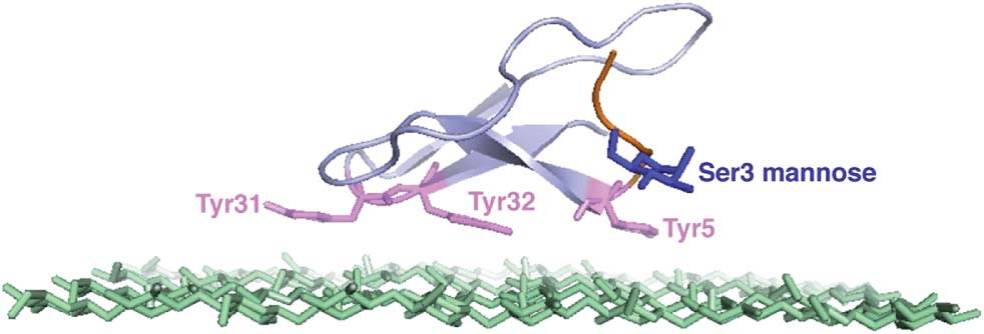
The NMR structure of the Family 1 CBM and the top layer of cellulose.^12^ The tyrosine residues are shown in purple. The *O*-linked mannose at Ser3 site is shown in blue.^13^

We conducted several comparative studies to determine the contributions of multiple molecular features (Fig. 2).^16,17^ Like our previous studies, we first designed and prepared 31 new CBM isoforms with systematic variations in amino acid sequence, glycopeptide linkage, glycan structure, and anomeric configuration to assess the importance of each of these structural elements in mediating the effects of *O*-glycosylation (Fig. 2, **4–34**).^18–20^ Three previously characterized CBM isoforms, which all have the natural amino acid sequence and either no glycans (**1**), a single mannose (**2**), or a single di-mannose (**3**), were also included as controls.^16^

**Fig. 2:**
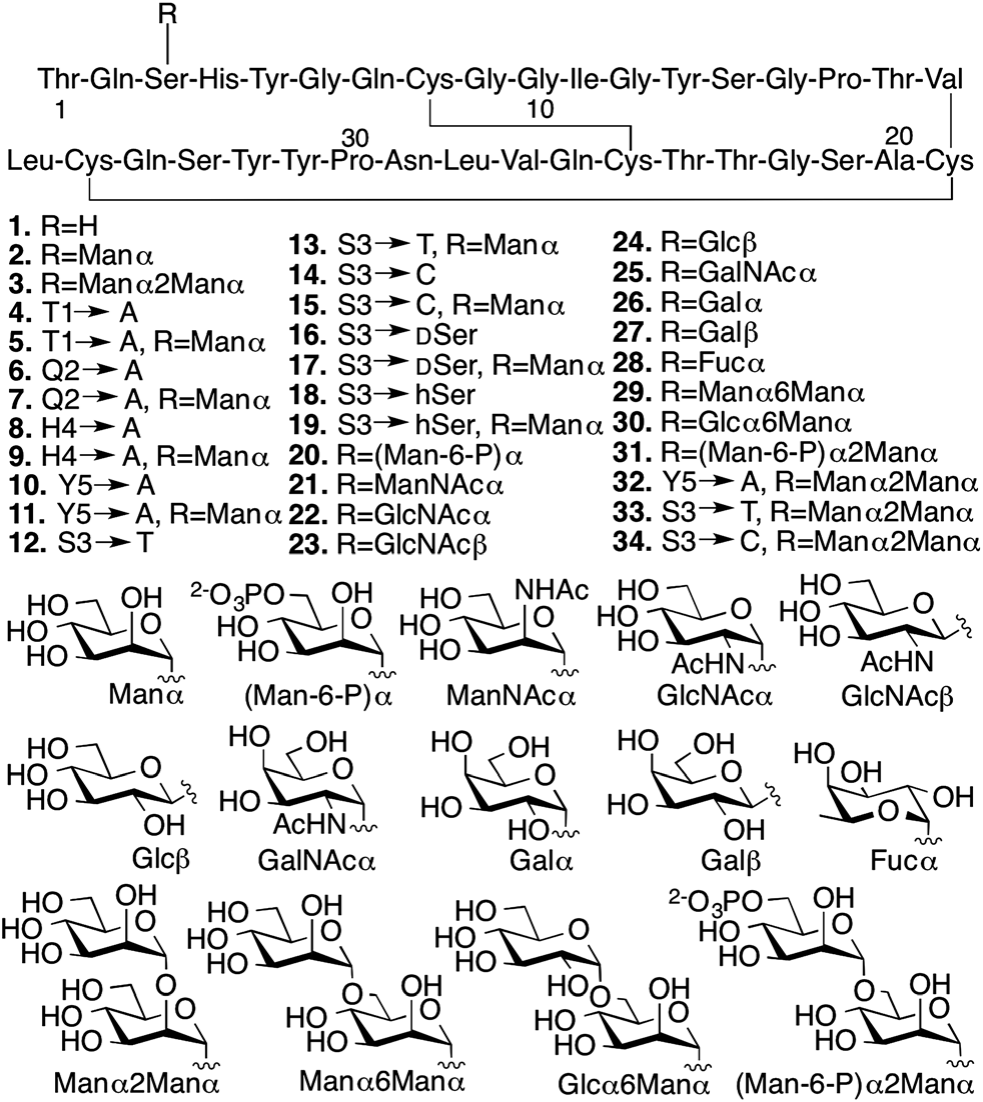
Synthetic CBM isoforms and the structures of *O*-linked glycans.

Since chemical glycosylation is not controlled by the structural features of peptides, it is capable of generating almost any glyco-variant.^8,9,11^ Synthesis of CBM isoforms was conducted with Fmoc-based solid-phase peptide synthesis (SPPS). During SPPS, all sugar hydroxyl and/or phosphate groups on the side chains of the glycoamino acid building blocks where protected as acetyl^16^ or benzyl esters,^21^ respectively, which are stable during peptide coupling procedures and easily removed under carbohydrate-compatible conditions. Since most of the glycoamino acid building blocks used in this study are not commercially available, we first identified efficient synthetic methods to quickly prepare glycosylated Fmoc-Ser, Fmoc-Thr, Fmoc-d-serine (DSer), and homoserine (hSer) in gram scales (ESI, Section II‡). To ensure strict control over anomeric stereochemistry, reaction conditions were carefully chosen for high diastereomeric selectivity and every synthetic glycoamino acid building block was analyzed using 2D HSQC NMR to confirm absolute anomeric configuration. After synthesizing all the desired building blocks, our previously developed one-pot synthesis and folding method enabled us to quickly generate all 31 desired CBM isoforms in high purity and with good yields for glycopeptide synthesis (ranging from 30% for **6** to 6% for **20**) (ESI, Section III‡).^16,22^

With the CBM isoform library completed, we began by investigating how amino acid side chains close to the glycosylation site alter the effects of *O*-glycosylation using Ala-scanning mutagenesis; four mutations were used for this. For each mutation, the unglycosylated CBM was compared to the corresponding mono-mannosylated glycopeptide in terms of proteolytic stability, thermostability, and binding affinity, following previously described protocols (Fig. 3).^6,16,23^ As shown in the left side of Fig. 3 (top panel), Ala mutations at any residue adjacent to the Ser3 glycosylation site (Thr1, Gln2, His4, or Tyr5) did not significantly alter the thermolysin half-life of the unglycosylated CBMs (compare **1**, **4**, **6**, **8** and **10**). Our previous study established that glycosylation of Ser3 significantly stabilized the CBM towards protease degradation,^16^ but this trend holds true in only two of the four Ala-mutant sequences (T1A, compare **4** and **5** and H4A, compare **8** and **9**). In contrast, the attachment of a single mannose to Ser3 in both the Q2A mutant (compare **6** and **7**) and the Y5A mutant (compare **10** and **11**) leads to almost no increase in their thermolysin half-life. Thermostability of these CBM sequences follows a similar trend (Fig. 3, middle panel). The binding affinity exhibits a very different pattern (Fig. 3, bottom panel). For unglycosylated isoforms, replacing Thr1, Gln2, His4, or Tyr5 with Ala induces pronounced and widely variable changes in BMCC binding, from large increases (Q2A, **6** and H4A, **8**) to totally eliminating binding (Y5A, **10**).^14^ Mono-mannosylation of any of these mutants gives only small negative or positive deviations to the binding constant.

**Fig. 3:**
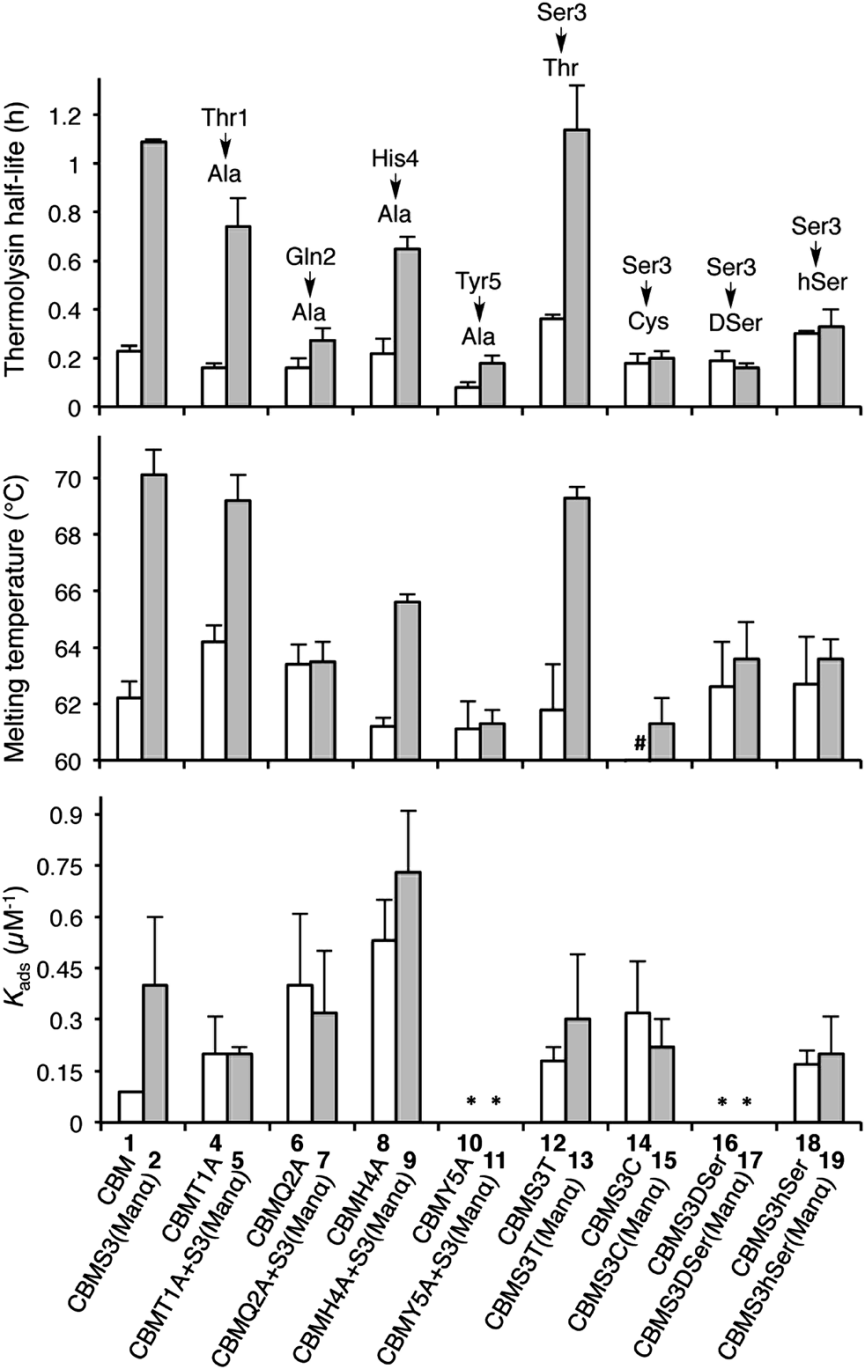
The contributions of amino acids to the effects of the Ser3 glycosylation on the proteolytic stability (half-life to thermolysin degradation), thermostability (melting temperatures measured by variable temperature CD), and binding affinity (*K*_ads_ values on BMCC) of the *Tr*Cel7A CBM. All error bars reported are standard deviations of data achieved from three separate trials. The structural feature of each isoform is implied by its name, *i.e.* CBMS3(Manα) representing the isoform containing a single mannose α-linked to Ser3, CBMQ2A + S3(Manα) representing the isoform containing a Gln-to-Ala mutation at position 2 and a single mannose α-linked to Ser3, and CBMS3hSer(Manα) representing the isoform containing a Ser-to-hSer mutation at position 3 and a single mannose α-linked to hSer3. # 53 °C. * No observable binding noted.

To quantify how side-chain properties like hydrophobicity, glycosidic bond character, side-chain orientation, and length alter the influence of mannosylation, Ser3 was replaced by four similar amino acids: Thr **12**/**13**, Cys **14**/**15**, DSer **16**/**17**, and hSer **18**/**19**. As shown in the right side of Fig. 3 (top and middle panel), replacement of Ser3 by Thr has little influence on the stability of either unglycosylated or mannosylated CBM. Replacement by Cys, DSer, or hSer, however, significantly diminishes the stabilizing effect of mannose. Thermostability followed a comparable trend. Interestingly, CBM variant **14** has a 10 °C lower melting temperature than that of CBM **1**. This may be a result of less stable disulfide bonds in the presence of a free Cys.^24^ Capping the free sulfhydryl group with a mannose brings the melting temperature back up to 61 °C. Binding affinity of the unglycosylated CBM increased upon substitution of Ser3 by Thr, Cys, or hSer (compare **1** to **12**, **14**, and **18**), but mannosylation of these mutant CBMs shows a very different trend. Both Thr and hSer-containing isoforms showed insignificant increases in binding affinity upon glycosylation (compare **12** to **13** and **18** to **19**), while glycosylation of the Ser-to-Cys mutation results in a small decrease (compare **14** and **15**). Neither of the DSer mutants (**16** and **17**) shows any obvious binding to BMCC.

Understanding the impact of glycan composition and linkage stereochemistry on the effects of Ser3 glycosylation was our next goal. For this, we directly compared CBM glycoforms with systematically varied glycan structures in two final studies (Fig. 4). To elucidate the potentially variable influence of different mono-saccharides nine CBM glycoforms, **20–28**, were compared to unglycosylated **1** and mannosylated **2**. As shown in Fig. 4A, half-lives towards thermolysin degradation and melting temperatures vary in a remarkably similar pattern across these isoforms, with the mannosylated isoform **2** having the highest of both types of stability. Changes to binding affinity followed a distinctly different pattern, although the three CBM glyco-variants with the lowest stabilities (**23**, **24**, and **27**), also have low affinities to the BMCC substrate. Of particular note, we observe that the anomeric stereochemistry of the glycosidic linkage has a more significant influence than most other structural features on the effects of glycosylation (compare **1** to **2**, **22**, and **23**). While the α-linked mono-saccharides on **2** and **22** gave significant improvements over the unglycosylated **1**, the β-linked mono-saccharide on **23** had almost no effect on the proteolytic stability, thermostability, or binding affinity of the CBM. Similarly, the α-linked galactose on **26** significantly improved the melting temperature and modestly improved the proteolytic stability, but the same galactose attached through a β-linkage in **27** gave almost no increase in either property. To probe the influence of a second glycan unit, we also examined six new CBM glyco-variants containing either α1,2-(**3**, **31**, **32**, **33**, and **34**) or α1,6-(**29** and **30**) glycosidic linkages. Once again, as shown in Fig. 4B, the proteolytic stability and thermostability exhibit similar trends after attachment of the additional sugar residues while the binding affinity varies independently. Only the attachment of α1,2-linked mono-mannose to Man-α-Ser (**3**) and Man-α-Thr (**33**) causes a further increase over mono-mannosylated CBM (**2**) in either stability measure. Similarly, only the attachment of α1,6-linked mono-glucose to Man-α-Ser (**30**) and α1,2-linked mono-mannose to Man-α-Thr (**33**) causes a further increase in the binding affinity. Mutating Tyr5 to Ala or Ser3 to Cys significantly diminishes or even abolishes the effects of glycosylation (compare **3** to **32** and **34**). Phosphorylation of the 6-hydroxyl of both mono- and di-mannose, which may naturally occur in Family 1 CBMs, adversely impacts the effects of mannosylation (compare **2** to **20** and **3** to **31**).^25^

**Fig. 4:**
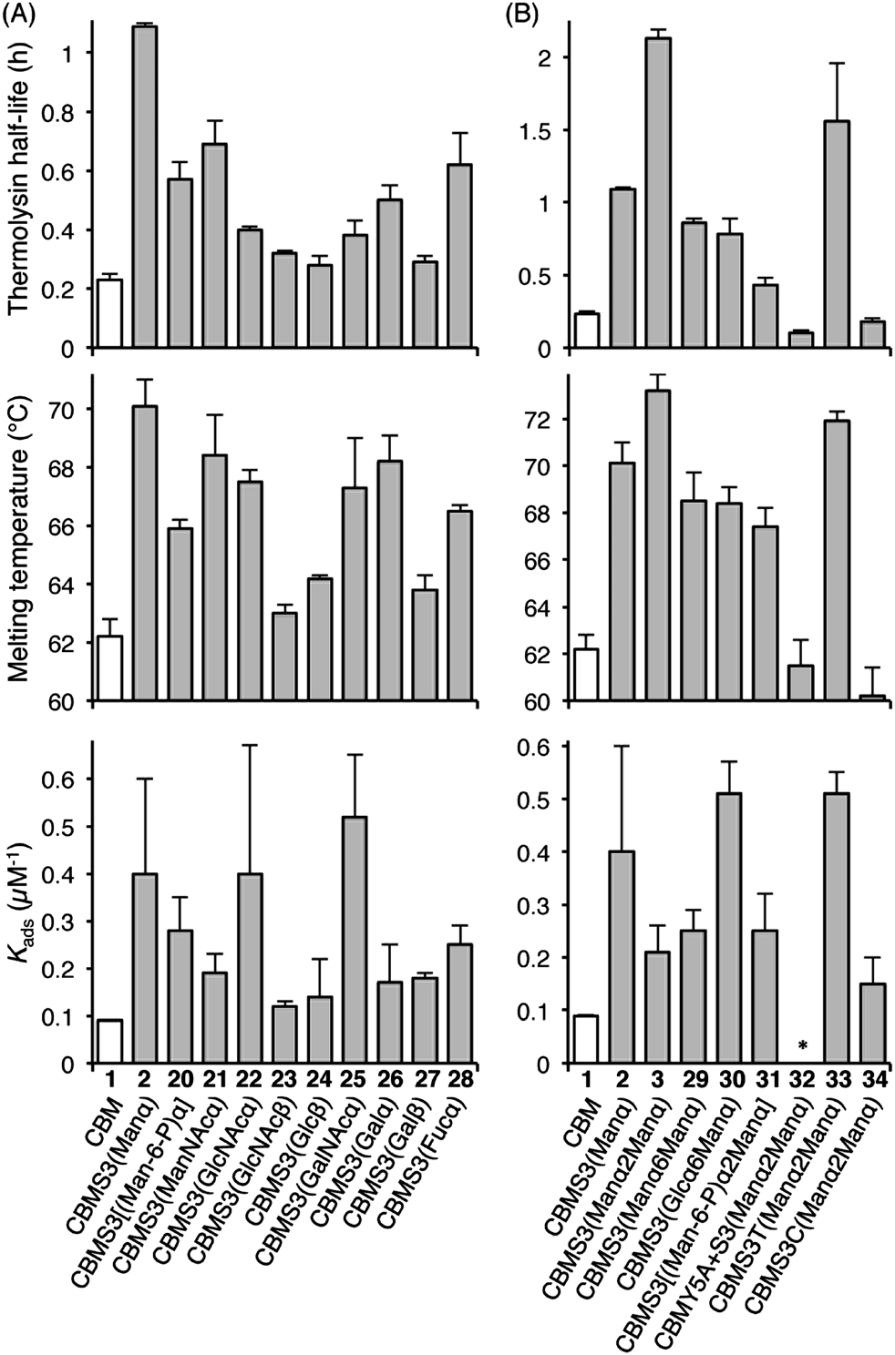
The contributions of different glycans to the effects of Ser3 glycosylation on the proteolytic stability, thermostability, and binding affinity of the *Tr*Cel7A CBM. All error bars reported are standard deviations of data achieved from three separate trials. * No observable binding noted.

The results obtained by comparing the properties of 34 CBM isoforms provide new insights into the molecular determinants of the effects of *O*-glycosylation on the stability and function of this protein. A well-established effect of protein glycosylation is an increase in proteolytic stability, either by increasing the rigidity of the protein, or by providing a steric barrier that hinders protease access to the peptide bonds.^19,26–28^ Our results indicate that steric hindrance may be less important than peptide rigidity in the case of CBM *O*-glycosylation. Support for this conclusion comes from the CBM variants **4–19** (Fig. 3). Since the sizes of their glycan moieties are identical, the differences observed in their susceptibilities to thermolysin hydrolysis can be attributed to altered conformational rigidity.^29^ More specifically, the rigidity seems largely controlled by Gln2, Tyr5 and the glycosylated amino acid residue because glyco-variants with Gln2-to-Ala, Tyr5-to-Ala, or Ser3-to-Cys, DSer, or hSer mutations do not exhibit large changes to the proteolytic stability upon glycosylation. Further support for the limited role of steric hindrance in thermolysin resistance comes from the results of the analysis of CBM variants **20–34**. As shown in Fig. 4, different extents of proteolytic stability are conferred by different mono- or di-saccharides of similar sizes at Ser3 and the stereochemistry at the anomeric carbon plays a large role in modulating the proteolytic stability.

Thermostability is another important property known to be affected by glycosylation.^2^ Recent studies have suggested that local interactions, such as carbohydrate–aromatic interactions, strongly contribute to the large stabilizing impact of *N*-glycosylation.^2,6,30^ Other studies into *O*-glycosylation have also revealed the importance of local interactions between carbohydrate and peptide for *O*-glycopeptide conformation.^31^ Our results here continue to support this conclusion for *O*-glycosylation. Mutating Tyr5 to Ala (compare **11**, **32** and **2**) led to a substantial decrease in the thermostability. In addition, we observe a loss of mannosylation-induced stability for the Q2A mutant. The specific role played by Gln2 is not clear, but previous findings from studies of protein–carbohydrate interactions suggest that its planar polar side chain may be involved in several hydrogen bonds linking the protein and glycan.^32,33^ The importance of these local interactions in stabilizing the CBM is further underscored by the fact that the β-linked glycans have very limited effects on CBM thermostability. This can be explained by decreased contact between glycan and nearby amino acids since the β-glycosidic linkages directs the glycan away from the peptide.^34^

One important question in glycobiology is whether altered biophysical properties and biological function of glycoproteins are related.^2,35^ The answer to this question is critical to the practice of glycoengineering. A positive answer would imply that it is possible to simultaneously increase protein stability and function by glycosylation. As shown in Fig. 5A, our results reveal a striking correlation between variations in the CBM's proteolytic stability and thermal stability, suggesting common molecular forces are responsible for both the thermostabilizing effects of mannosylation and increasing the rigidity of the same site.^31^ Most interestingly, our study reveals a strong link between glycoprotein stability and function: CBM glyco-variants with much lower affinities towards BMCC generally also have low stabilities, those with higher binding affinities often have intermediate stabilities, and the highest stabilities do not necessarily correlate with the highest binding affinities (Fig. 5B). Existing theories shed some light on these observations: intermediate stability or flexibility would allow the CBM to maintain its native structure in solution while permitting the peptide to adopt optimal conformations for dynamically binding to cellulose.^36^

**Fig. 5:**
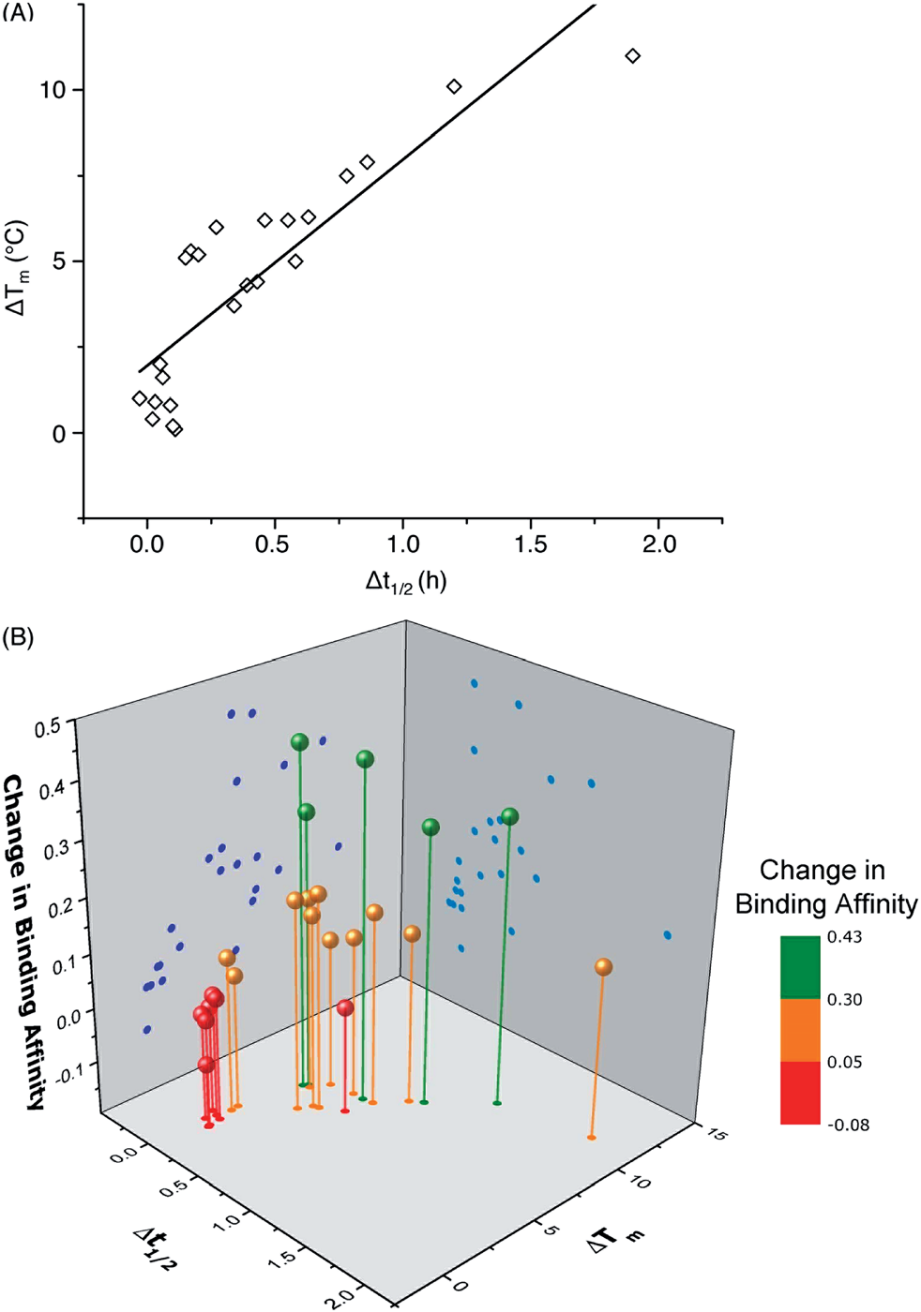
Correlation of (A) the change in melting temperature (Δ*T*_m_) and change in half-life during thermolysin degradation (Δ*t*_1/2_), and (B) the change in binding affinity upon glycosylation. Data points represent differences between CBM glyco-variants and their corresponding unglycosylated counterparts. The data for the CBM pairs **15**/**14** and **34**/**14** are not included in the plot because of their unique characteristics.

In summary, by using chemical synthesis, we were able to systematically vary the amino acid sequence at the *N*-terminal end of a model Family 1 CBM and the glycan structures at Ser3, a highly conserved and functionally important glycosylation site.^17^ By comparing these variants' characteristics, this study provides new insights into the molecular basis for the effects of CBM Ser3 *O*-glycosylation. We have shown that planar polar (Gln) and aromatic amino acid (Tyr) residues as well as *O*-glycans α-linked to Ser or Thr are important for the effects of CBM *O*-glycosylation. More importantly, our data suggest that CBM proteolytic and thermostability are linearly related while the CBM function (*i.e.*, binding affinity) peaks at moderate levels of stability. This type of knowledge is expected to facilitate future investigations into the glycosylation of other proteins, including those with therapeutic and industrial relevance. Although there are many challenges remaining, this work is one small but significant contribution to the currently opaque process of rationally engineering proteins, and provides an illustrative example of simultaneously improving stability and function.
