# Supplementary material for: Molecular-scale features that govern the effects of O-glycosylation on a carbohydrate-binding module
Source: Chem Sci. 2015 Sep 21;6(12):7185–9. doi: 10.1039/c5sc02636a (PMC5580309; doi:10.1039/c5sc02636a)
Supplement: Supplementary file 1 [file SC-006-C5SC02636A-s001.pdf]

Electronic Supplementary Material (ESI) for Chemical Science.
This journal is © The Royal Society of Chemistry 2015

Supporting Information

Molecular-Scale Features that Govern the Effects of O-Glycosylation on a Carbohydrate-Binding Module

Xiaoyang Guan[1,‡], Patrick K. Chaffey[1, ‡], Chen Zeng[1, ‡], Eric R. Greene[1], Liqun Chen[1], Matthew R. Drake[1], Claire Chen[1], Ari Groobman[1], Michael G. Resch[2], Michael E. Himmel[2], Gregg T. Beckham[3,*], Zhongping Tan[1,*]

[1] Department of Chemistry and Biochemistry and BioFrontiers Institute, University of Colorado, Boulder CO 80303;
[2] National Bioenergy Center, National Renewable Energy Laboratory, Golden CO 80401;
[3] Biosciences Center, National Renewable Energy Laboratory, Golden CO 80401

‡ Equal contribution

* Corresponding authors

Gregg T. Beckham, National Bioenergy Center, National Renewable Energy Laboratory, Golden, CO 80401 USA, +01 (303) 384-7806, gregg.beckham@nrel.gov

Zhongping Tan, Department of Chemistry and Biochemistry and BioFrontiers Institute, University of Colorado, Boulder, CO 80303 USA, +01 (303) 492-5160, zhongping.tan@colorado.edu

I. MATERIALS AND METHODS

All commercial reagents and solvents were used as received. Unless otherwise noted, all reactions and purifications were performed under air atmosphere at room temperature. All LC-MS analyses were performed using a Waters Acquity™ Ultra Performance LC system equipped with Acquity UPLC® BEH 300 C4, 1.7μm, 2.1 x 100 mm column at flow rates of 0.3 and 0.5 mL/min. The mobile phase for LC-MS analysis was a mixture of H_2O (0.1% formic acid, v/v) and acetonitrile (0.1% formic acid, v/v). All preparative separations were performed using a LabAlliance HPLC solvent delivery system equipped with a Rainin UV-1 detector and a Varian Microsorb 100-5, C18 250x21.4 mm column at a flow rate of 16.0 mL/min. The mobile phase for HPLC purification was a mixture of H_2O (0.05% TFA, v/v) and acetonitrile (0.04% TFA, v/v). A Waters SYNAPT G2-S system was used mass spectrometric analysis. All circular dichroism (CD) spectra were obtained using an Applied PhotophysicsChirascan™-plus CD spectrometer.

II. SYNTHESIS OF GLYCOAMINO ACIDS

The glycoamino acid building blocks Fmoc-Ser(Ac4Manα1)-OH (**35**), Fmoc-Ser(Ac4Manα1-2Ac$_3$Manα1)-OH (**36**), Fmoc-Thr(Ac4Manα1)-OH (**37**), and Fmoc-Thr(Ac4Manα1-2Ac$_3$Manα1)-OH (**53**) were prepared according to the previously reported method [1]. Glycoamino acid building block **44** was purchased from AnaSpec. All the other building blocks, **38**-**43**, **45**-**52**, and **54** are prepared as described below. The spectroscopic characterizations (^{1}H NMR, ^{13}C NMR, IR, and high-resolution MS) of all new compounds are reported.

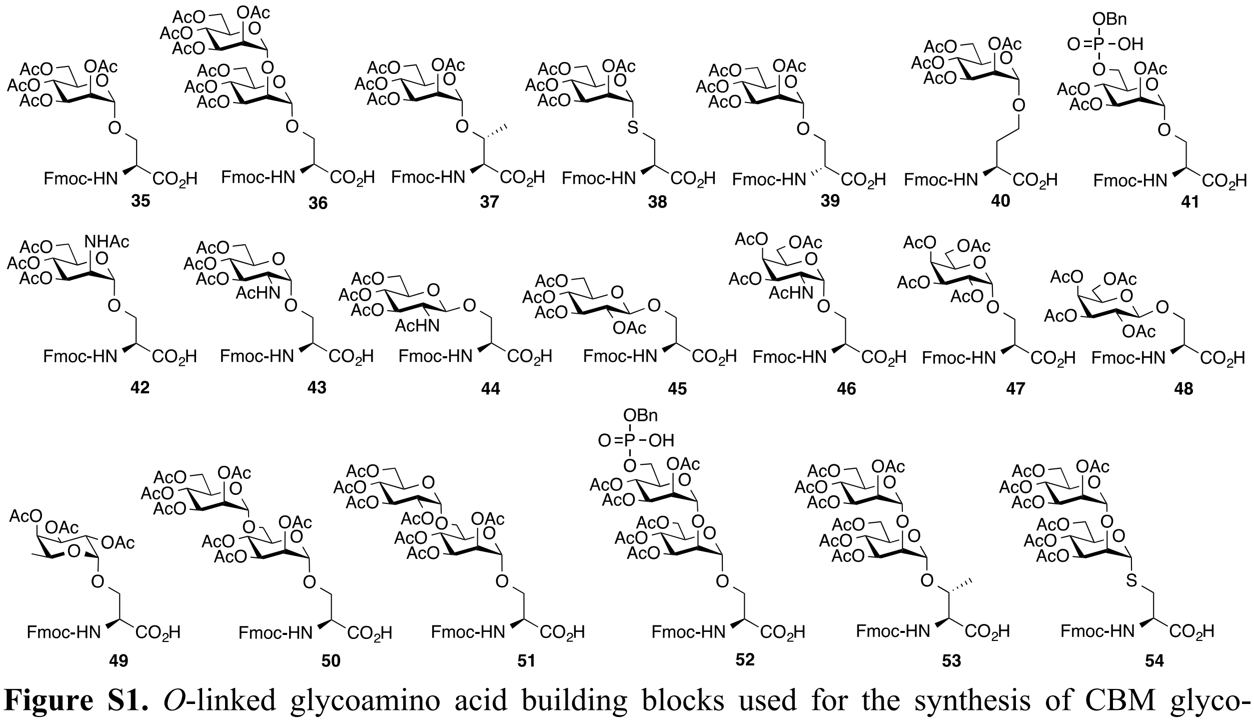

Figure S1. *O*-linked glycoamino acid building blocks used for the synthesis of CBM glyco-variants.

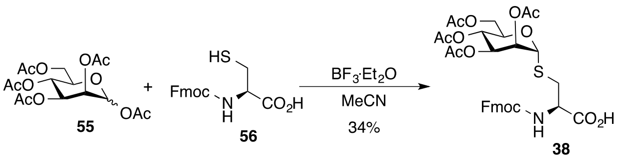

Synthesis of glycoamino acid 38. The 1,2,3,4,6-penta-O-acetyl-D-mannopyranose **55** was prepared as reported in the literature[1]. To the solution of **55** (682 mg, 1.75 mmol) and **56** (Fmoc-Cys-OH, 900 mg, 2.62 mmol) in MeCN (35 ml), $BF_3{\cdot}OEt_2$ (0.81 ml, 5.25 mmol) was added. The resulting mixture was stirred at room temperature for 28 h under argon. The solvent was removed under reduced pressure, and the resulting residue was diluted with EtOAc then washed with water. The organic layer was dried over Na_2SO_4, filtered and the filtrate was concentrated under reduced pressure. The resulting oil was purified by flash chromatography on a silica gel column (Hex/EtOAc/AcOH = 4:1:0.5→3:1:0.4→2:1:0.3) to give **38** (404 mg, 34%) as a white foam.

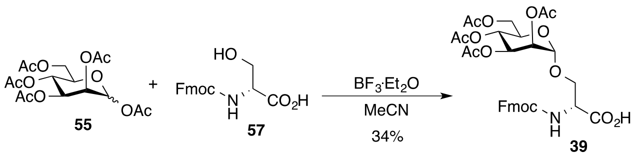

Synthesis of glycoamino acid 39. To the solution of **55** (390 mg, 1.0 mmol) and **57** (Fmoc-DSer-OH, 490 mg, 1.5 mmol) in MeCN (12 ml), $BF_3{\cdot}OEt_2$ (0.38 ml, 3.0 mmol) was added. The resulting mixture was stirred at room temperature for 23 h under argon. The solvent was removed under reduced pressure, and the resulting residue was diluted with EtOAc then washed with water. The organic layer was dried over Na_2SO_4, filtered and the filtrate was concentrated under reduced pressure. The resulting oil was purified by flash chromatography on a silica gel column (Hex/EtOAc/AcOH = 4:1:0.5→3:1:0.4→2:1:0.3) to give **39** (223 mg, 34%) as a white foam.

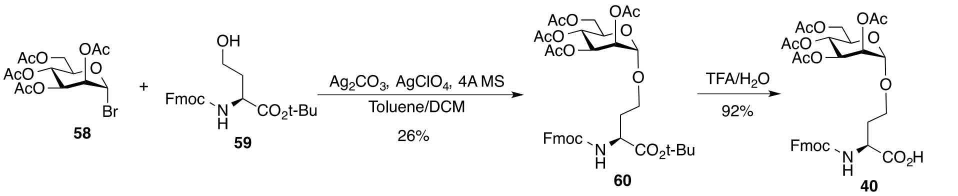

Synthesis of glycoamino acid 40. The 2,3,4,6 tetra-O-acetyl mannosyl bromide **58** was prepared as reported in the literature[1]. A solution of **59** (1.0 g, 2.51 mmol), Ag_2CO_3 (1.03 g, 3.77 mmol), 4A molecular sieves (4A MS) (2.5 g) in toluene (8.5 ml) and DCM (12.8 ml) was stirred at 0 ^oC for 30 minutes[2]. Then, a solution of $AgClO_4$ (130 mg, 0.63 mmol) in toluene (3.1 ml) was added dropwise at 0 ^oC. Subsequently, a solution of **58** (1.47 g, 3.77 mmol) in a mixture of DCM (6.4 ml) and toluene (6.4 ml) was very slowly added dropwise at 0 ^oC. The mixture was stirred, in the dark, at room temperature for 21 h. The reaction was diluted with DCM, filtered through Celite and washed with H_2O then $NaHCO_3$ (sat.). The organic layer was dried with Na_2SO_4 and

concentrated under reduced pressure. The residue was purified by flash chromatography on a silica gel column (Hex/EtOAc = 2:1→1:1) to give **60** (474 mg, 26%) as a white foam.

60 (470 mg, 0.65 mmol) was dissolved in a solution of TFA/H_2O (95:1, 8.0 ml) and stirred at room temperature for 2.5 h. The solvent was removed under reduced pressure by co-evaporation with toluene and the remaining residue was purified by flash chromatography on a silica gel column (Hex/EtOAc/AcOH = 1:1:0→1:1:0.2) to give **40** (400 mg, 92%) as a white foam. ^1H NMR (400 MHz, $CDCl_3$) δ 7.82 – 7.75 (m, 2H), 7.62 (d, *J* = 7.5 Hz, 2H), 7.46 – 7.37 (m, 2H), 7.33 (td, *J* = 7.4, 1.3 Hz, 2H), 5.81 (d, *J* = 6.6 Hz, 1H), 5.38 – 5.24 (m, 2H), 5.21 (s, 1H), 4.81 (s, 1H), 4.50 (d, *J* = 5.7 Hz, 1H), 4.43 (d, *J* = 7.1 Hz, 2H), 4.36 – 4.27 (m, 1H), 4.23 (t, *J* = 6.7 Hz, 1H), 4.12 (d, *J* = 12.5 Hz, 1H), 4.01 (d, *J* = 8.9 Hz, 1H), 3.90 (s, 1H), 3.53 (dd, *J* = 10.7, 5.3 Hz, 1H), 2.35 – 2.20 (m, 2H), 2.15 (s, 3H), 2.11 (s, 3H), 2.07 (d, *J* = 1.6 Hz, 3H), 2.01 (s, 3H). ^{13}C NMR (101 MHz, $CDCl_3$) δ 171.37 , 170.80 , 170.37 , 169.80 , 155.82 , 143.68 , 141.31 , 127.77 , 127.08 , 125.08 , 125.03 , 120.02 , 120.00 , 97.55 , 69.63 , 69.47 , 68.92 , 66.98 , 65.65 , 63.84 , 62.51 , 51.35 , 47.18 , 31.33 , 20.87 , 20.79 , 20.75 , 20.71. IR (NaCl, film): 3341, 3066, 2952, 1748, 1522, 1451, 1371, 1228, 1138, 1117, 1049, 980 cm^{-1}. HRMS (ESI) Calcd. for $C_{33}H_{37}NNaO_{14}$ $[M + Na]^+$ requires 694.2107, Found: 694.2103.

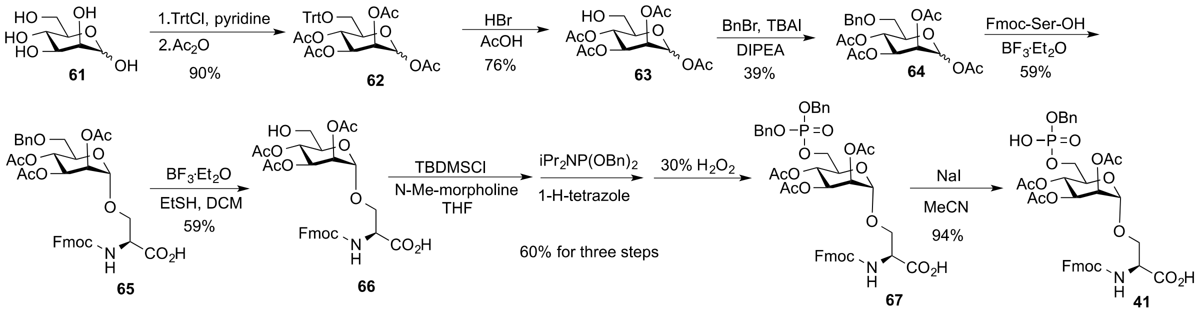

Synthesis of glycoamino acid 41. To a stirred solution of D-(+)-mannose **61** (5 g, 27.8 mmol) in pyridine (25 ml) trityl chloride (TrtCl) (8.5 g, 30.55 mmol) was added and the resulting mixture was stirred for 1.5 h at 40 °C. After cooling to 0 °C, Ac_2O (15 ml) was added to the mixture and the resulting solution was stirred overnight at room temperature. The reaction mixture was poured into ice water and extracted with DCM. The organic phase was dried over anhydrous Na_2SO_4, filtered and the filtrate was concentrated under reduced pressure. The resulting oil was purified by flash chromatography on a silica gel column (Hex/EtOAc = 5:1) to give **62** (14.71 g, 90%) as a white foam. Product matched the previously known spectra of **62**[3].

To a stirred solution of **62** (3 g, 5.08 mmol) in AcOH (10 ml) was added 33% HBr in AcOH (1.0 ml). The resulting mixture was stirred for 1 min. The Ph_3CBr formed was immediately removed by suction filtration. The filtrate was diluted with cold water and extracted with DCM. The organic layer was dried over Na_2SO_4, filtered and the filtrate was concentrated under reduced pressure. The resulting oil was purified by flash chromatography on a silica gel column (Hex/EtOAc =2:1→1:1) to give **63** (1.34 g, 76%) as a white foam. Product matched the

previously known spectra of **63**[3].

To a flask with **63** (23 g, 66 mmol) and tetra-n-butylammonium iodide (TBAI) (7.3 g, 19.8 mmol) was added DIPEA (45 ml, 264 mmol) and BnBr (31.6 ml, 264 mmol). The resulting mixture was stirred at 90 °C for 4 h. The reaction was diluted with DCM and washed with water. The organic phase was dried over Na_2SO_4, filtered and the filtrate was concentrated under reduced pressure. The resulting oil was purified by flash chromatography on a silica gel column (Hex/EtOAc =4:1→2:1) to give **64** (11.16 g, 39%) as an oil (α/β=2:1). ^{1}H-NMR (400 MHz, $CDCl_3$) δ 7.27-7.36 (m, 7.5H, H-Ph), 6.10 (d, *J* = 2.0 Hz, 1H, H-1α), 5.85 (d, *J* = 1.2 Hz, 0.5H, H-1β), 5.47 (dd, *J* = 3.2 Hz, 1.2 Hz, 0.5H, H-2β), 5.31-5.41 (m, 2.5H, H-3α, H-4α, H-4β), 5.25 (dd, *J* = 3.2 Hz, 2.0 Hz, 1H, H-2α), 5.11 (dd, *J* = 10.0 Hz, 3.2 Hz, 0.5H, H-3β), 4.46-4.59 (m, 3H, CH_2-Bn), 3.98-4.02 (m, 1H, H-5α), 3.73-3.77 (m, 0.5H, H-5β), 3.57-3.60 (m, 3H, H-6α, H-6β), 2.21 (s, 1.5H, CH_3-Acβ), 2.162 (s, 3H, CH_3-Acα), 2.155 (s, 3H, CH_3-Acα), 2.09 (s, 1.5H, CH_3-Acβ), 2.002 (s, 3H, CH_3-Acα), 1.997 (s, 1.5H, CH_3-Acβ), 1.92 (s, 3H, CH_3-Acα), 1.90 (s, 1.5H, CH_3-Acβ). ^{13}C-NMR (100 MHz, $CDCl_3$) δ 170.3, 170.1, 169.9, 169.8, 169.62, 169.58, 168.4, 168.2, 137.7, 137.6, 128.6, 128.4, 128.0, 127.9, 127.8, 127.7, 90.7, 90.5, 77.2, 74.5, 73.67, 73.61, 72.0, 70.9, 68.9, 68.8, 68.6, 68.4, 68.3, 66.4, 66.3, 20.9, 20.82, 20.79, 20.74, 20.68, 20.57. IR (NaCl, film): 3064, 3031, 2937, 2870, 1761, 1454, 1432, 1370, 1237, 1149, 1055, 972, 738, 701 cm^{-1}. HRMS (ESI) Calcd. for $C_{21}H_{26}NaO_{10}$ $[M + Na]^+$ requires 461.1419, Found: 461.1417.

To the solution of **64** (400 mg, 0.91 mmol) and Fmoc-Ser-OH (448 mg, 1.37 mmol) in MeCN (22 mL), $BF_3 \cdot OEt_2$ (0.42 ml, 2.74 mmol) was added[1]. The resulting mixture was stirred at room temperature for 24 h under argon. The solvent was removed under reduced pressure and the residue was diluted with EtOAc and washed with water. The organic layer was dried over Na_2SO_4, filtered and the filtrate was concentrated under reduced pressure. The resulting oil was purified by flash chromatography on a silica gel column (Hex/EtOAc/AcOH =2:1:0.3) to give **65** (379 mg, 59%) as a white foam. ^{1}H-NMR (400 MHz, $CDCl_3$) δ 7.74 (d, *J* = 7.6 Hz, 2H, H-Fmoc), 7.56-7.58 (m, 2H, H-Fmoc), 7.24-7.38 (m, 9H, H-Fmoc, H-Ph), 6.54 (d, *J* = 8.8 Hz, 1H, H-NH), 5.33-5.43 (m, 3H, H-2, H-3, H-4), 4.87 (s, 1H, H-1), 4.68 (brs, 1H, H-α), 4.49 (dd, *J* = 43.2 Hz, 12.0 Hz, 2H, CH_2-Bn), 4.03-4.44 (m, 6H, H-5, CH_2-β, CH_2-Fmoc, CH-Fmoc), 3.51 (s, 2H, H-6), 2.14 (s, 3H, CH_3-Ac), 2.00 (s, 3H, CH_3-Ac), 1.84 (s, 3H, CH_3-Ac). ^{13}C-NMR (100 MHz, $CDCl_3$) δ 170.7, 170.3, 169.8, 156.1, 143.8, 141.27, 141.24, 137.5, 128.8, 128.3, 127.92, 127.86, 127.7, 127.1, 125.3, 119.9, 98.3, 73.5, 70.1, 69.5, 68.4, 67.3, 66.7, 47.1, 43.9, 20.9, 20.8, 20.7. IR (NaCl, film): 3339, 3065, 1754, 1707, 1370, 1224, 1050, 740, 700 cm^{-1}. HRMS (ESI) Calcd. for $C_{37}H_{39}NNaO_{13}$ $[M + Na]^+$ requires 728.2314, Found: 728.2304.

The mixture of **65** (300 mg, 0.42 mmol), EtSH (2.1 ml) and $BF_3 \cdot OEt_2$ (523 μl, 3.40 mmol) in DCM (4.2 ml) was stirred for 6 h at room temperature under argon. The reaction was quenched with water and extracted with EtOAc. The organic phase was washed with brine, dried (Na_2SO_4), filtered and the filtrate was concentrated under reduced pressure. The resulting oil was purified

by flash chromatography on a silica gel column (Hex/EtOAc/AcOH =3:2:0.5→1:1:0.2) to give **66** (152 mg, 59%) as an oil. ^{1}H-NMR (400 MHz, CD_3OD) δ 7.82 (d, *J* = 7.6 Hz, 2H, H-Fmoc), 7.72-7.74 (m, 2H, H-Fmoc), 7.39-7.43 (m, 2H, H-Fmoc), 7.32-7.36 (m, 2H, H-Fmoc), 5.23-5.36 (m, 3H, H-2, H-3, H-4), 4.91 (s, 1H, H-1), 3.89-4.48 (m, 7H, H-5, H-α, CH_2-β, CH_2-Fmoc, CH-Fmoc), 3.66 (dd, *J* = 12.4 Hz, 2.4 Hz, 1H, H-6), 3.56 (dd, *J* = 12.4 Hz, 5.2 Hz, 1H, H-6), 2.12 (s, 3H, CH_3-Ac), 1.97 (s, 3H, CH_3-Ac), 1.95 (s, 3H, CH_3-Ac). ^{13}C-NMR (400 MHz, CD_3OD) δ 170.22, 170.17, 170.1, 157.0, 144.1, 143.9, 141.18, 141.12, 127.34, 127.32, 126.82, 126.79, 125.1, 124.9, 119.47, 119.45, 98.1, 71.0, 69.6, 69.5, 68.5, 66.8, 66.2, 60.5, 19.21, 19.19. IR (NaCl, film): 1755, 1706, 1370, 1227, 1084, 1047, 760, 740 cm^{-1}. HRMS (ESI) Calcd. for $C_{30}H_{33}NNaO_{13}$ $[M + Na]^+$ requires 638.1845, Found: 638.1844.

To a solution of **66** (150 mg, 0.24 mmol) in THF (1.5 ml) were added N-methyl-morpholine (27 μl, 0.24 mmol, dissolved in 0.4 ml THF) and tert-Butyldimethylchlorosilane (TBDMSCl) (36 mg, 0.24 mmol, dissolved in 0.5 ml THF)[4]. After stirring for 30 minutes, 1H-tetrazole (2.5 ml, 1.13 mmol, 0.45M in CH_3CN) and dibenzyl N,N-diisopropylphosphoramidite [$iPr_2NP(OBn)_2$] (166 μl, 0.5 mmol) were added. The reaction mixture was stirred for 3 h at room temperature, cooled to 0 ^{o}C, and then 30% H_2O_2 (aq., 64 μl, 0.64 mmol) was added. The resulting mixture was slowly warmed to room temperature over 30 minutes, saturated Na_2SO_3 (1.5 ml) was then added. After stirring vigorously for 30 minutes, the mixture was diluted with saturated $Na_2SO_{3,}$ extracted with EtOAc. The organic layer was dried over Na_2SO_4, filtered and the filtrate was concentrated under reduced pressure. The resulting oil was purified by flash chromatography on a silica gel column (Hex/EtOAc/AcOH =2:1:0.3→3:2:0.5) to give **67** (127 mg, 60%) as a white foam. ^{1}H-NMR (400 MHz, CD_3OD) δ 7.81 (d, *J* = 7.2 Hz, 2H, H-Fmoc), 7.69 (d, *J* = 7.6 Hz, 2H, H-Fmoc), 7.30-7.42 (m, 14H, H-Fmoc, H-Ph), 5.33-5.38 (m, 2H, H-3, H-4), 5.26 (d, *J* = 2.0 Hz, 1H, H-2), 5.04-5.12 (m, 4H, CH_2-Bn), 4.90 (s, 1H, H-1), 4.40-4.48 (m, 2H, H-α, CH-Fmoc), 4.23-4.31 (m, 2H, CH_2-Fmoc), 4.05-4.17 (m, 4H, H-5, H-6, CH_2-β), 3.90 (dd, *J* = 10.4 Hz, 6.0 Hz, 1H, H-6), 2.01 (s, 3H, CH_3-Ac), 1.96 (s, 3H, CH_3-Ac), 1.95 (s, 3H, CH_3-Ac). ^{13}C-NMR (400 MHz, CD_3OD) δ 170.04, 170,00, 169.91, 144.0, 143.8, 141.2, 135.8, 128.32, 128.30, 128.28, 127.81, 127.75, 127.4, 126.83, 126.80, 125.0, 119.5, 98.3, 69.55, 69.52, 69.49, 69.45, 69.4, 69.2, 68.4, 66.8, 65.6, 65.3, 54.7, 19.21, 19.16, 19.15. ^{31}P-NMR (400 MHz, CD_3OD) δ -1.77. IR (NaCl, film): 3065, 3035, 2360, 2343, 1756, 1718, 1521, 1371, 1246, 1220, 1011, 882, 740, 698 cm^{-1}. HRMS (ESI) Calcd. for $C_{44}H_{46}NNaO_{16}P$ $[M + Na]^+$ requires 898.2447, Found: 898.2438.

67 (120 mg, 0.14 mmol) was dissolved in CH_3CN (1.5 ml). To this solution was added NaI (42 mg, 0.28 mmol)[5]. The reaction was stirred at 45 ^{o}C for 12 h under argon. The reaction mixture was concentrated and dissolved in small amount EtOAc. Hexanes was added until white solid formed. The suspension was centrifuged and the resulting solid was dissolved in H_2O/CH_3CN=1:1. The resulting solution was frozen and lyophilized to give **41** (103 mg, 94%) as a white solid. ^{1}H-NMR (400 MHz, CD_3OD) δ 7.80 (d, *J* = 7.6 Hz, 2H, H-Fmoc), 7.69-7.72 (m, 2H, H-Fmoc), 7.22-7.41 (m, 9H, H-Fmoc, H-Ph), 5.26-5.36 (m, 3H, H-2, H-3, H-4), 4.91-4.95

(m, 2H, CH_2-Bn), 4.85 (d, J = 1.6 Hz, 1H, H-1), 4.41-4.46 (m, 1H, CH-Fmoc), 4.22-4.29 (m, 3H, H-α, CH_2-Fmoc), 3.83-4.13 (m, 5H, H-5, H-6, CH_2-β), 2.09 (s, 3H, CH_3-Ac), 1.95 (s, 3H, CH_3-Ac), 1.90 (s, 3H, CH_3-Ac). ^{13}C-NMR (400 MHz, CD_3OD) δ 170.3, 170.2, 170.1, 156.9, 144.1, 143.9, 141.17, 141.13, 138.5, 138.4, 128.2, 127.9, 127.3, 127.1, 126.9, 126.8, 125.1, 124.9, 119.5, 98.0, 69.8, 69.7, 69.5, 68.7, 66.79, 66.73, 66.1, 63.77, 63.72, 56.1, 19.30, 19.27, 19.24. ^{31}P-NMR (400 MHz, CD_3OD) δ 0.31. IR (NaCl, film): 3405, 3065, 2952, 1753, 1613, 1524, 1452, 1416, 1371, 1228, 1138, 1048, 868, 761, 740, 699 cm^{-1}. HRMS (ESI) Calcd. for $C_{37}H_{40}NNaO_{16}P$ [M + Na]$^+$ requires 808.1977, Found: 808.1980.

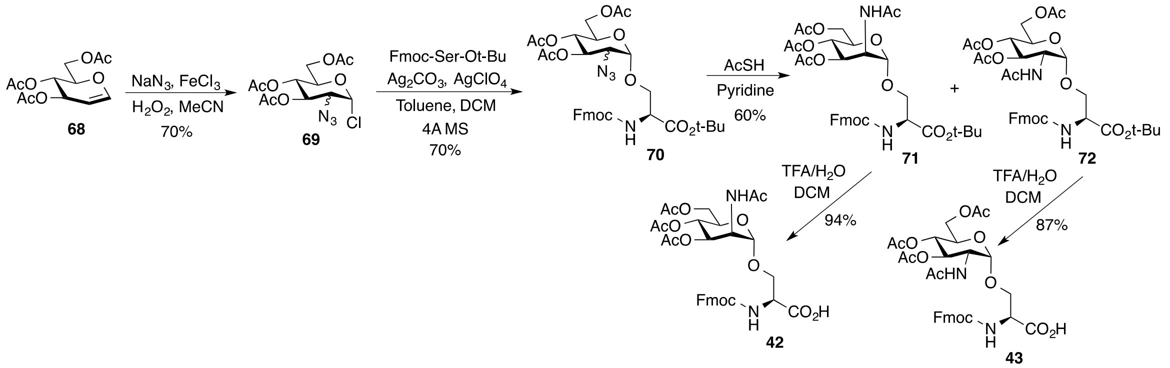

Synthesis of glycoamino acid 42 and 43. To a solution of Tri-O-acetyl-D-glucal **68** (2.3 g, 8.43 mmol) in MeCN (38 mL) at -30 °C was added $FeCl_3 \cdot 6H_2O$ (2.50 g, 9.27 mmol), NaN_3 (602.5 mg, 9.27 mmol) and H_2O_2 (30%, aq., 1.26 ml, 12.65 mmol) and the reaction was stirred at -30 °C for 6 h[6]. The mixture was diluted with Et_2O and washed with H_2O, $NaHCO_3$ (sat.), and brine. The organic layer was dried with Na_2SO_4 and concentrated under reduced pressure to give **69** (2.06 g, 70%) as a viscous oil. The product was used directly without further purification.

To a solution of Fmoc-Ser-OH (1.0 g, 3.05 mmol) in EtOAc (15.3 ml) was slowly added a solution of tert-Butyl 2,2,2-trichloroacetimidate (TBTA) (918 mg, 5.13 mmol) in cyclohexane (6.1 ml) over the course of 15 minutes with stirring at room temperature[7]. The mixture was allowed to stir at room temperature for 5 hours, then quenched with $NaHCO_3$ (sat., aq.) and extracted with EtOAc. The organic layers were combined, washed with H_2O, then brine, dried over Na_2SO_4, filtered and concentrated under reduced pressure. The residue was purified by flash chromatography on a silica gel column (Hex/EtOAc = 10:1→2:1) to yield Fmoc-Ser-Ot-Bu (643.6 mg, 55%) as a white solid.

A solution of Fmoc-Ser-Ot-Bu (2.03 g, 5.31 mmol), Ag_2CO_3 (2.44 g, 8.85 mmol), 4A MS (3.5 g) in toluene (12 ml) and DCM (18 ml) was stirred at 0 °C for 30 min[6]. Then, a solution of $AgClO_4$ (306.8 mg, 1.48 mmol) in toluene (6 mL) was added dropwise at 0 °C. Subsequently, a solution of **69** (2.06 g, 5.9 mmol) in a mixture of DCM (12 ml) and toluene (12 mL) was very slowly added dropwise at 0 °C. The mixture was stirred, in the dark, at room temperature for 24 h. The reaction was diluted with EtOAc, filtered through Celite and washed with H_2O then $NaHCO_3$ (sat.). The organic layer was dried with Na_2SO_4 and concentrated under reduced pressure. The

residue was purified by flash chromatography on a silica gel column (PE/EtOAc = 5:1→2:1) to give **70** (2.86 g, 70%) as a white foam.

70 (2.6 g, 3.73 mmol) was dissolved in a solution of pyridine (4 ml) and AcSH (8 ml) and stirred at room temperature for 40 hours[8]. The mixture was diluted with EtOAc and washed with HCl (1M, aq.), and $NaHCO_3$ (sat., aq.). The organic layers were dried with Na_2SO_4 and concentrated under reduced pressure. The residue was purified by flash chromatography on a silica gel column (DCM/EtOAc = 3:2→1:1→0:1) to give **71** (780 mg, 29%) and **72** (830 mg, 31%) as white foams.

71 (780 mg, 1.09 mmol) was dissolved in a solution of TFA (4 ml), H_2O (0.2 mL) and DCM (4 ml) and stirred at room temperature for 3 h. The solvent was removed under reduced pressure by co-evaporation with toluene and the remaining residue was purified by flash chromatography on a silica gel column (DCM:MeOH = 15:1→10:1) to give **42** (680 mg, 94%) as a white foam.

72 (830 mg, 1.16 mmol) was dissolved in a solution of TFA (4 ml), H_2O (0.2 ml) and DCM (3 ml) and stirred at room temperature for 3 h. The solvent was removed under reduced pressure by co-evaporation with toluene and the remaining residue was purified by flash chromatography on a silica gel column (DCM:MeOH = 15:1→10:1) to give **43** (670 mg, 87%) as a white foam.

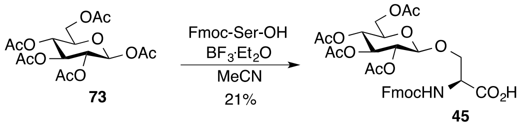

Synthesis of glycoamino acid 45. To the solution of penta-O-acetyl-β-D-glucopyranose **73** (1.0 g, 2.56 mmol) and Fmoc-Ser-OH (1.0 g, 3.07 mmol) in MeCN (30 ml), $BF_3{\cdot}OEt_2$ (1.0 mL, 7.68 mmol) was added. The resulting mixture was stirred at room temperature for 24 h under argon[1]. The solvent was removed under reduced pressure, and the resulting residue was diluted with EtOAc then washed with water. The organic layer was dried over Na_2SO_4, filtered and the filtrate was concentrated under reduced pressure. The resulting oil was purified by flash chromatography on a silica gel column (Hex/EtOAc/AcOH = 4:1:0.5→3:1:0.4→2:1:0.3) to give **45** (361 mg, 21%) as a white foam. Product matched the previously known spectra of **45**[9].

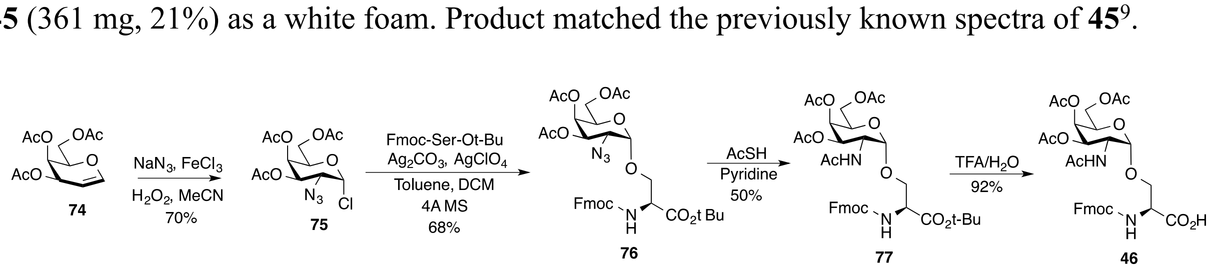

Synthesis of glycoamino acid 46. To a solution of 3,4,6-Tri-O-acetyl-D-galactal **74** (1.0 g, 3.67 mmol) in MeCN (30 ml) at -30 °C was added $FeCl_3{\cdot}6H_2O$ (0.79 g, 2.94 mmol), NaN_3 (263 mg, 4.04 mmol) and H_2O_2 (30%, aq., 0.42 ml, 4.04 mmol) and the reaction was stirred at -30 °C for 6 h[6]. The mixture was diluted with Et_2O and washed with H_2O, $NaHCO_3$, and brine. The organic layer was dried with Na_2SO_4 and concentrated under reduced pressure to give **75** (900 mg, 70%)

as a viscous oil. The product was used directly without further purification.

A solution of Fmoc-Ser-Ot-Bu (560 mg, 1.46 mmol), Ag_2CO_3 (600 mg, 2.19 mmol), 4A MS (1.7 g) in toluene (5 ml) and DCM (7.5 ml) was stirred at 0 ^oC for 30 minutes. Then, a solution of $AgClO_4$ (75 mg, 0.37 mmol) in toluene (2 ml) was added dropwise at 0^oC. Subsequently, a solution of **75** (900 mg, 2.58 mmol) in a mixture of DCM (3.75 ml) and toluene (3.75 ml) was very slowly added dropwise at 0 ^oC. The mixture was stirred, in the dark, at room temperature for 19 h. The reaction was diluted with DCM, filtered through Celite and washed with H_2O then $NaHCO_3$ (sat.). The organic layer was dried with Na_2SO_4 and concentrated under reduced pressure. The residue was purified by flash chromatography on a silica gel column (Tol/EtOAc = 10:1) to give **76** (692 mg, 68%) as a white foam.

76 (500 mg, 0.72 mmol) was dissolved in a solution of pyridine (0.8 ml) and AcSH (1.6 ml) and stirred at room temperature for 24 h. The mixture was diluted with DCM and washed with H_2O, HCl (1M, aq.), and $NaHCO_3$ (sat., aq.). The organic layers were dried with Na_2SO_4 and concentrated under reduced pressure. The residue was purified by flash chromatography on a silica gel column (Hex/EtOAc = 1:1→0:1) to give **77** (253 mg, 50%) as a white foam.

77 (240 mg, 0.34 mmol) was dissolved in a solution of TFA/H_2O (95:5, 4.0 ml) and stirred at room temperature for 2 h. The solvent was removed under reduced pressure by co-evaporation with toluene and the remaining residue was suspended in MeCN/H_2O (1:1), frozen and lyophilized to give a white solid. The solid was dissolved in DCM and purified by flash chromatography on a silica gel column (DCM:MeOH = 10:1→5:1) to give **46** (206 mg, 92%) as a white foam.

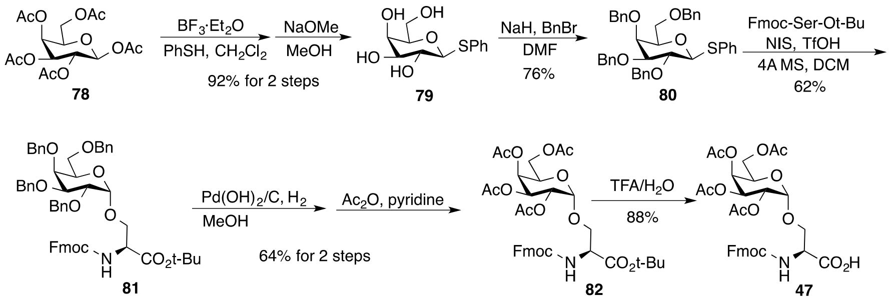

Synthesis of glycoamino acid 47. To a solution of β-D-galactose pentaacetate **78** (20.0 g, 51.3 mmol) in CH_2Cl_2 (100 ml) was added thiophenol (7.3 mL, 72.0 mmol). The resulting mixture was cooled to 0 °C. $BF_3 \cdot Et_2O$ (7.7 ml, 61.5 mmol) was then added dropwise and the reaction mixture was allowed to warm to room temperature. After being stirred at room temperature for 2 h, the mixture was diluted with CH_2Cl_2 (100 ml), washed with 2 M NaOH solution, H_2O, dried over $MgSO_4$ and concentrated under reduced pressure. The residue was dissolved in MeOH (100 ml) and MeONa (138 mg, 2.56 mmol) was added to the solution. The reaction was stirred at room temperature overnight, then neutralized with Amberlite IR-120 resin, filtered and

concentrated to give **79** (12.8 g, 92%) as a white foam[10].

To a suspension of NaH (9.4 g, 235 mmol, 60% in mineral oil) in DMF (150 mL) at 0 °C was added dropwise a solution of **79** (12.8 g, 47.0 mmol) in DMF (70 ml), which was followed by the addition of a solution of BnBr (27.8 mL, 235 mmol) in DMF (80 mL). The resulting mixture was stirred at room temperature overnight, then diluted with EtOAc, washed with H_2O, and concentrated under reduced pressure. The oily residue was purified by flash chromatography on a silica gel column (Hex/EtOAc = 6:1) to afford **80** (22.6 g, 76%) as a white solid[10].

To a solution of **80** (632 mg, 1.00 mmol) and Fmoc-Ser-Ot-Bu (421 mg, 1.10 mmol) in DCM (15 ml) at -30 °C were added 4A MS (300 mg), N-iodosuccinimide (NIS) (470 mg, 2.00 mmol) and trifluoromethanesulfonic acid (TfOH) (9 µL, 0.10 mmol). The resulting mixture was stirred at -30 °C for 10 min under argon before it was quenched with Na_2SO_3 (sat., aq.). The mixture was diluted with water and extracted with EtOAc. The organic layer was dried over Na_2SO_4, filtered and the filtrate was concentrated under reduced pressure. The resulting oil was purified by flash chromatography on silica gel (Hex/EtOAc = 5:1) to give **81** (561 mg, 62%) as a white foam. ^{1}H NMR (400 MHz, $CDCl_3$) δ 7.79 (d, J = 7.6 Hz, 2H), 7.63 (dd, J = 7.5, 4.0 Hz, 2H), 7.50 – 7.17 (m, 24H), 6.29 (d, J = 8.5 Hz, 1H), 4.99 (d, J = 11.3 Hz, 1H), 4.90 – 4.82 (m, $^1J_{CH}$ = 167.2 Hz, 3H), 4.79 (d, J = 11.7 Hz, 1H), 4.72 (d, J = 11.9 Hz, 1H), 4.62 (d, J = 11.4 Hz, 1H), 4.53 (d, J = 12.0 Hz, 1H), 4.49 – 4.40 (m, 2H), 4.40 – 4.33 (m, 2H), 4.29 – 4.19 (m, 2H), 4.11 (dd, J = 10.1, 3.7 Hz, 1H), 4.06 (t, J = 6.5 Hz, 1H), 4.01 (d, J = 2.8 Hz, 1H), 3.95 (dd, J = 10.1, 2.7 Hz, 1H), 3.87 (dd, J = 11.2, 3.0 Hz, 1H), 3.64 (dd, J = 9.3, 6.1 Hz, 1H), 3.55 (dd, J = 9.2, 6.6 Hz, 1H), 1.51 (s, 9H). ^{13}C NMR (101 MHz, $CDCl_3$) δ 169.15 , 156.14 , 143.97 , 143.95 , 141.28 , 138.71 , 138.59 , 138.57 , 137.98 , 128.42 , 128.39 , 128.37 , 128.29 , 127.96 , 127.76 , 127.71 , 127.67 , 127.64 , 127.58 , 127.51 , 127.10 , 125.27 , 119.95 , 99.52 , 82.36 , 78.74 , 74.96 , 74.80 , 73.48 , 73.41 , 73.09 , 70.73 , 70.02 , 68.93 , 67.06 , 55.24 , 47.16 , 28.04. IR (NaCl, film): 3340, 3064, 3031, 2978, 2929, 1725, 1497, 1453, 1369, 1347, 1248, 1155, 1100, 1057, 739, 697 cm^{-1}. HRMS (ESI) Calcd. for $C_{56}H_{59}NNaO_{10}$ $[M + Na]^+$ requires 928.4032, Found: 928.4026.

A solution of **81** (420 mg, 0.46 mmol) in MeOH (8 ml) was stirred with Pearlman's catalyst [$Pd(OH)_2$/C, 50 mg] under a hydrogen atmosphere at room temperature for 24 h. The reaction was filtered through Celite and the filtrate was concentrated under reduced pressure. The residue was dissolved in pyridine (2 ml) and Ac_2O (2 ml) was added dropwise. The resulting mixture was stirred at room temperature under argon overnight. The mixture was poured into ice-water and extracted with EtOAc. The organic layer was dried over Na_2SO_4, filtered and the filtrate was concentrated under reduced pressure. The resulting oil was purified by flash chromatography on silica gel column (Hex/EtOAc = 2:1) to give **82** (212 mg, 64%) as a white foam. ^{1}H NMR (400 MHz, $CDCl_3$) δ 7.75 (d, J = 7.5 Hz, 2H), 7.62 (d, J = 6.8 Hz, 2H), 7.39 (t, J = 7.3 Hz, 2H), 7.35 – 7.28 (m, 2H), 5.83 (d, J = 8.0 Hz, 1H), 5.45 (d, J = 2.4 Hz, 1H), 5.30 (dd, J = 10.9, 3.2 Hz, 1H), 5.15 (dd, J = 10.9, 3.5 Hz, 1H), 5.04 (d, J = 3.5 Hz, 1H), 4.46 – 4.35 (m, 3H), 4.24 (t, J = 7.1 Hz,

1H), 4.19 (t, J = 6.6 Hz, 1H), 4.10 – 4.02 (m, 2H), 3.98 (dd, J = 10.4, 2.8 Hz, 1H), 3.92 (dd, J = 10.6, 2.9 Hz, 1H), 2.13 (s, 3H), 2.05 (s, 3H), 1.99 (s, 3H), 1.95 (s, 3H), 1.48 (s, 9H). ^{13}C NMR (101 MHz, $CDCl_3$) δ 170.42 , 170.31 , 170.17 , 169.97 , 168.61 , 155.84 , 143.83 , 141.29 , 127.77 , 127.11 , 125.13 , 120.02 , 96.96 , 82.82 , 69.31 , 67.98 , 67.81 , 67.40 , 67.26 , 66.82 , 61.74 , 54.74 , 47.08 , 28.00 , 20.76 , 20.68 , 20.65 , 20.61. IR (NaCl, film): 3357, 3066, 2979, 1751, 1519, 1451, 1371, 1229, 1156, 1065, 761, 741 cm^{-1}. HRMS (ESI) Calcd. for $C_{36}H_{43}NNaO_{14}$ [M + Na]$^+$ requires 736.2576, Found: 736.2579.

Compound **82** (120 mg, 0.17 mmol) was dissolved in a TFA-water mixture (95:5, 1 ml) and stirred at room temperature for 2 h. The solvent was evaporated and the residue was co-evaporated with toluene to afford **47** (98 mg, 88%) as a white foam. ^{1}H NMR (400 MHz, $CDCl_3$) δ 7.76 (d, J = 7.5 Hz, 2H), 7.61 (t, J = 7.2 Hz, 2H), 7.40 (t, J = 7.5 Hz, 2H), 7.31 (t, J = 7.4 Hz, 2H), 6.50 (d, J = 8.7 Hz, 1H), 5.50 – 5.39 (m, 2H), 5.17 (d, $^1J_{CH}$ = 167.2 Hz, J = 3.5 Hz, 1H), 5.09 – 5.03 (m, 1H), 4.66 (d, J = 8.3 Hz, 1H), 4.39 (d, J = 7.1 Hz, 2H), 4.24 (d, J = 6.5 Hz, 2H), 4.12 – 4.01 (m, 2H), 4.01 – 3.91 (m, 2H), 2.14 (s, 3H), 2.03 (s, 3H), 2.01 (s, 3H), 1.91 (s, 3H). ^{13}C NMR (101 MHz, $CDCl_3$) δ 172.46 , 170.79 , 170.64 , 170.35 , 156.10 , 143.82 , 143.69 , 141.29 , 127.81 , 127.08 , 125.12 , 125.05 , 120.05 , 96.73 , 77.26 , 69.28 , 68.25 , 67.94 , 67.89 , 67.38 , 66.75 , 61.89 , 54.16 , 47.03 , 20.80 , 20.64 , 20.54 , 20.52. IR (NaCl, film): 3336, 3067, 2954, 1750, 1527, 1451, 1372, 1229, 1153, 1063, 761, 741 cm^{-1}. HRMS (ESI) Calcd. for $C_{32}H_{36}NNaO_{14}$ [M + Na]$^+$ requires 658.2131, Found: 658.2144.

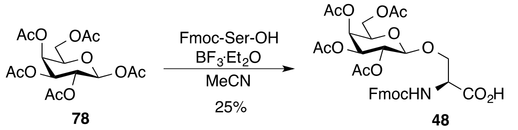

Synthesis of glycoamino acid 48. To the solution of β-D-Galactose pentaacetate **78** (1.0 g, 2.56 mmol) and Fmoc-Ser-OH (1.0 g, 3.07 mmol) in MeCN (30 ml), $BF_3 \cdot OEt_2$ (1.0 ml, 7.68 mmol) was added. The resulting mixture was stirred at room temperature for 24 h under argon. The solvent was removed under reduced pressure, and the resulting residue was diluted with EtOAc then washed with water. The organic layer was dried over Na_2SO_4, filtered and the filtrate was concentrated under reduced pressure. The resulting oil was purified by flash chromatography on a silica gel column (Hex/EtOAc/AcOH = 4:1:0.5→3:1:0.4→2:1:0.3) to give **48** (417.1 mg, 25%) as a white foam. Product matched the previously known spectra of **48**[9].

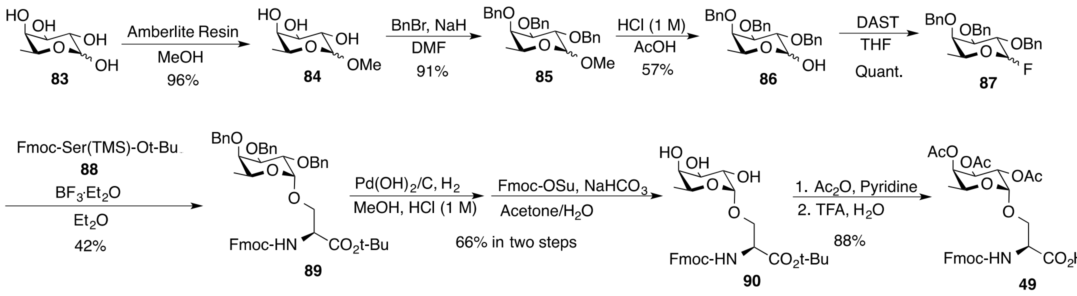

Synthesis of glycoamino acid 49. To a solution of L-fucose **83** (3.3 g, 20.1 mmol) in MeOH (33 ml) was added Amberlite IR120 Resin (MeOH pre-treated H^+ form, 5.3 g). The mixture was heated to reflux and allowed to stir at reflux for 3 h. The reaction was then filtered and concentrated under reduced pressure. The residue was purified by recrystallization from EtOH to give **84** (3.41 g, 96%) as an off-white solid[11].

To a solution of **84** (3.41 g, 19.1 mmol) in DMF (85 ml) was slowly added NaH (60% in oil, 5.04 g, 126.06 mmol) over the course of 20 min with stirring. The mixture was allowed to stir for 1 h before BnBr (6.77 ml, 57.3 mmol) was added. The mixture was stirred at room temperature overnight. The reaction was quenched by the slow addition of H_2O and extracted with EtOAc. The organic layers were combined, washed with $NaHCO_3$ (sat.) and brine, dried over Na_2SO_4 and concentrated under reduced pressure. The residue was purified by flash chromatography on a silica gel column (Hex/EtOAc = 12:1→6:1) to give **85** (7.84 g, 91%) as an oil[11].

85 (7.81 g, 17.4 mmol) was dissolved in AcOH (112.75 mL) and heated to 95 ^oC with stirring. HCl (aq., 1 M, 31.3 mL) was added and the mixture was allowed to stir at 95 ^oC for 2 h. The reaction was cooled to room temperature and extracted with $CHCl_3$. The organic layers were combined, washed first with ice-cold $NaHCO_3$ (sat.) until neutral, then brine, dried over Na_2SO_4, filtered and concentrated under reduced pressure. The residue was purified by flash chromatography on a silica gel column (Hex/EtOAc = 4:1→3:1) to give **86** (4.3 g, 57%) as a thick syrup[11].

86 (1.17 g, 2.68 mmol) was dissolved in THF (30 ml) and cooled to -30 ^oC. (Diethylamino)sulfur trifluoride (DAST) (0.37 mL, 2.81 mmol) was added at -30 ^oC and stirred for 5 min. The reaction was quenched with H_2O (4 ml) at -30 ^oC and stirred another 5 min, after which the cooling bath was removed and the reaction was diluted with EtOAc. The organic layer was washed with brine, dried over Na_2SO_4, filtered and concentrated under reduced pressure to almost-dryness. The residue was quickly purified by flash chromatography on a short silica gel column (Hex/EtOAc = 6:1) to give **87** (1.2 g, quant.) as a white oil[12].

To a solution of Fmoc-Ser-Ot-Bu (643.6 mg, 1.68 mmol) in DMF (3.5 ml) was added imidazole (456.8 mg, 6.71 mmol) and trimethylchlorosilane (TMSCl) (0.426 ml, 3.36 mmol). The mixture was allowed to stir at room temperature for 5 h. The reaction was quenched with brine and extracted with EtOAc. The organic layers were combined, washed with brine, dried over Na_2SO_4, filtered and concentrated under reduced pressure to give **88** (712.3 mg, 93%) as a thick oil. The product was used directly without further purification.

This and subsequent steps of the synthesis of compound **49** are based on a previously published procedure[13]. **87** (670.5 mg, 1.53 mmol) and **88** (698.9 mg, 1.53 mmol) were dissolved in Et_2O (23 ml) and cooled to -20 ^oC. $BF_3{\cdot}OEt_2$ (57 μl, 0.46 mmol) was added to the reaction and the

solution was allowed to stir at -20 °C for 1 h, after which the reaction was allowed to warm to 0 °C and stirred for another hour. The cooling bath was then removed and the reaction was stirred at room temperature for an additional 3 h. The reaction was quenched with $NaHCO_3$ (sat., aq.). The aqueous layer was extracted with Et_2O. The organic layers were then combined, washed with brine, dried over Na_2SO_4 and concentrated under reduced pressure. The residue was purified by flash chromatography on a silica gel column (Hex/EtOAc = 4:1) to give **89** (511.1 mg, 42%) as a hard white foam.

A solution of **89** (479.4 mg, 0.6 mmol) in EtOH (17 ml) and HCl (aq., 1M, 0.5 ml) was stirred with Pearlman's catalyst [$Pd(OH)_2$/C, 120 mg] under a hydrogen atmosphere at room temperature for 3 h. The reaction was filtered through Celite and the filtrate was neutralized with $NaHCO_3$ (sat., aq.) then concentrated under reduced pressure. The resulting residue was dissolved in H_2O (20 ml) and $NaHCO_3$ (200 mg), acetone (40 ml) and Fmoc-OSu (202 mg, 0.6 mmol) were added with vigorous stirring. The reaction was allowed to stir at room temperature for 1 h. The reaction was concentrated under reduced pressure, diluted with H_2O and extracted with $CHCl_3$. The organic layers were combined, dried with Na_2SO_4 and concentrated under reduced pressure. The residue was purified by flash chromatography on a silica gel column (DCM/MeOH = 50:1→25:2) to give **90** (210.6 mg, 66% in two steps from **89**) as a hard, white foam.

90 (201.4 mg, 0.38 mmol) was dissolved in a mixture of Ac_2O (1 ml) and pyridine (0.8 ml) and allowed to stir at room temperature for 17 h. The reaction was concentrated under reduced pressure and the residue was co-evaporated with toluene three times before being dissolved in a solution of TFA and H_2O (95:5, 1 ml) and stirred for an additional hour at room temperature. Solvent was removed by co-evaporating the reaction mixture with toluene under reduced pressure and the resulting residue was purified by flash chromatography on a silica gel column ($CHCl_3$/MeOH = 40:1→20:1) to give an oil, which was then dissolved in H_2O/CH_3CN=1:1. The solution was frozen and lyophilized to give **49** (184.2 mg, 88% over two steps from **90**) as a white solid. Product matched the previously known spectra of **49**[14].

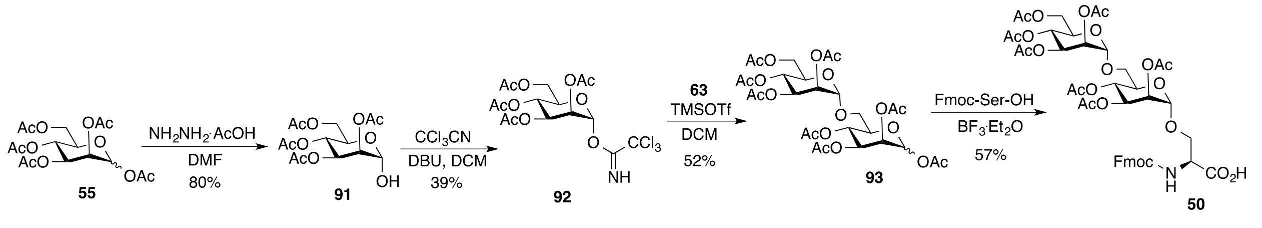

Synthesis of glycoamino acid 50. To a stirred solution of **55** (6.6 g, 16.92 mmol) in DMF (66 ml) was added N_2H_4·AcOH (1.87 g, 20.30 mmol). The resulting mixture was stirred for 4 h at room temperature under Ar. The reaction mixture was diluted with EtOAc, washed by water and brine. The organic layer was dried over Na_2SO_4, filtered and the filtrate was concentrated under reduced pressure. The resulting oil was purified by flash chromatography on a silica gel column

(Hex/EtOAc =1:1) to give **91** (4.69 g, 80%) as a syrup[15].

To a stirred solution of **94** (4.6 g, 13.21 mmol) in DCM (60 ml), CCl_3CN (13.21 ml, 132.10 mmol) and DBU (3.95 ml, 24.43 mmol) were added. After stirring overnight, the solvent was evaporated under reduced pressure and the residue was purified by flash chromatography on a silica gel column (Hex/EtOAc =1:1) to give **92** (2.54 g, 39%) as a syrup[15].

A solution of **63** (500 mg, 1.43 mmol) and **92** (776 mg, 1.58 mmol) in DCM (20 ml) was stirred with 4A molecular sieves (450 mg) under argon for 15 min. Then, a solution of Trimethylsilyl triflate (TMSOTf) (113 µl, 0.40 mmol) in DCM (2 ml) was added dropwise. The resulting mixture was stirred for 4 h at room temperature under argon. The reaction mixture was diluted with DCM and washed with sat. aq. $NaHCO_3$. The organic layer was dried over Na_2SO_4, filtered and the filtrate was concentrated under reduced pressure. The resulting oil was purified by flash chromatography on a silica gel column (Hex/EtOAc =2:1→3:2→1:1) to give **93** (504 mg, 52%) as a white foam.

To the solution of **93** (500 mg, 0.73 mmol) and Fmoc-Ser-OH (363 mg, 1.11 mmol) in MeCN (18 ml), $BF_3 \cdot OEt_2$ (0.34 ml, 2.22 mmol) was added. The resulting mixture was stirred at room temperature for 24 h under argon. The solvent was removed under reduced pressure, and the resulting residue was diluted with EtOAc then washed with water. The organic layer was dried over Na_2SO_4, filtered and the filtrate was concentrated under reduced pressure. The resulting oil was purified by flash chromatography on a silica gel column (Hex/EtOAc/AcOH = 2:1:0.3→3:2:0.5→1:1:0.2) to give **50** (390 mg, 57%) as a white foam. ^{1}H-NMR (400 MHz, Acetone-d6) δ 7.86 (d, J = 7.6 Hz, 2H, H-Fmoc), 7.74 (d, J = 7.6 Hz, 2H, H-Fmoc), 7.42 (t, J = 7.2 Hz, 2H, H-Fmoc), 7.34 (t, J = 7.4 Hz, 2H, H-Fmoc), 5.22-5.37 (m, 6H, H-2, H-3, H-4, H-2', H-3', H-4'), 4.96 (s, $^1J_{CH}$ = 176 Hz, H-1), 4.94 (s, $^1J_{CH}$ = 172 Hz, H-1'), 4.03-4.55 (m, 10H, H-α, CH_2-β, CH-Fmoc, CH_2-Fmoc, H-5, H-5', H-6'), 3.69-3.83 (m, 2H, H-6), 2.13 (s, 3H, CH_3-Ac), 2.10 (s, 3H, CH_3-Ac), 2.02 (s, 6H, CH_3-Ac), 2.01 (s, 3H, CH_3-Ac), 1.953 (s, 3H, CH_3-Ac), 1.947 (s, 3H, CH_3-Ac). ^{13}C-NMR (100 MHz, Acetone-d6) δ 169.9, 169.5, 169.4, 169.3, 144.3, 144.2, 141.18, 141.15, 127.6, 127.1, 125.4, 125.3, 119.9, 98.2, 97.5, 69.4, 69.2, 68.6, 66.6, 65.9, 62.3, 47.1, 19.86, 19.83, 19.80, 19.76. IR (NaCl, film): 3361, 2954, 1751, 1371, 1225, 1138, 1087, 1047, 761, 741 cm^{-1}. HRMS (ESI) Calcd. for $C_{44}H_{51}NNaO_{22}$ $[M + Na]^+$ requires 968.2795, Found: 968.2784.

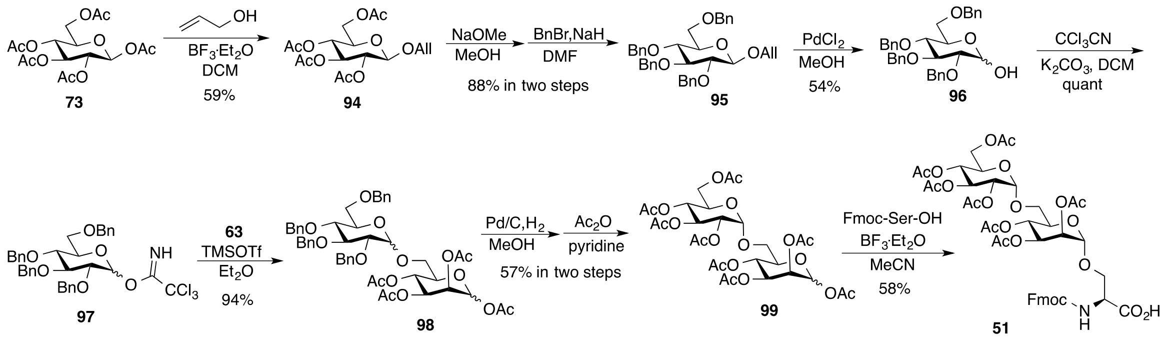

Synthesis of glycoamino acid 51. 73 (5 g, 12.80 mmol) was dissolved in DCM (30 ml) under argon. The stirred solution was cooled to 0 °C and $BF_3{\cdot}Et_2O$ (2.44 ml, 19.22 mmol) was added by syringe. After stirring for 10 min at 0 °C, allyl alcohol (1.31 ml, 19.22 mmol) was added. The ice bath was removed after completion of the addition and the reaction stirred at room temperature for overnight. The reaction was then cooled to 0 °C and quenched with $NaHCO_3$ (sat. aq.). After dilution with water, the organic layer was separated and aqueous layer was extracted with DCM. The combined organic layer was washed with brine, dried over Na_2SO_4, filtered and concentrated under reduced pressure. The product was purified by flash chromatography on a silica gel column (Hex/EtOAc =4:1→3:1→2:1) to give **94** (2.92 g, 59%) as a white solid[16].

To a stirred solution of **94** (2.9 g, 7.47 mmol) in MeOH (30 ml) was added NaOMe (20.5 mg, 0.38 mmol). After stirring for 1 h, the reaction was neutralized with Dowex H^+ and then filtered. The solvent was concentrated under reduced pressure to give a white foam (1.68g, 7.47 mmol). The product was dissolved in DMF (5 ml). The resulting solution was used directly in the next step without purification. To a suspension of NaH (60% in oil, 1.52 g, 38 mmol) in DMF (25 ml) was added dropwise the above solution at 0 °C under argon. The resulting mixture was stirred at 0 °C for 30 min and then BnBr (4.5 ml, 38 mmol) was added dropwise. The reaction was allowed to warm up to room temperature and stirred overnight. With caution, the reaction was quenched with water at 0°C. The resulting mixture was diluted with EtOAc and washed with water then brine. The organic layer was dried over Na_2SO_4, filtered and the filtrate was concentrated under reduced pressure. The resulting oil was purified by flash chromatography on a silica gel column (Hex/EtOAc =20:1→15:1→10:1) to give **95** (3.8 g, 88% over two steps) as a white solid[17].

A solution of **95** (1.8 g, 3.10 mmol) and $PdCl_2$ (109 mg, 0.62 mmol) in MeOH (20 ml) was stirred vigorously at room temperature overnight. The reaction was diluted with diethyl ether and filtered through Celite. The solvent was concentrated under reduced pressure and the residue was purified by flash chromatography on a silica gel column (Hex/EtOAc =4:1→2:1) to give **96** (900 mg, 54%) as a white solid[17].

A solution of **96** (900 mg, 1.66 mmol), CCl_3CN (1.72 ml, 21.6 mmol) and K_2CO_3 (1.15 g, 8.3 mmol) in DCM (22 ml) was stirred vigorously at room temperature under argon overnight. The reaction was filtered through Celite and the filtrate was concentrated under reduced pressure to

give **97** (1.21 g, 100%) as a white foam. The product was used directly in the next step without purification.

A solution of **63** (293 mg, 0.84 mmol) and **97** (690 mg, 1.01 mmol) in diethyl ether (20 ml) was stirred with 4A MS (700 mg) under argon for 1 h. The reaction was cooled to -40 °C and TMSOTf (76 µl, 0.42 mmol) was added. The resulting mixture was allowed to warm up to room temperature slowly and stirred for 4 h. The reaction was quenched with Et_3N (500 µl) and stirred for 10 additional minutes, then filtered. The filtrate was concentrated under reduced pressure and the residue was purified by flash chromatography on a silica gel column (Hex/EtOAc =5:1→2:1→1:1) to give **98** (688 mg, 94%) as a white foam.

A solution of **98** (680 mg, 0.78 mmol) in MeOH (25 ml) was stirred at room temperature under a hydrogen atmosphere for 24 h in the presence of 10% Pd/C (200 mg). The reaction was filtered through Celite and the filtrate was concentrated under reduced pressure. The residue was dissolved in pyridine (3 ml) and Ac_2O (1 ml) was added dropwise. The resulting mixture was stirred at room temperature under argon overnight. The mixture was slowly poured into ice-water and extracted with DCM. The organic layer was dried over Na_2SO_4, filtered and the filtrate was concentrated under reduced pressure. The resulting oil was purified by flash chromatography on a silica gel column (Hex/EtOAc =2:1→3:2) to give first **99** (304 mg, 57%). ^{1}H-NMR (400 MHz, $CDCl_3$) δ 6.06 (d, *J* = 2.0 Hz, 1H, H-1), 5.47 (dd, *J* = 10.0 Hz, 9.2 Hz, 1H, H-3'), 5.38 (dd, *J* = 10.0 Hz, 3.2 Hz, H-3), 5.31 (t, *J* = 10.0 Hz, 1H, H-4), 5.27 (dd, *J* = 3.6 Hz, 2.0 Hz, 1H, H-2), 5.07-5.12 (m, 2H, H-1', H4'), 4.86 (dd, *J* = 10.0 Hz, 3.6 Hz, 1H, H-2'), 4.26-4.30 (m, 1H, H-6'), 4.09-4.13 (m, 2H, H-5', H-6'), 4.03-4.07 (m, 1H, H-5), 3.79 (dd, *J* = 11.2 Hz, 6.0 Hz, H-6), 3.62 (dd, *J* = 10.8 Hz, 2.8 Hz, H-6), 2.21 (s, 3H, CH_3-Ac), 2.20 (s, 3H, CH_3-Ac), 2.11 (s, 3H, CH_3-Ac), 2.102, (s, 3H, CH_3-Ac), 2.099 (s, 3H, CH_3-Ac), 2.07 (s, 3H, CH_3-Ac), 2.04 (s, 3H, CH_3-Ac), 2.03 (s, 3H, CH_3-Ac). ^{13}C-NMR (100 MHz, $CDCl_3$) δ 170.7, 170.2, 169.98, 169.97, 169.8, 169.7, 169.6, 168.1, 95.3, 90.3, 71.1, 70.9, 69.9, 68.8, 68.30, 68.27, 67.3, 67.1, 66.3, 61.8, 20.84, 20.75, 20.72, 20.69, 20.66, 20.62. IR (NaCl, film): 1751, 1370, 1223, 1149, 1040, 977 cm^{-1}. HRMS (ESI) Calcd. for $C_{28}H_{38}NaO_{19}$ $[M + Na]^+$ requires 701.1900, Found: 701.1902.

To the solution of **99** (400 mg, 0.59 mmol) and Fmoc-Ser-OH (290 mg, 0.88 mmol) in MeCN (15 mL), $BF3{\cdot}OEt_2$ (0.27 ml, 1.77 mmol) was added. The resulting mixture was stirred at room temperature for 24h under argon. The solvent was removed under reduced pressure. The resulting residue was diluted with EtOAc, and then washed with water. The organic layer was dried over Na_2SO_4, filtered and the filtrate was concentrated under reduced pressure. The resulting oil was purified by flash chromatography on a silica gel column (Hex/EtOAc/AcOH =2:1:0.3→3:2:0.5→1:1:0.2) to give **51** (321 mg, 58%) as a white foam. ^{1}H-NMR (400 MHz, $CDCl_3$) δ 7.76 (d, *J* = 7.6 Hz, 2H, H-Fmoc), 7.63 (d, *J* = 6.4 Hz, 2H, H-Fmoc), 7.40 (t, *J* = 7.4 Hz, 2H, H-Fmoc), 7.31 (t, *J* = 7.6 Hz, 2H, H-Fmoc), 5.92 (d, *J* = 8.4 Hz, 1H, H-NH), 5.44 (t, *J* = 9.8 Hz, 1H, H-3'), 5.34 (dd, *J* = 10.0 Hz, 3.6 Hz, 1H, H-3), 5.27-5.29 (m, 1H, H-2), 5.18 (t, *J* =

10.2 Hz, 1H, H-4), 5.10 (d, J = 4.0 Hz, 1H, H-1'), 5.03 (t, J = 9.4 Hz, 1H, H-4'), 4.86 (dd, J = 10.0 Hz, 3.6 Hz, 1H, H-2'), 4.83 (s, 1H, H-1), 4.67-4.72 (m, 1H, H-α), 4.42 (d, J = 7.6 Hz, 2H, CH_2-Fmoc), 4.25 (t, J = 7.0 Hz, 1H, CH-Fmoc), 4.07-4.15 (m, 5H, H-β, H-5, H-5', H-6'), 3.90-3.94 (m, 1H, H-β), 3.79 (dd, J = 10.8 Hz, 6.8 Hz, 1H, H-6), 3.53 (dd, J = 10.8 Hz, 2.8 Hz), 2.14 (s, 3H, CH_3-Ac), 2.08 (s, 3H, CH_3-Ac), 2.06 (s, 3H, CH_3-Ac), 2.05 (s, 3H, CH_3-Ac), 2.01 (s, 3H, CH_3-Ac), 2.00 (s, 3H, CH_3-Ac), 1.99 (s, 3H, CH_3-Ac). ^{13}C-NMR (100 MHz, $CDCl_3$) δ 170.77, 170.75, 170.6, 170.3, 170.0, 169.9, 167.7, 156.1, 143.81, 143.77, 141.3, 127.7, 127.10, 127.08, 125.2, 120.0, 98.0, 95.4, 77.2, 70.7, 70.4, 69.9, 69.2, 69.0, 68.5, 68.4, 67.4, 67.3, 67.1, 66.6, 61.9, 54.1, 47.1, 20.80, 20.79, 20.77, 20.74, 20.70, 20.64, 20.58. IR (NaCl, film): 3355, 3065, 2952, 1751, 1521, 1452, 1370, 1223, 1139, 1042, 763, 741 cm^{-1}. HRMS (ESI) Calcd. for $C_{44}H_{51}NNaO_{22}$ [M + Na]$^+$ requires 968.2795, Found: 968.2793.

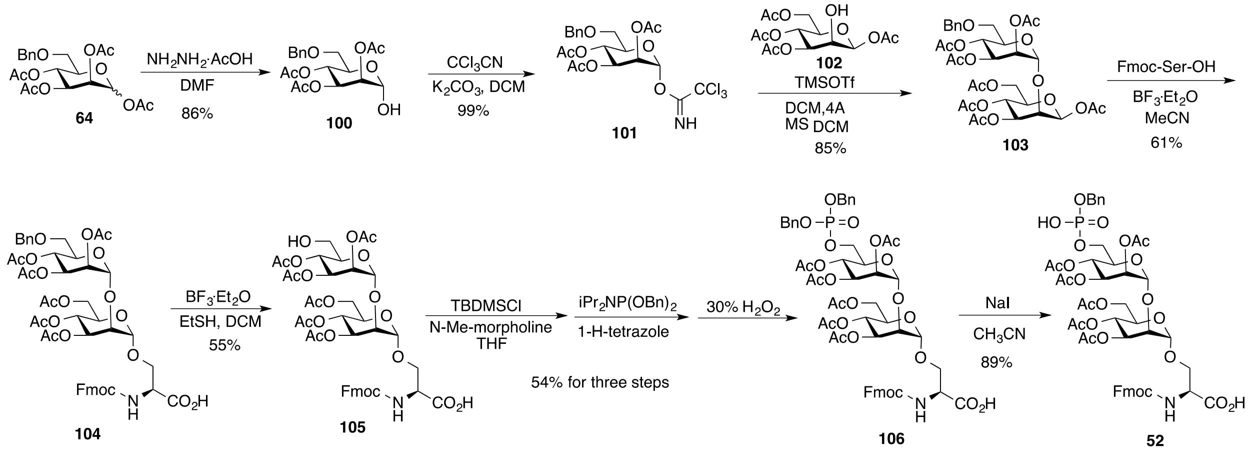

Synthesis of glycoamino acid 52. To a solution of **64** (4 g, 9.13 mmol) in DMF (37 ml) was added N_2H_4·AcOH (1.0 g, 10.96 mmol). The resulting mixture was stirred for 4 h at room temperature under argon. The reaction was diluted with EtOAc and washed with water and brine. The organic layer was dried over anhydrous Na_2SO_4, filtered and the filtrate was concentrated under reduced pressure. The resulting oil was purified by flash chromatography on a silica gel column (Hex/EtOAc =2:1→3:2) to give **100** (3.11 g, 86%) as a white foam. ^{1}H-NMR (400 MHz, $CDCl_3$) δ 7.27-7.36 (m, 5H, H-Ph), 5.40 (dd, J = 10.0 Hz, 3.2 Hz, 1H, H-3), 5.19-5.26 (m, 3H, H-1, H-2, H-4), 4.54 (dd, J = 25.2 Hz, 12.0 Hz, 2H, CH_2-Bn), 4.17-4.22 (m, 1H, H-5), 3.57 (dd, J = 10.8 Hz, 6.4 Hz, 1H, H-6), 3.49 (dd, J = 10.4 Hz, 2.4 Hz, 1H, H-6), 3.27 (brs, 1H, H-OH), 2.14 (s, 3H, CH_3-Ac), 1.99 (s, 3H, CH_3-Ac), 1.93 (s, 3H, CH_3-Ac). ^{13}C-NMR (100 MHz, $CDCl_3$) δ 170.2, 170.0, 169.9, 137.5, 128.4, 128.1, 127.8, 92.1, 73.6, 70.0, 69.6, 69.1, 68.8, 66.9, 20.9, 20.72, 20.69. IR (NaCl, film): 3423, 2937, 2871, 1751, 1454, 1433, 1372, 1227, 1078, 1051, 739, 701 cm^{-1}. HRMS (ESI) Calcd. for $C_{19}H_{24}NaO_9$ [M + Na]$^+$ requires 419.1313, Found: 419.1320.

100 (3.1 g, 7.83 mmol) in DCM (100 ml) was stirred vigorously with CCl_3CN (8.10 ml, 101.73 mmol) and K_2CO_3 (5.40 g, 39.13 mmol) at room temperature under argon overnight. The reaction mixture was filtered through celite and the filtrate was concentrated under reduced pressure to give **101** (4.39 g, 99%) as a syrup. The product was used directly to the next step

without further purification. ^{1}H-NMR (400 MHz, $CDCl_3$) δ 8.75 (s, 1H, H-NH), 7.27-7.35 (m, 5H, H-Ph), 6.29 (d, *J* = 2.0 Hz, 1H, H-1), 5.38-5.50 (m, 3H, H-2, H-3, H-4), 4.54 (dd, *J* = 40.4 Hz, 12.0 Hz, 2H, CH_2-Bn), 4.14-4.18 (m, 1H, H-5), 3.60 (d, *J* = 4.0 Hz, 2H, H-6), 2.18 (s, 3H, CH_3-Ac), 2.00 (s, 3H, CH_3-Ac), 1.93 (s, 3H, CH_3-Ac). ^{13}C-NMR (100 MHz, $CDCl_3$) δ 169.9, 169.8, 169.6, 137.7, 128.3, 127.9, 127.7, 94.7, 73.5, 72.5, 69.0, 68.5, 67.9, 66.1, 20.8, 20.69, 20.65. IR (NaCl, film): 3323, 2917, 2869, 1751, 1678, 1454, 1432, 1369, 1246, 1159, 1089, 1050, 976, 944, 836, 798, 737, 701 cm^{-1}. HRMS (ESI) Calcd. for $C_{21}H_{24}Cl_3NNaO_9$ $[M + Na]^+$ requires 562.0409, Found: 562.0406.

The 1,3,4,6-Tetra-O-acetyl-β-D-mannopyranose **102** was prepared as reported in the literature[1]. A solution of **102** (1.46 g, 4.20 mmol) and **101** (3 g, 5.58 mmol) in DCM (45 ml) was stirred with 4A MS (1.5 g) under argon for 30 min. The reaction was cooled to -30 ^oC and a solution of TMSOTf (234 μl, 1.26 mmol) in DCM (2 ml) was added dropwise. After stirring at -30 ^oC for 15 min, the reaction mixture was allowed to warm up to room temperature slowly and stirred for 5 h. The reaction was quenched with Et_3N (3 ml), stirred for an additional 10 minutes and then filtered. The filtrate was concentrated under reduced pressure and the residue was purified by flash chromatography on a silica gel column (Hex/EtOAc =3:2→1:1) to give **103** (2.6 g, 85%) as a white foam. ^{1}H-NMR (400 MHz, $CDCl_3$) δ 7.27-7.35 (m, 5H, H-Ph), 5.78 (d, *J* = 1.2 Hz, 1H, H-1), 4.99 (dd, *J* = 10.0 Hz, 3.2 Hz, 1H, H-3'), 5.29-5.41 (m, 3H, H-2', H-4, H-4'), 5.12 (dd, *J* = 9.6 Hz, 2.8 Hz, 1H, H-3), 5.00 (d, *J* = 2.0 Hz, 1H, H-1'), 4.50 (dd, *J* = 34.0 Hz, 8.0 Hz, 2H, CH_2-Bn), 4.36-4.41 (m, 1H, H-5'), 4.26 (dd, *J* = 12.4 Hz, 4.8 Hz, 1H, H-6), 4.16-4.20 (m, 2H, H-2, H-6), 3.76-3.80 (m, 1H, H-5), 3.55 (d, *J* = 4.0 Hz, 2H, H-6'), 2.14 (s, 3H, CH_3-Ac), 2.12 (s, 3H, CH_3-Ac), 2.10 (s, 3H, CH_3-Ac), 2.06 (s, 3H, CH_3-Ac), 2.03 (s, 3H, CH_3-Ac), 2.02 (s, 3H, CH_3-Ac), 1.92 (s, 3H, CH_3-Ac). ^{13}C-NMR (100 MHz, $CDCl_3$) δ 171.0, 170.3, 169.92, 169.85, 169.6, 169.3, 168.7, 137.6, 128.4, 127.9, 127.8, 98.4, 90.9, 74.6, 73.7, 73.1, 72.2, 70.2, 70.0, 69.3, 68.4, 67.1, 65.8, 61.8, 21.0, 20.77, 20.74, 20.70, 20.6, 20.5. IR (NaCl, film): 3064, 2939, 2869, 1751, 1454, 1433, 1370, 1221, 1055, 933, 737, 702 cm^{-1}. HRMS (ESI) Calcd. for $C_{33}H_{42}NaO_{18}$ $[M + Na]^+$ requires 749.2264, Found: 749.2261.

To the solution of **103** (2.5 g, 3.44 mmol) and Fmoc-Ser-OH (1.69 g, 5.16 mmol) in MeCN (80 mL), $BF_3{\cdot}OEt_2$ (1.6 ml, 10.33 mmol) was added. The resulting mixture was stirred at room temperature for 24 h under argon. The solvent was removed under reduced pressure; the resulting residue was diluted with EtOAc and washed with water. The organic layer was dried (Na_2SO_4), filtered and the filtrate was concentrated under reduced pressure. The resulting oil was purified by flash chromatography on a silica gel column (Hex/EtOAc/AcOH =2:1:0.3→3:2:0.5) to give **104** (2.10 g, 61%) as a white foam. ^{1}H-NMR (400 MHz, $CDCl_3$) δ 7.77 (d, *J* = 7.6 Hz, 2H, H-Fmoc), 7.64 (d, *J* = 7.2 Hz, 2H, H-Fmoc), 7.41 (t, *J* = 7.4 Hz, 2H, H-Fmoc), 7.30-7.36 (m, 7H, H-Fmoc, H-Ph), 5.81 (d, *J* = 8.0 Hz, 1H, H-NH), 5.22-5.40 (m, 4H, H-2', H-3, H-3', H-4'), 5.09 (t, *J* = 10.0 Hz, 1H, H-4), 5.02 (d, *J* = 0.8 Hz, 1H, H-1), 4.98 (d, *J* = 1.6 Hz, 1H, H-1'), 4.65 (dd, *J* = 56.4 Hz, 12.4 Hz, 2H, CH_2-Bn), 4.50-4.52 (m, 1H, H-α), 3.90 (d, *J* = 6.8 Hz, 2H, CH_2-

Fmoc), 3.96-4.27 (m, 8H, H-2, H-5, H5', H-6, CH-Fmoc, CH_2-β), 3.66 (dd, J = 10.4 Hz, 7.2 Hz, 1H, H-6'), 3.56 (dd, J = 10.4 Hz, 2.8 Hz, 1H, H-6'), 2.12 (s, 6H, CH_3-Ac), 2.09 (s, 3H, CH_3-Ac), 2.02 (s, 3H, CH_3-Ac), 2.00 (s, 3H, CH_3-Ac), 1.93 (s, 3H, CH_3-Ac). ^{13}C-NMR (100 MHz, $CDCl_3$) δ 171.0, 170.7, 170.4, 170.0, 169.8, 169.4, 155.9, 143.79, 143.76, 141.3, 136.0, 128.6, 128.5, 128.4, 127.8, 127.1, 125.20, 125.17, 120.0, 100.2, 98.2, 74.5, 73.8, 70.23, 70.17, 70.15, 69.7, 69.6, 69.0, 68.0, 67.4, 67.3, 66.7, 62.1, 54.7, 47.1, 20.9, 20.69, 20.67, 20.65. IR (NaCl, film): 3338, 3065, 2952, 1751, 1521, 1452, 1370, 1225, 1136, 1047, 760 cm^{-1}. HRMS (ESI) Calcd. for $C_{49}H_{55}NNaO_{21}$ $[M + Na]^+$ requires 1016.3159, Found: 1016.3148[1].

The mixture of **104** (1.9 g, 1.91 mmol), EtSH (7.5 ml) and $BF_3{\cdot}OEt_2$ (2.42 ml, 15.30 mmol) in DCM (15 ml) was stirred for 6 h at room temperature under argon. The reaction was quenched with water and extracted with EtOAc. The organic phase was washed with brine, dried (Na_2SO_4), filtered and the filtrate was concentrated under reduced pressure. The resulting oil was purified by flash chromatography on a silica gel column (Hex/EtOAc/AcOH =1:1:0.2→2:3:0.5) to give **105** (945 mg, 55%) as a white foam. ^1H-NMR (400 MHz, CD_3OD) δ 7.82 (d, J = 7.6 Hz, 2H, H-Fmoc), 7.71-7.74 (m, 2H, H-Fmoc), 7.39-7.43 (m, 2H, H-Fmoc), 7.32-7.36 (m, 2H, H-Fmoc), 5.42-5.41 (m, 5H, H-2', H-3, H-3', H-4, H-4'), 5.15 (d, J = 2.0 Hz, 1H, H-1), 5.00 (d, J = 1.6 Hz, 1H, H-1'), 4.47 (dd, J = 10.0 Hz, 6.4 Hz, 2H, CH_2-Fmoc), 3.93-4.34 (m, 9H, H-2, H-5, H-5', H-6, H-α, CH_2-β, CH-Fmoc), 3.66 (dd, J = 12.4 Hz, 2.4 Hz, 1H, H-6'), 3.59 (dd, J = 12.0 Hz, 5.6 Hz, 1H, H-6'), 2.14 (s, 3H, CH_3-Ac), 2.11 (s, 3H, CH_3-Ac), 2.052 (s, 3H, CH_3-Ac), 2.046 (s, 3H, CH_3-Ac), 2.00 (s, 3H, CH_3-Ac), 1.97 (s, 3H, CH_3-Ac). ^{13}C-NMR (100 MHz, CD_3OD) δ 171.3, 170.5, 170.22, 170.20, 170.1, 170.0, 157.0, 144.0, 143.8, 141.19, 141.15, 127.41, 127.38, 126.84, 126.80, 125.0, 124.8, 119.53, 119.51, 99.2, 99.0, 76.9, 71.6, 70.3, 69.7, 69.0, 68.8, 68.4, 66.8, 66.4, 66.1, 54.8, 19.4, 19.29, 19.27, 19.23, 19.18. IR (NaCl, film): 3350, 3065, 1751, 1371, 1229, 1046, 740 cm^{-1}. HRMS (ESI) Calcd. for $C_{42}H_{49}NNaO_{21}$ $[M + Na]^+$ requires 926.2690, Found: 926.2693.

To a solution of **105** (850 mg, 0.94 mmol) in THF (5.875 ml) were added N-methyl-morpholine (106 μl, 0.94 mmol, dissolved in 1.57 ml THF) and TBDMSCl (141 mg, 0.94 mmol, dissolved in 1.96 ml THF). After stirring for 30 minutes, 1H-tetrazole (9.79 ml, 4.42 mmol, 0.45M in CH_3CN) and $iPr_2N(OBn)_2$ (650 μl, 1.98 mmol) were added. The reaction mixture was stirred for 3 h at room temperature, cooled to 0 °C, and then 30% H_2O_2 (aq., 250 μl, 2.49 mmol) was added. The resulting mixture was slowly warmed to room temperature over 30 min, saturated Na_2SO_3 (6 ml) was then added. After stirring vigorously for 30 min, the mixture was diluted with saturated Na_2SO_3, extracted with EtOAc. The organic layer was dried over Na_2SO_4, filtered and the filtrate was concentrated under reduced pressure. The resulting oil was purified by flash chromatography on a silica gel column (Hex/EtOAc/AcOH =1:1:0.2) to give **106** (586 mg, 54%) as a white foam. ^1H-NMR (400 MHz, CD_3OD) δ 7.81 (d, J = 7.6 Hz, 2H, H-Fmoc), 7.69-7.72 (m, 2H, H-Fmoc), 7.31-7.42 (m, 14H, H-Fmoc, H-Ph), 5.27-5.41 (m, 5H, H-2', H-3, H-3', H-4, H-4'), 5.04-5.13 (m, 5H, H-1, CH_2-Bn), 4.94 (d, J = 1.2 Hz, 1H, H-1'), 4.04-4.45 (m, 12H, H-2, H-

5, H-5’, H-6, H-6’, H-α, CH_2-β, CH_2-Fmoc, CH-Fmoc), 3.92 (dd, J = 10.4 Hz, 6.0 Hz, 1H, H-6), 2.09 (s, 3H, CH_3-Ac), 2.04 (s, 6H, CH_3-Ac), 2.00 (s, 6H, CH_3-Ac), 1.97 (s, 3H, CH_3-Ac). ^{13}C-NMR (100 MHz, CD_3OD) δ 171.2, 170.4, 170.03, 170.99, 169.91, 169.86, 158.3, 157.0, 144.0, 143.8, 141.19, 141.14, 135.74, 135.67, 128.39, 128.36, 128.33, 128.31, 127.9, 127.8, 127.39, 127.37, 126.84, 126.80, 125.0, 124.9, 119.5, 99.1, 98.8, 77.1, 70.2, 69.57, 69.55, 69.51, 69.50, 69.45, 68.8, 68.4, 66.8, 65.93, 65.85, 65.80, 65.6, 61.7, 19.4, 19.28, 19.25, 19.20, 19.19, 19.13. ^{31}P-NMR (400 MHz, CD_3OD) δ -1.51. IR (NaCl, film): 2956, 1750, 1452, 1370, 1225, 1046, 740 cm^{-1}. HRMS (ESI) Calcd. for $C_{56}H_{62}NNaO_{24}P$ $[M + Na]^+$ requires 1186.3292, Found: 1186.3287[4].

106 (480 mg, 0.41 mmol) was dissolved in CH_3CN (4.4 ml). To this solution was added NaI (124 mg, 0.82 mmol). The reaction was stirred at 45 °C for 12 h under argon. The reaction mixture was concentrated and dissolved in small amount EtOAc. Hexane was added until white solid formed. The suspension was centrifuged and the resulting solid was dissolved in H_2O/CH_3CN=1:1. The resulting solution was frozen and lyophilized to give **52** (392 mg, 89%) as a white solid. ^{1}H-NMR (400 MHz, CD_3OD) δ 7.81 (d, J = 7.6 Hz, 2H, H-Fmoc), 7.71-7.74 (m, 2H, H-Fmoc), 7.22-7.42 (m, 9H, H-Fmoc, H-Ph), 5.24-5.40 (m, 5H, H-2’, H-3, H-3’, H-4, H-4’), 5.14 (d, J = 1.6 Hz, 1H, H-1), 4.93-5.00 (m, 2H, CH_2-Bn), 4.86 (d, J = 2.0 Hz, 1H, H-1’), 3.93-4.47 (m, 13H, H-2, H-5, H-5’, H-6, H-6’, H-α, CH_2-β, CH_2-Fmoc, CH-Fmoc), 2.13 (s, 3H, CH_3-Ac), 2.10 (s, 3H, CH_3-Ac), 2.02 (s, 3H, CH_3-Ac), 1.99 (s, 3H, CH_3-Ac), 1.98 (s, 3H, CH_3-Ac), 1.97 (s, 3H, CH_3-Ac). ^{13}C-NMR (100 MHz, CD_3OD) δ 171.3, 170.3, 170.2, 170.1, 170.0, 143.9, 141.2, 127.9, 127.3, 127.0, 126.9, 126.8, 119.46, 119.43, 99.0, 98.9, 76.7, 70.4, 70.2, 69.6, 69.0, 68.6, 66.8, 66.7, 66.2, 66.1, 63.9, 61.7, 60.2, 55.4, 19.4, 19.28, 19.27, 19.25, 19.22, 19.15. ^{31}P-NMR (400 MHz, CD_3OD) δ 0.28. IR (NaCl, film): 3431, 2954, 1749, 1623, 1452, 1370, 1227, 1081, 1046, 740 cm^{-1}. HRMS (ESI) Calcd. for $C_{49}H_{56}NNaO_{24}P$ $[M + Na]^+$ requires 1096.2822, Found: 1096.2830[5].

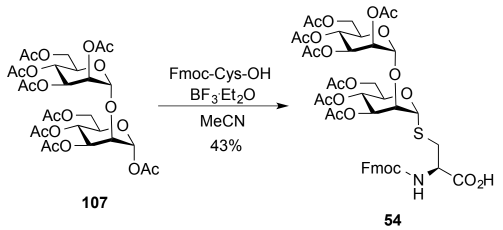

Synthesis of glycoamino acid 54. 107 was prepared as reported in the literature [1]. To the solution of **107** (678 mg, 1.0 mmol) and Fmoc-Cys-OH (514 mg, 1.5 mmol) in MeCN (20 ml), $BF_3{\cdot}OEt_2$ (0.46 mL, 3.0 mmol) was added[18]. The resulting mixture was stirred at room temperature for 19 h under argon. The solvent was removed under reduced pressure, and the resulting residue was diluted with EtOAc then washed with water. The organic layer was dried (Na_2SO_4), filtered and the filtrate was concentrated under reduced pressure. The resulting oil was purified by flash chromatography on a silica gel column (Hex/EtOAc/AcOH = 2:1:0.3→3:2:0.5) to give **54** (414 mg, 43%) as a white foam. ^{1}H-NMR (400 MHz, $CDCl_3$) δ 7.76 (d, J = 7.6 Hz,

2H, H-Fmoc), 7.61 (d, J = 7.2 Hz, 2H, H-Fmoc), 7.40 (t, J = 7.4 Hz, 2H, H-Fmoc), 7.32 (t, J = 7.4 Hz, 2H, H-Fmoc), 6.05 (d, J = 7.2 Hz, 1H, NH), 5.48 (s, 1H, H-1), 5.40 (dd, 3H, J = 10.0, 2.7 Hz, H-3'), 5.31-5.36 (m, 2H, H-4, H-4'), 5.17-5.23 (m, 2H, H-2', H-3), 4.92 (d, J = 1.2 Hz, 1H, H-1'), 4.64 (s, 1H, H-α), 4.27-4.45 (m, 5H, H-5', H-6, CH_2- Fmoc), 4.22 (t, 1H, J = 7.0 Hz, CH-Fmoc), 4.13-4.17 (m, 4H, H-2, H-5, H-6'), 3.24 (dd, 2H, J = 66.0, 13.6 Hz, CH_2-β), 2.14 (s, 3H, CH_3-Ac), 2.13 (s, 3H, CH_3-Ac), 2.12 (s, 3H, CH_3-Ac), 2.08 (s, 3H, CH_3-Ac), 2.04 (s, 3H, CH_3-Ac), 2.02 (s, 3H, CH_3-Ac), 2.01 (s, 3H, CH_3-Ac). ^{13}C-NMR (100 MHz, $CDCl_3$) δ 171.9, 171.1, 170.3, 169.8, 169.7, 169.6, 169.4, 155.8, 143.74, 143.72, 141.3, 127.8, 127.1, 125.1, 120.0, 99.2, 83.6, 78.4, 77.2, 70.2, 69.62, 69.56, 69.1, 68.4, 67.3, 66.6, 66.2, 62.4, 62.1, 53.6, 47.1, 33.6, 20.9, 20.8, 20.67, 20.65, 20.63. IR (NaCl, film): 3350, 2956, 1749, 1370, 1228, 1045, 741 cm^{-1}. HRMS (ESI) Calcd. for $C_{44}H_{51}NNaO_{21}S$ $[M + Na]^+$ requires 984.2567, Found: 984.2556.

III. SYNTHESIS AND CHARACTERIZATION OF CBM GLYCOFORMS

General procedure for the synthesis of unglycosylated CBM variants. The crude peptide was prepared using the previously reported protocol[18]. 16 mg of the crude peptide was dissolved in 80 ml of folding buffer (0.2 M Tris-acetate, 0.33 mM oxidized glutathione, 2.6 mM reduced glutathione, pH 8.2) and stirred at room temperature for 12 h under a helium atmosphere. The solution was then concentrated to a small volume (around 6 ml) using 3 kDa cut-off centrifugal filter units (Amicon) before RP-HPLC purification. The RP-HPLC purification was performed on a Versagrad Preparation-HPLC system using a semi-preparative C18 column. The products were detected by UV absorption at 275 nm. After HPLC purification with a linear gradient of 20→40% MeCN in H_2O over 30 min, the fractions were collected and checked by ESI+ MS. The pure fractions were combined and lyophilized to give the desired product as a white solid.

General procedure for the synthesis of glycosylated CBM variants. The crude glycopeptide was prepared using the previously reported protocol[18]. 16 mg of the crude peptide was dissolved in 1 ml of hydrazine solution (hydrazine/H_2O, 5/100, v/v) and stirred at room temperature for 30 min under helium. The reaction was quenched with 2 ml of acetic acid solution (AcOH/H_2O, 5/100, v/v). The resulting mixture was diluted to 80 mL with folding buffer (0.2 M Tris-acetate, 0.33 mM oxidized glutathione, 2.6 mM reduced glutathione, pH 8.2, 80 ml) and stirred at room temperature for 12 h under a helium atmosphere. The solution was then concentrated to a small volume (around 6 ml) using 3 kDa cut-off centrifugal filter units (Amicon) before RP-HPLC purification. The RP-HPLC purification was performed on a Versagrad Preparation-HPLC system using a semi-preparative C18 column. The products were detected by UV absorption at 275 nm. After HPLC purification with a linear gradient of 20→40% MeCN in H_2O over 30 min, the fractions were collected and checked by ESI+ MS. The pure fractions were combined and lyophilized to afford the desired product as a white solid.

LC-MS analysis of purified CBM variants. LC-MS was performed under two flow rates with C4 column: (1) 0.5 ml/min with a linear gradient of 15% to 35% acetonitrile in water over 3 min and (2) 0.3 ml/min with a linear gradient of 15% to 35% acetonitrile in water over 5 min.

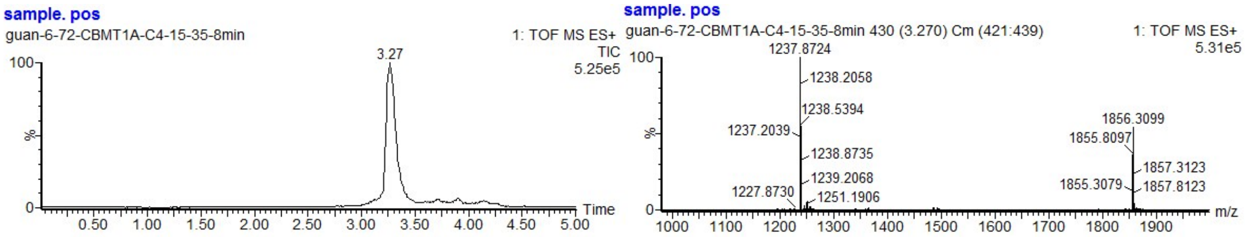

Figure S2. LC-MS trace and ESI-MS of purified CBM **4** (yield: 28%). LC-MS condition: 0.3 mL/min, 15%-35% MeCN in H_2O over 5 min. MS (ESI) Calcd for **4** $C_{158}H_{233}N_{43}O_{53}S_4$ $[M+2H]^{2+}$ m/z = 1855.2949, $[M+3H]^{3+}$ m/z = 1237.1992.

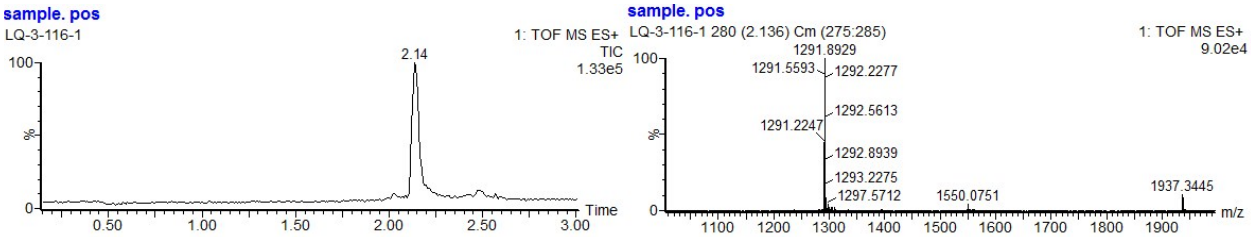

Figure S3. LC-MS trace and ESI-MS of purified CBM **5** (yield: 18%). LC-MS condition: 0.5 ml/min, 15%-35% MeCN in H_2O over 3 min. MS (ESI) Calcd for **5** $C_{164}H_{243}N_{43}O_{58}S_4$ $[M+2H]^{2+}$ m/z = 1936.3135, $[M+3H]^{3+}$ m/z =1291.2090.

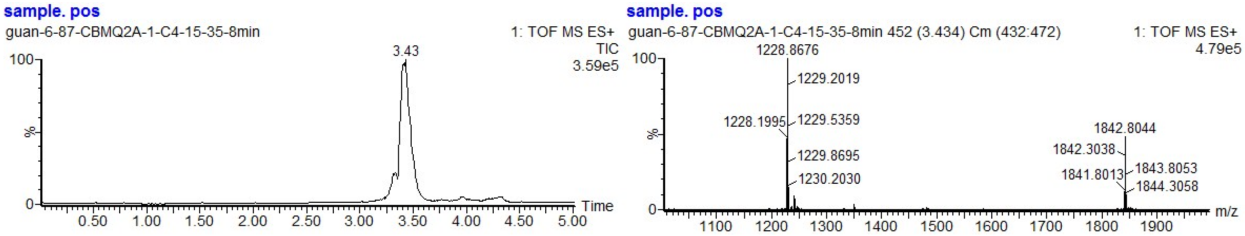

Figure S4. LC-MS trace and ESI-MS of purified CBM **6** (yield: 30%). LC-MS condition: 0.3 mL/min, 15%-35% MeCN in H_2O over 5 min. MS (ESI) Calcd for **6** $C_{157}H_{232}N_{42}O_{53}S_4$ $[M+2H]^{2+}$ m/z = 1841.7894, $[M+3H]^{3+}$ = m/z 1228.1955.

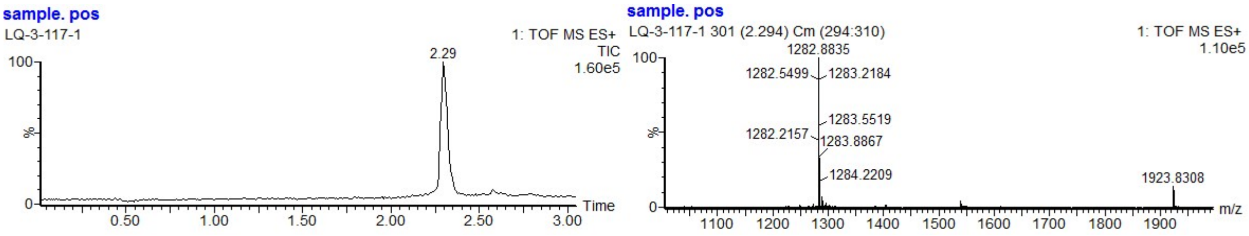

Figure S5. LC-MS trace and ESI-MS of purified CBM **7** (yield: 17%). LC-MS condition: 0.5 mL/min, 15%-35% MeCN in H_2O over 3 min. MS (ESI) Calcd for **7** $C_{163}H_{242}N_{42}O_{58}S_4$ $[M+2H]^{2+}$ m/z = 1922.8081, $[M+3H]^{3+}$ m/z = 1282.2054.

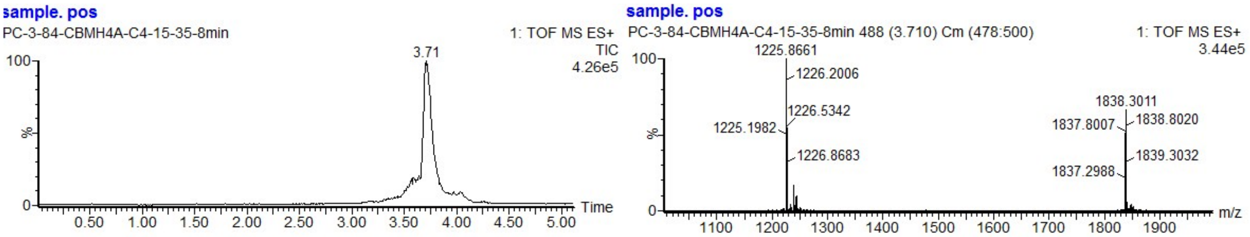

Figure S6. LC-MS trace and ESI-MS of purified CBM **8** (yield: 15%). LC-MS condition: 0.3 ml/min, 15%-35% MeCN in H_2O over 5 min. MS (ESI) Calcd for **8** $C_{156}H_{233}N_{41}O_{54}S_4$ $[M+2H]^{2+}$ m/z = 1837.2893, $[M+3H]^{3+}$ m/z = 1225.1954.

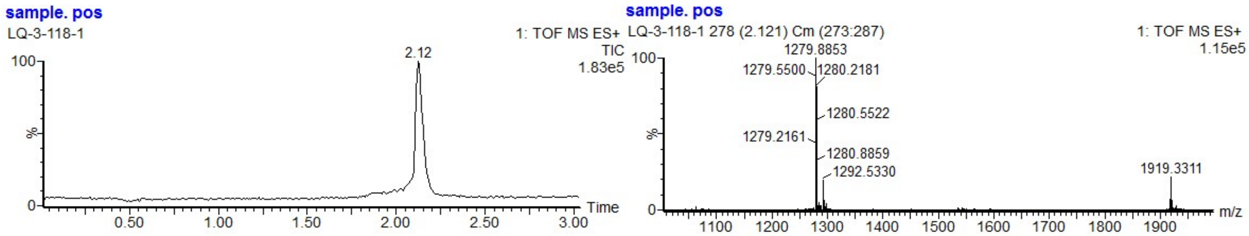

Figure S7. LC-MS trace and ESI-MS of purified CBM **9** (yield: 13%). LC-MS condition: 0.5 ml/min, 15%-35% MeCN in H_2O over 3 min. MS (ESI) Calcd for **9** $C_{162}H_{243}N_{41}O_{59}S_4$ $[M+2H]^{2+}$ m/z = 1918.3079, $[M+3H]^{3+}$ m/z = 1279.2053.

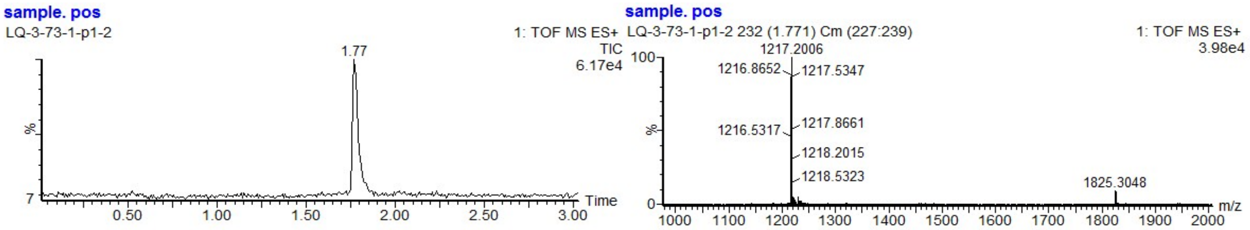

Figure S8. LC-MS trace and ESI-MS of purified CBM **10** (yield: 12%). LC-MS condition: 0.5 ml/min, 15%-35% MeCN in H_2O over 3 min. MS (ESI) Calcd for **10** $C_{153}H_{231}N_{43}O_{53}S_4$ $[M+2H]^{2+}$ m/z = 1824.2793, $[M+3H]^{3+}$ m/z = 1216.5195.

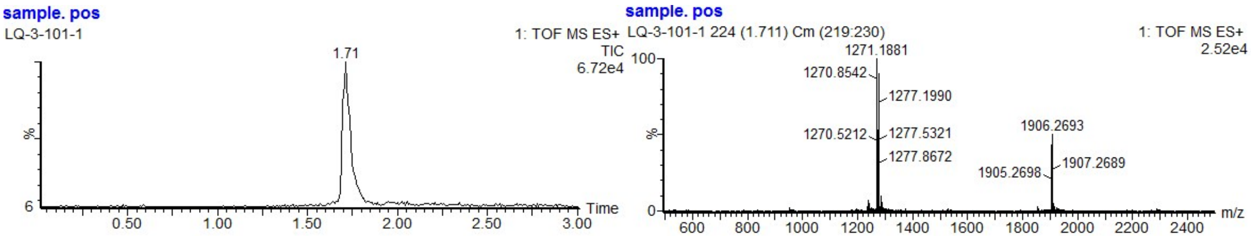

Figure S9. LC-MS trace and ESI-MS of purified CBM **11** (yield: 13%). LC-MS condition: 0.5 ml/min, 15%-35% MeCN in H_2O over 3 min. MS (ESI) Calcd for **11** $C_{159}H_{241}N_{43}O_{58}S_4$ $[M+2H]^{2+}$ m/z = 1905.3057, $[M+3H]^{3+}$ m/z = 1270.5371.

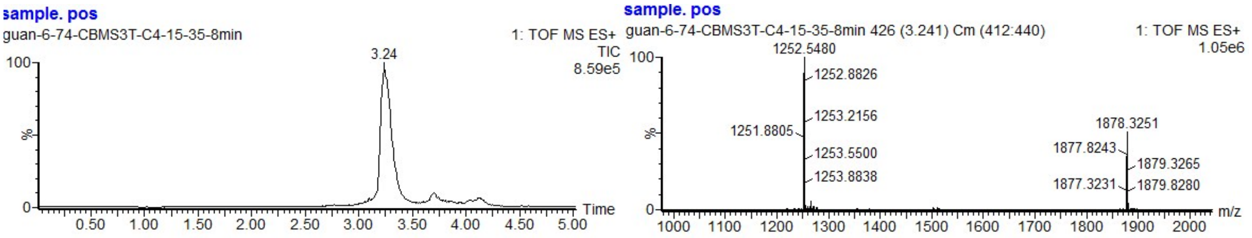

Figure S10. LC-MS trace and ESI-MS of purified CBM **12** (yield: 23%). LC-MS condition: 0.3 ml/min, 15%-35% MeCN in H_2O over 5 min. MS (ESI) Calcd for **12** $C_{160}H_{237}N_{43}O_{54}S_4$ $[M+2H]^{2+}$ m/z = 1877.3080, $[M+3H]^{3+}$ m/z = 1251.8746.

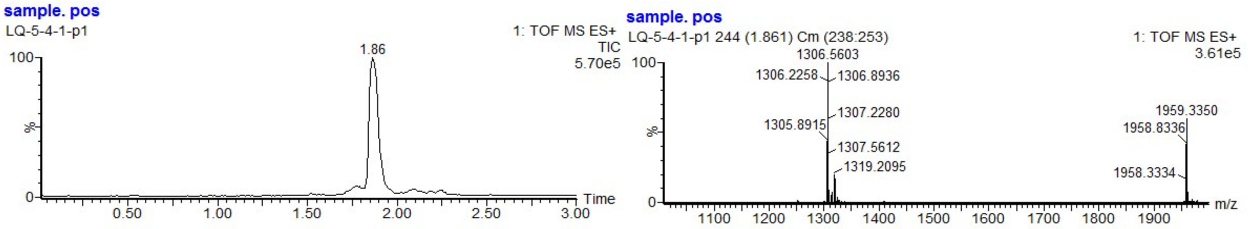

Figure S11. LC-MS trace and ESI-MS of purified CBM **13** (yield: 16%). LC-MS condition: 0.5 ml/min, 15%-35% MeCN in H_2O over 3 min. MS (ESI) Calcd for **13** $C_{166}H_{247}N_{43}O_{59}S_4$ $[M+2H]^{2+}$ m/z = 1958.3266, $[M+3H]^{3+}$ m/z = 1305.8844.

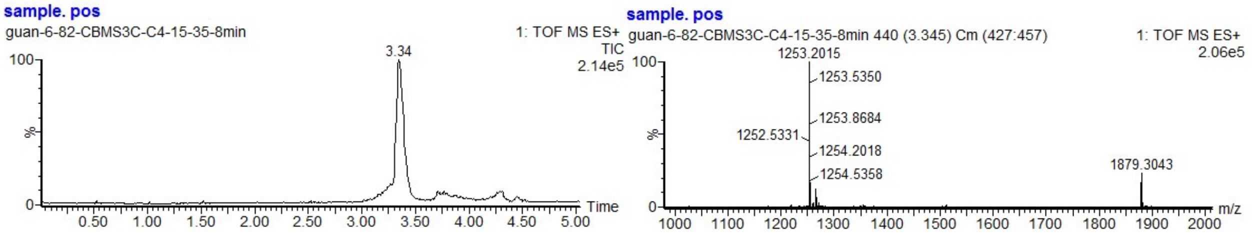

Figure S12. LC-MS trace and ESI-MS of purified CBM **14** (yield: 13%). LC-MS condition: 0.3 ml/min, 15%-35% MeCN in H_2O over 5 min. MS (ESI) Calcd for **14** $C_{159}H_{235}N_{43}O_{53}S_5$ $[M+2H]^{2+}$ m/z = 1878.2887, $[M+3H]^{3+}$ m/z = 1252.5284.

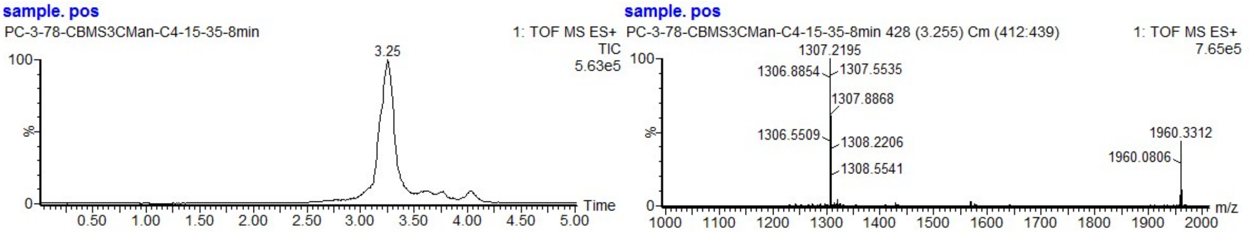

Figure S13. LC-MS trace and ESI-MS of purified CBM **15** (yield: 16%). LC-MS condition: 0.3 ml/min, 15%-35% MeCN in H_2O over 5 min. MS (ESI) Calcd for **15** $C_{165}H_{245}N_{43}O_{58}S_5$ $[M+2H]^{2+}$ m/z = 1959.3151, $[M+3H]^{3+}$ m/z = 1306.5460.

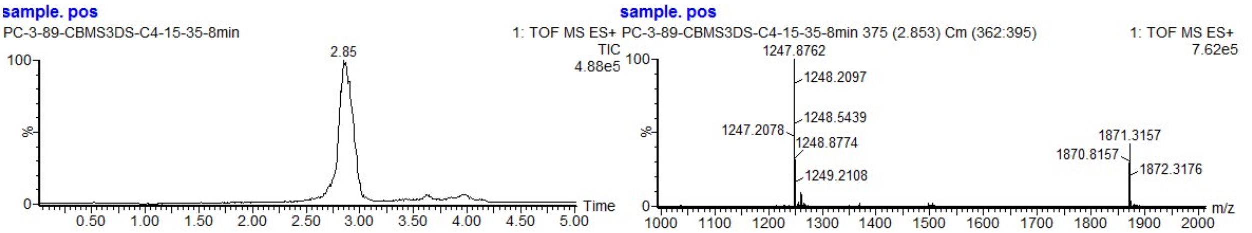

Figure 14. LC-MS trace and ESI-MS of purified CBM **16** (yield: 22%). LC-MS condition: 0.3 ml/min, 15%-35% MeCN in H_2O over 5 min. MS (ESI) Calcd for **16** $C_{159}H_{235}N_{43}O_{54}S_4$ $[M+2H]^{2+}$ m/z = 1870.3002, $[M+3H]^{3+}$ m/z = 1247.2027.

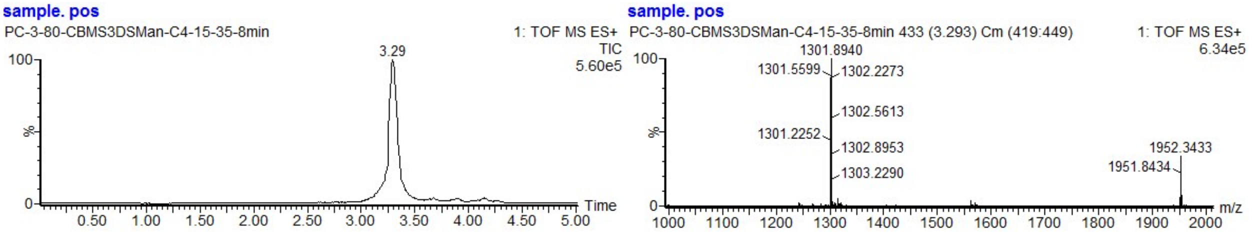

Figure S15. LC-MS trace and ESI-MS of purified CBM **17** (yield: 8%). LC-MS condition: 0.3 mL/min, 15%-35% MeCN in H_2O over 5 min. MS (ESI) Calcd for **17** $C_{165}H_{245}N_{43}O_{59}S_4$ $[M+2H]^{2+}$ m/z = 1951.3188 $[M+3H]^{3+}$ m/z = 1301.2125.

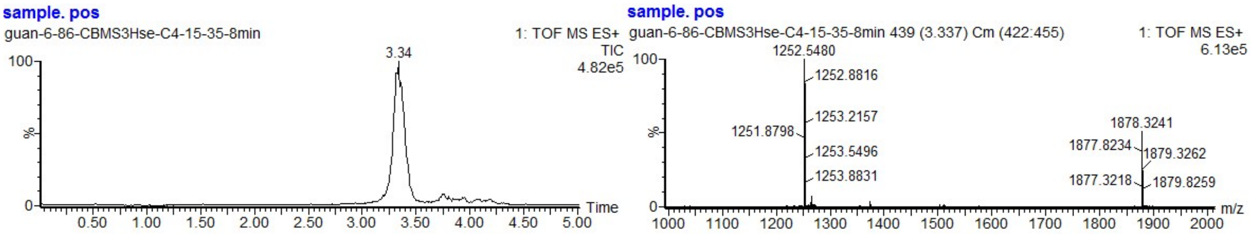

Figure S16. LC-MS trace and ESI-MS of purified CBM **18** (yield: 27%). LC-MS condition: 0.3 ml/min, 15%-35% MeCN in H_2O over 5 min. MS (ESI) Calcd for **18** $C_{160}H_{237}N_{43}O_{54}S_4$ $[M+2H]^{2+}$ m/z = 1877.3080, $[M+3H]^{3+}$ m/z = 1251.8746.

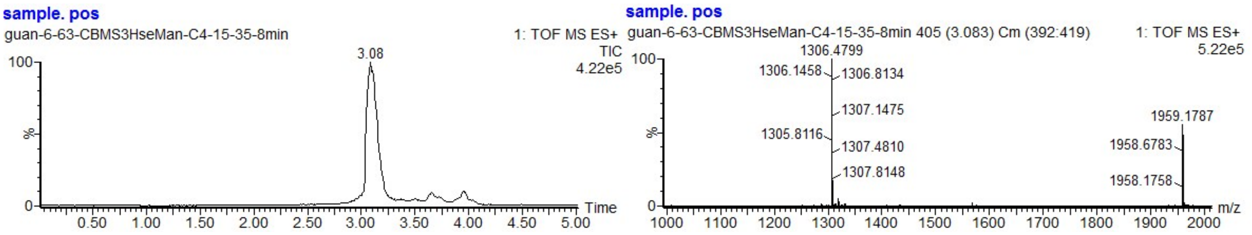

Figure S17. LC-MS trace and ESI-MS of purified CBM **19** (yield: 28%). LC-MS condition: 0.3 ml/min, 15%-35% MeCN in H_2O over 5 min. MS (ESI) Calcd for **19** $C_{166}H_{247}N_{43}O_{59}S_4$ $[M+2H]^{2+}$ m/z = 1958.3344, $[M+3H]^{3+}$ m/z = 1305.8922.

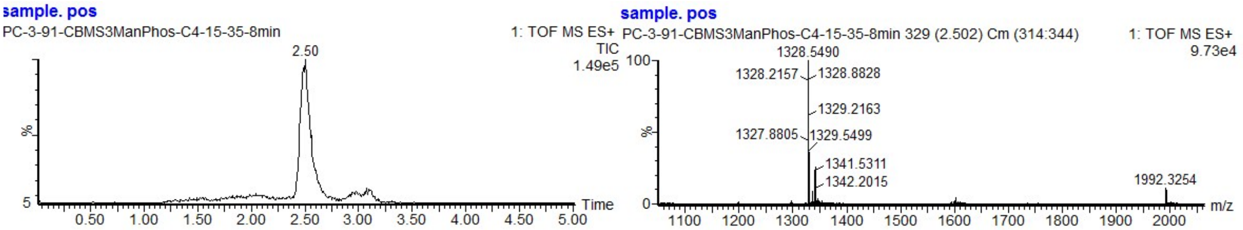

Figure S18. LC-MS trace and ESI-MS of purified CBM **20** (yield: 6%). LC-MS condition: 0.3 ml/min, 15%-35% MeCN in H_2O over 5 min. MS (ESI) Calcd for **20** $C_{165}H_{246}N_{43}O_{62}PS_4$ $[M+2H]^{2+}$ m/z = 1991.3097, $[M+3H]^{3+}$ m/z = 1327.8758.

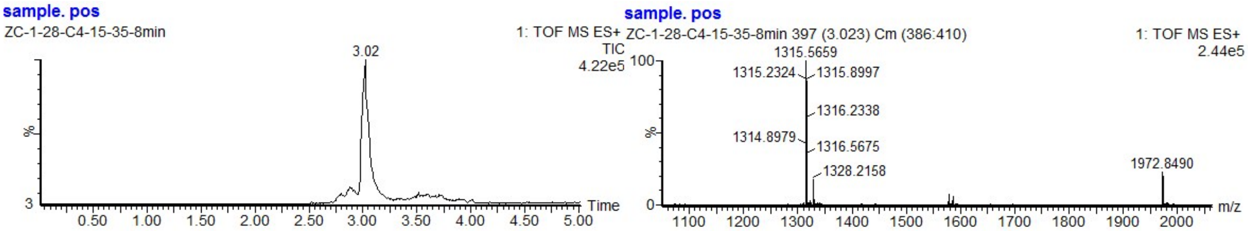

Figure S19. LC-MS trace and ESI-MS of purified CBM **21** (yield: 16%). LC-MS condition: 0.3 ml/min, 15%-35% MeCN in H_2O over 5 min. MS (ESI) Calcd for **21** $C_{167}H_{248}N_{44}O_{59}S_4$ $[M+2H]^{2+}$ m/z = 1971.8321, $[M+3H]^{3+}$ m/z = 1314.8880.

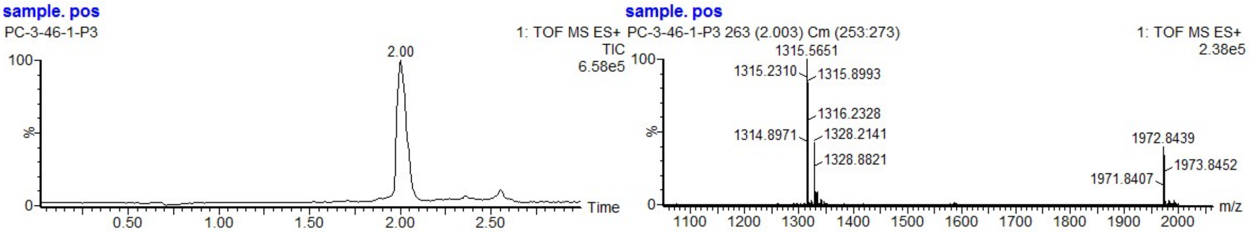

Figure S20. LC-MS trace and ESI-MS of purified CBM **22** (yield: 18%). LC-MS condition: 0.3 ml/min, 15%-35% MeCN in H_2O over 5 min. MS (ESI) Calcd for **22** $C_{167}H_{248}N_{44}O_{59}S_4$ $[M+2H]^{2+}$ m/z = 1971.8321, $[M+3H]^{3+}$ m/z = 1314.8880.

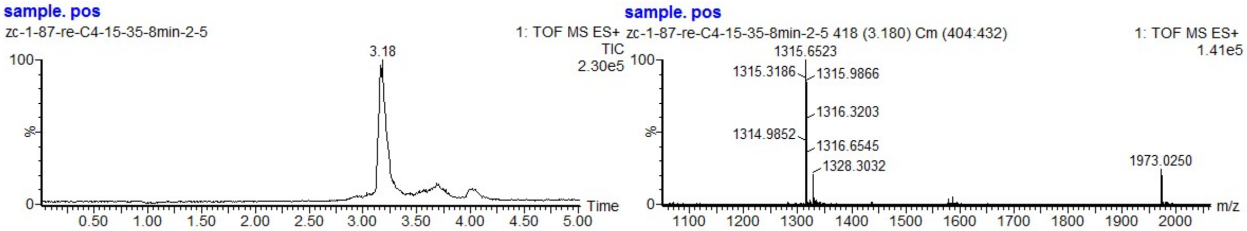

Figure S21. LC-MS trace and ESI-MS of purified CBM **23** (yield: 7%). LC-MS condition: 0.3 ml/min, 15%-35% MeCN in H_2O over 5 min. MS (ESI) Calcd for **23** $C_{167}H_{248}N_{44}O_{59}S_4$ $[M+2H]^{2+}$ m/z = 1971.8321, $[M+3H]^{3+}$ m/z = 1314.8880.

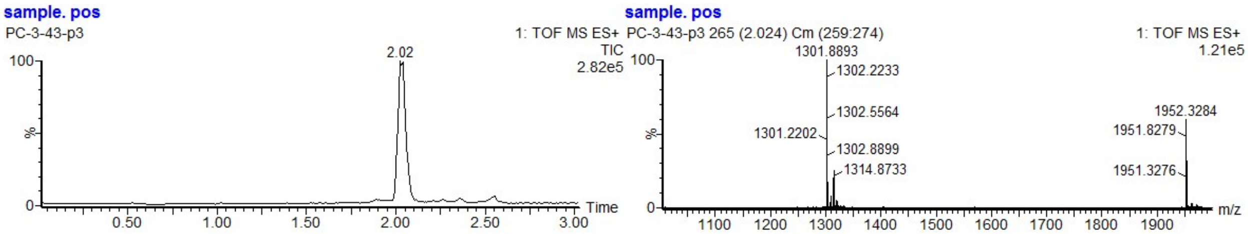

Figure S22. LC-MS trace and ESI-MS of purified CBM **24** (yield: 15%). LC-MS condition: 0.5 ml/min, 15%-35% MeCN in H_2O over 3 min. MS (ESI) Calcd for **24** $C_{165}H_{245}N_{43}O_{59}S_4$ $[M+2H]^{2+}$ m/z = 1951.3188, $[M+3H]^{3+}$ m/z = 1301.2125.

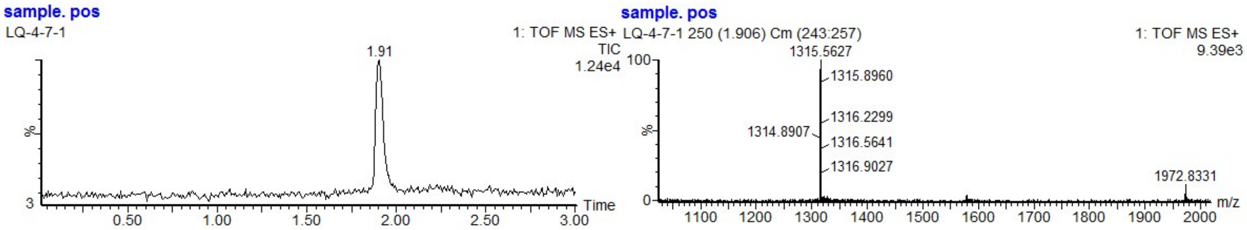

Figure S23. LC-MS trace and ESI-MS of purified CBM **25** (yield: 20%). LC-MS condition: 0.5 ml/min, 15%-35% MeCN in H_2O over 3 min. MS (ESI) Calcd for **25** $C_{167}H_{248}N_{44}O_{59}S_4$ $[M+2H]^{2+}$ m/z = 1971.8321, $[M+3H]^{3+}$ m/z = 1314.8880.

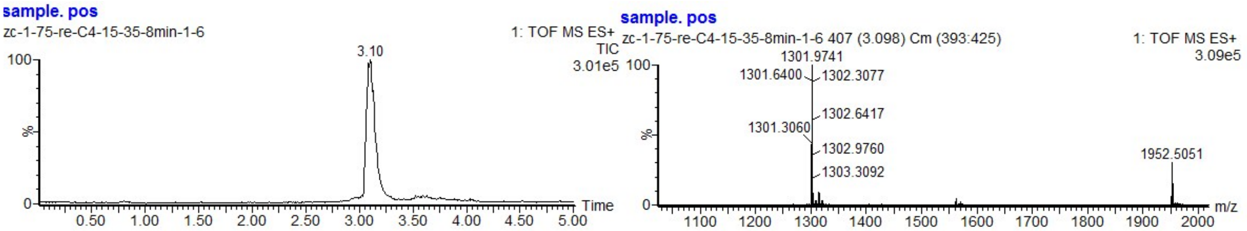

Figure S24. LC-MS trace and ESI-MS of purified CBM **26** (yield: 13%). LC-MS condition: 0.3 ml/min, 15%-35% MeCN in H_2O over 5 min. MS (ESI) Calcd for **26** $C_{165}H_{245}N_{43}O_{59}S_4$ $[M+2H]^{2+}$ m/z = 1951.3188, $[M+3H]^{3+}$ m/z = 1301.2125.

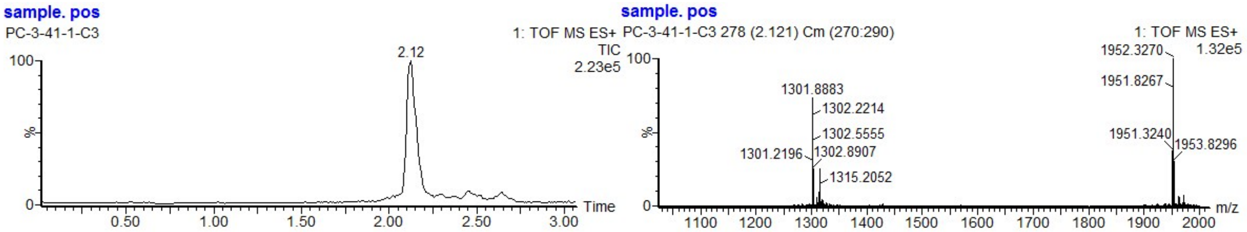

Figure S25. LC-MS trace and ESI-MS of purified CBM **27** (yield: 24%). LC-MS condition: 0.5 ml/min, 15%-35% MeCN in H_2O over 3 min. MS (ESI) Calcd for **27** $C_{165}H_{245}N_{43}O_{59}S_4$ $[M+2H]^{2+}$ m/z = 1951.3188, $[M+3H]^{3+}$ m/z = 1301.2125.

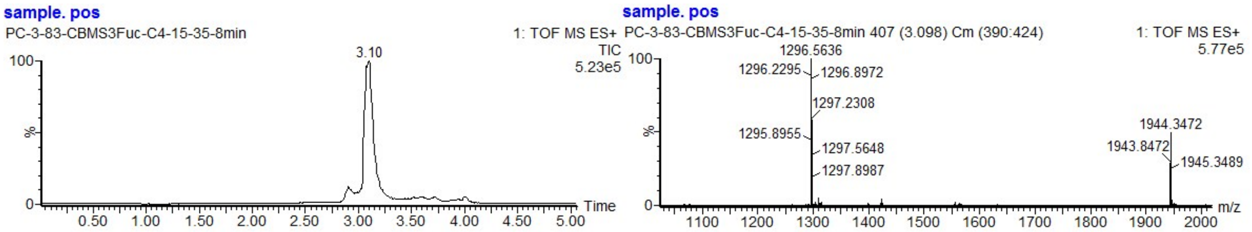

Figure S26. LC-MS trace and ESI-MS of purified CBM **28** (yield: 26%). LC-MS condition: 0.3 ml/min, 15%-35% MeCN in H_2O over 5 min. MS (ESI) Calcd for **28** $C_{165}H_{245}N_{43}O_{58}S_4$ $[M+2H]^{2+}$ m/z = 1943.3213, $[M+3H]^{3+}$ m/z = 1295.8809.

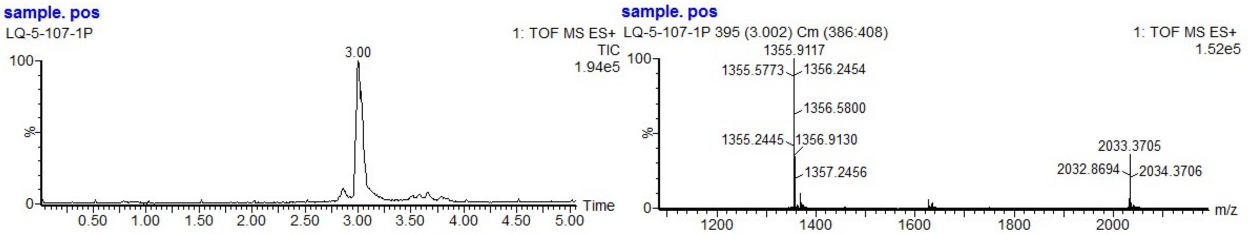

Figure S27. LC-MS trace and ESI-MS of purified CBM **29** (yield: 22%). LC-MS condition: 0.3 ml/min, 15%-35% MeCN in H_2O over 5 min. MS (ESI) Calcd for **29** $C_{171}H_{255}N_{43}O_{64}S_4$ $[M+2H]^{2+}$ m/z = 2032.3530, $[M+3H]^{3+}$ m/z = 1355.2379.

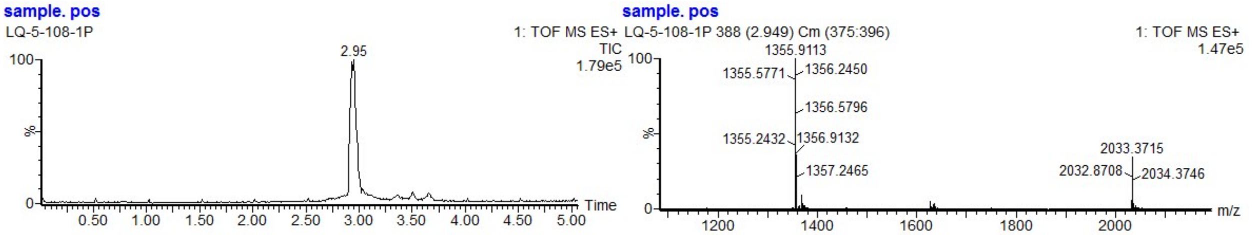

Figure S28. LC-MS trace and ESI-MS of purified CBM **30** (yield: 19%). LC-MS condition: 0.3 ml/min, 15%-35% MeCN in H_2O over 5 min. MS (ESI) Calcd for **30** $C_{171}H_{255}N_{43}O_{64}S_4$ $[M+2H]^{2+}$ m/z = 2032.3530, $[M+3H]^{3+}$ m/z = 1355.2379.

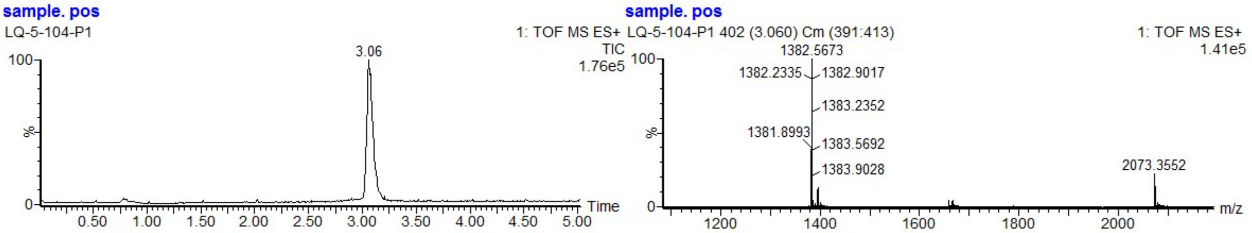

Figure S29. LC-MS trace and ESI-MS of purified CBM **31** (yield: 11%). LC-MS condition: 0.3 ml/min, 15%-35% MeCN in H_2O over 5 min. MS (ESI) Calcd for **31** $C_{171}H_{256}N_{43}O_{67}PS_4$ $[M+2H]^{2+}$ m/z = 2072.3284, $[M+3H]^{3+}$ m/z = 1381.8856.

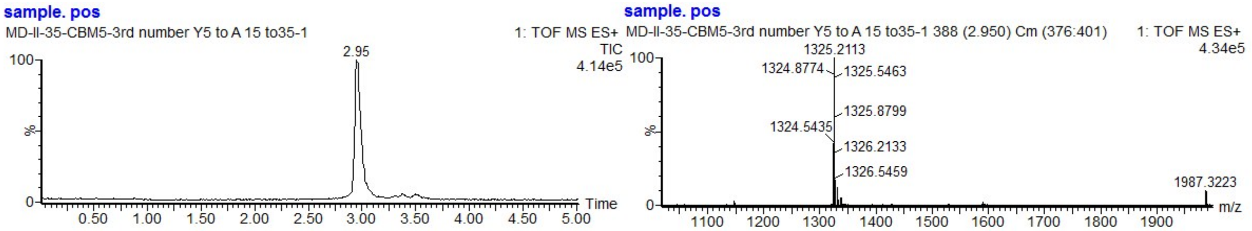

Figure S30. LC-MS trace and ESI-MS of purified CBM **32** (yield: 6%). LC-MS condition: 0.3 ml/min, 15%-35% MeCN in H_2O over 5 min. MS (ESI) Calcd for **32** $C_{165}H_{251}N_{43}O_{63}S_4$ $[M+2H]^{2+}$ m/z = 1986.3321, $[M+3H]^{3+}$ m/z = 1324.5547.

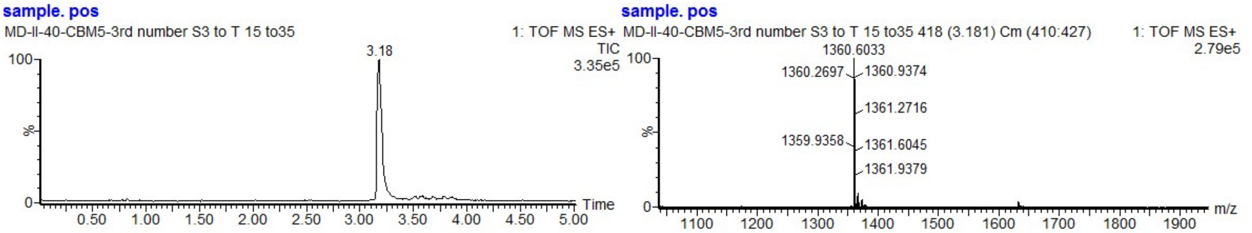

Figure S31. LC-MS trace and ESI-MS of purified CBM **33** (yield: 7%). LC-MS condition: 0.3 ml/min, 15%-35% MeCN in H_2O over 5 min. MS (ESI) Calcd for **33** $C_{172}H_{257}N_{43}O_{64}S_4$ $[M+2H]^{2+}$ m/z = 2039.3530, $[M+3H]^{3+}$ m/z = 1359.9020.

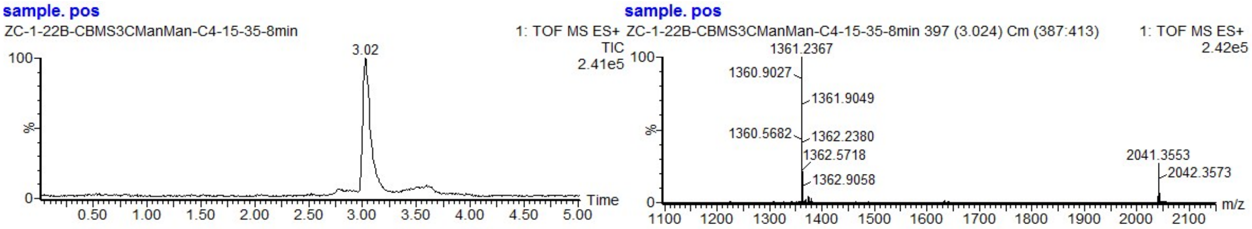

Figure S32. LC-MS trace and ESI-MS of purified CBM **34** (yield: 14%). LC-MS condition: 0.3 ml/min, 15%-35% MeCN in H_2O over 5 min. MS (ESI) Calcd for **34** $C_{171}H_{255}N_{43}O_{63}S_5$ $[M+2H]^{2+}$ m/z = 2040.3338, $[M+3H]^{3+}$ m/z = 1360.5558.

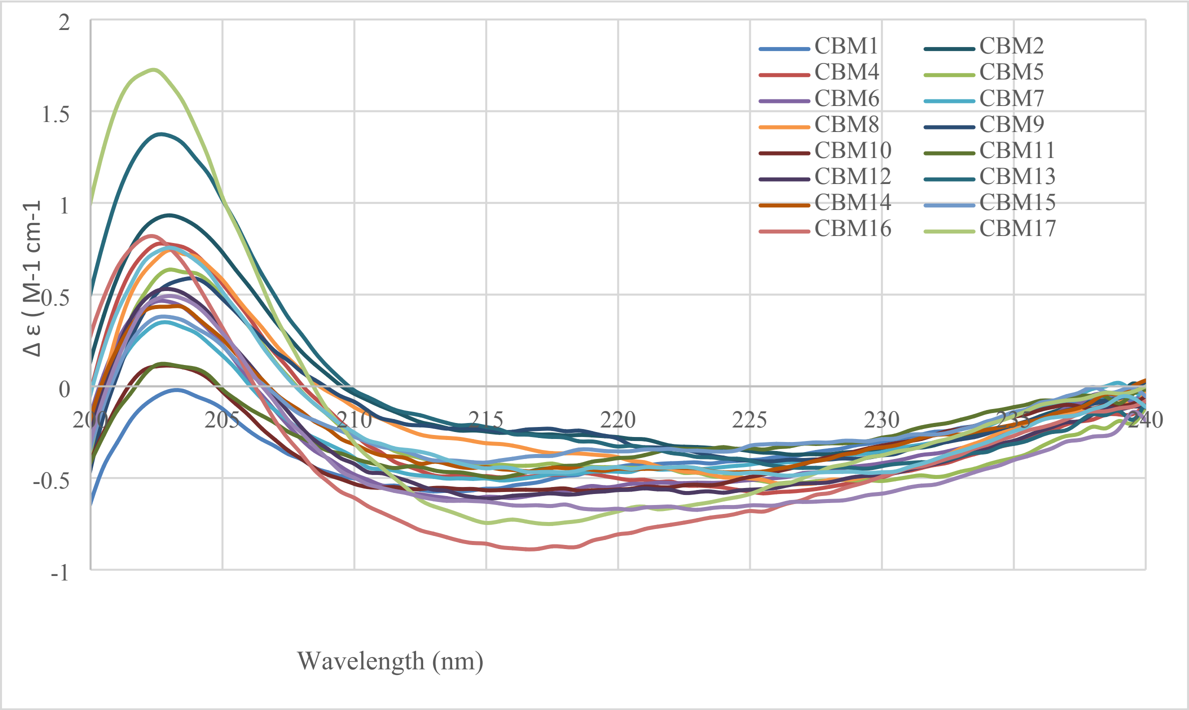

Figure S33. The CD spectra of CBM variants **1**, **2**, and **4-19**. The spectra were acquired in the same way as previously reported[18].

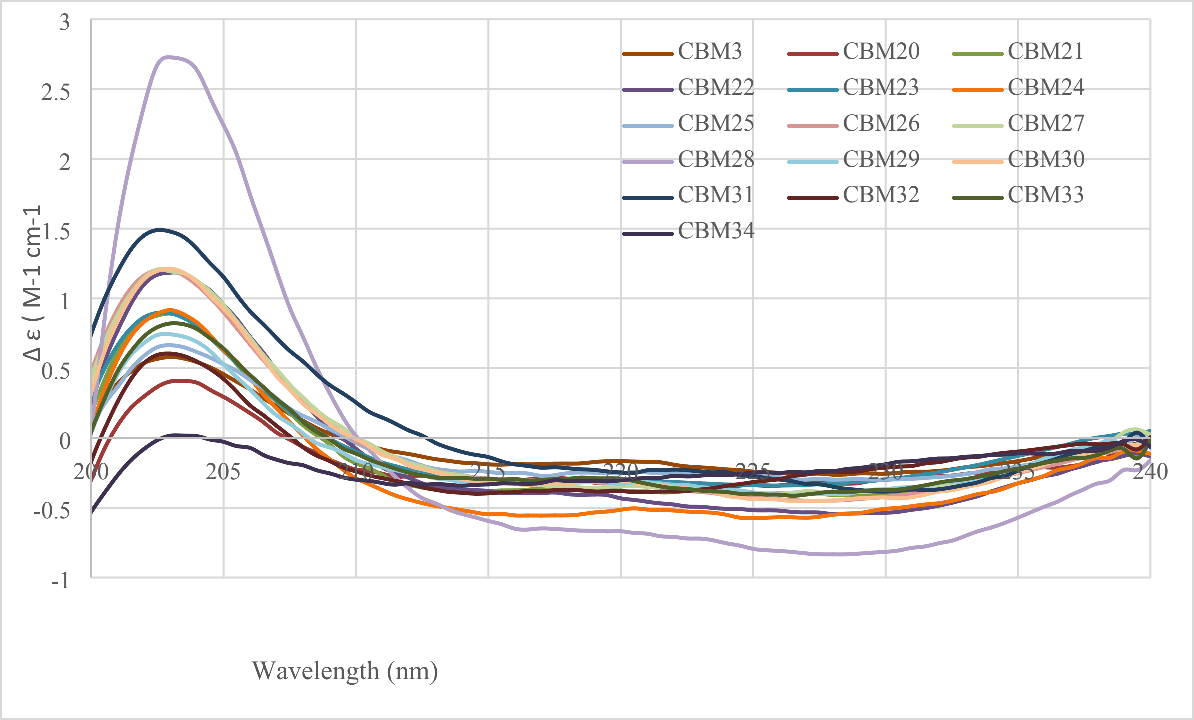

Figure S34. The CD spectra of CBM variants **3** and **20-34**. The spectra were acquired in the same way as previously reported[18].

Confirming disulfide linkages. The folding of the CBMs was confirmed as described in our previous report.[18] After HPLC purification, the UPLC-MS trace of the folded CBMs showed a single peak, which indicated the homogeneity of the product. The observed mass loss of 4 Da is consistent with the formation of two disulfide bridges. The far-ultraviolet CD spectra of the CBMs were very similar to previously obtained spectrum of the unglycosylated CBM, which suggested that the synthetic peptide adopted the appropriate secondary structure upon folding. Moreover, the UPLC-MS analysis revealed that the thermolysin digestion of CBMs produces two fragments that contain two short peptide chains, VC/YSQCL and YGQCGG/ASGTTCQV (or AGQCGG/ASGTTCQV for CBM **11**). These short peptide chains are connected by disulfide linkages, which clearly confirmed the correct disulfide connectivity (Fig. S35).

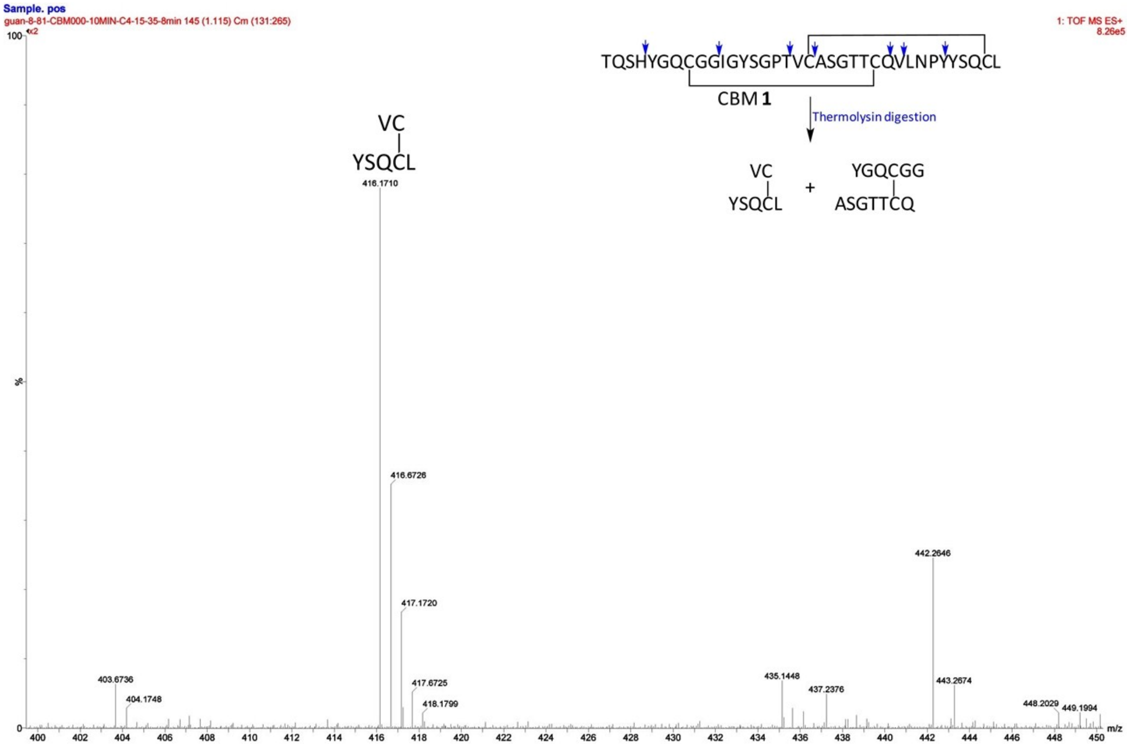

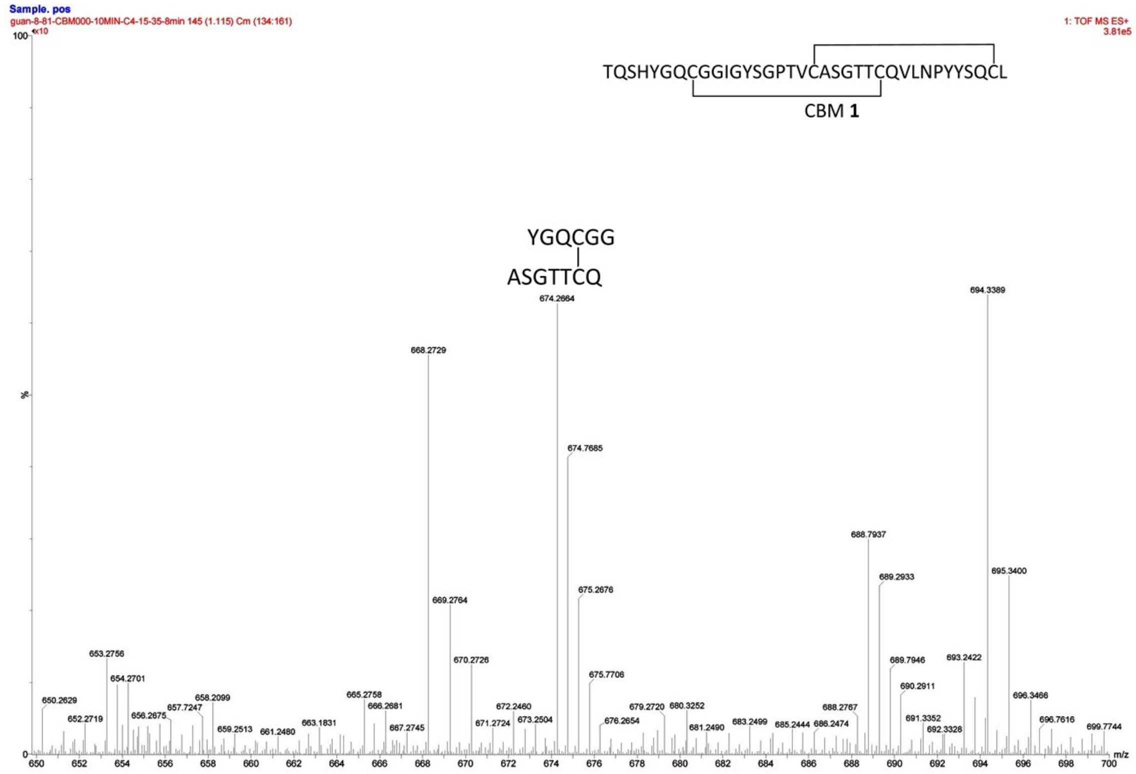
Sample. pos
guan-8-81-CBM000-10MIN-C4-15-35-8min 145 (1.115) Cm (134:161)
1: TOF MS ES+
3.81e5
TQSHYGQCGGIGYSGPTVCASGTTCQVLNPYYSQCL
CBM 1
YGQCGG
ASGTTCQ
674.2664
694.3389
668.2729
674.7685
688.7937
689.2933
695.3400
669.2764
675.2676
670.2726
653.2756
689.7946
693.2422
654.2701
675.7706
m/z

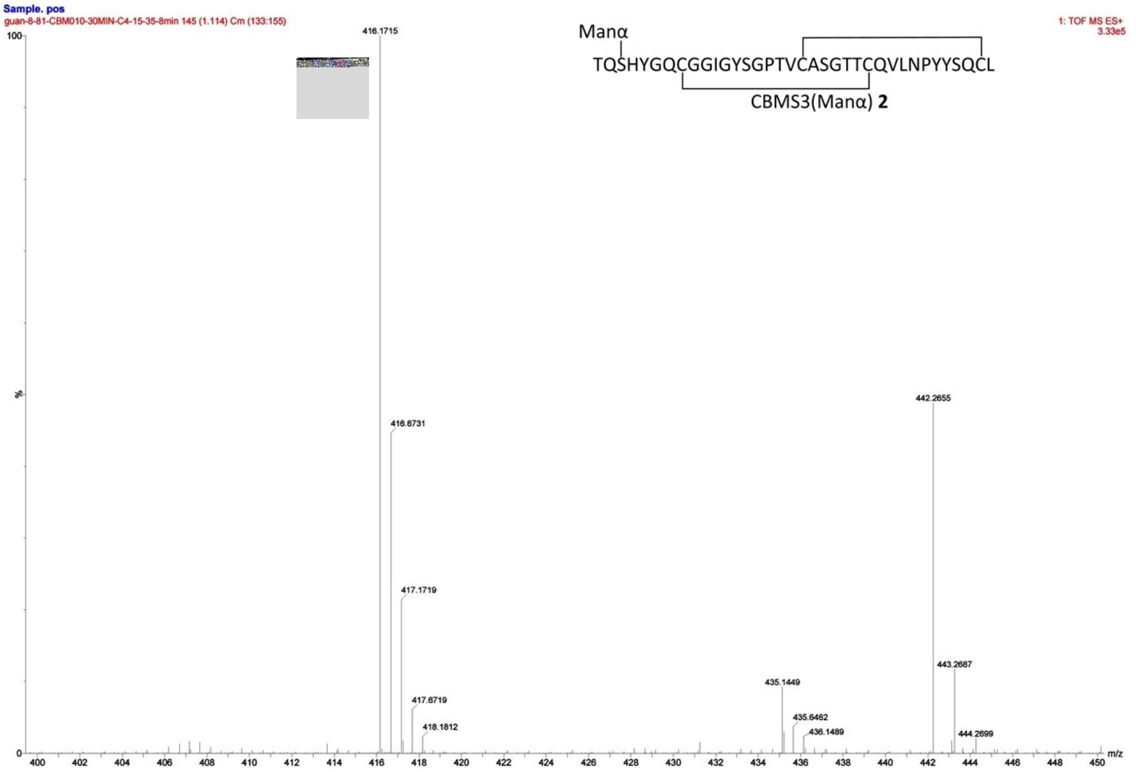
Sample. pos
guan-8-81-CBM010-30MIN-C4-15-35-8min 145 (1.114) Cm (133:155)
1: TOF MS ES+
3.33e5
Manα
TQSHYGQCGGIGYSGPTVCASGTTCQVLNPYYSQCL
CBMS3(Manα) 2
416.1715
416.6731
442.2655
417.1719
443.2687
435.1449
417.6719
435.6482
418.1812
436.1489
444.2699
m/z

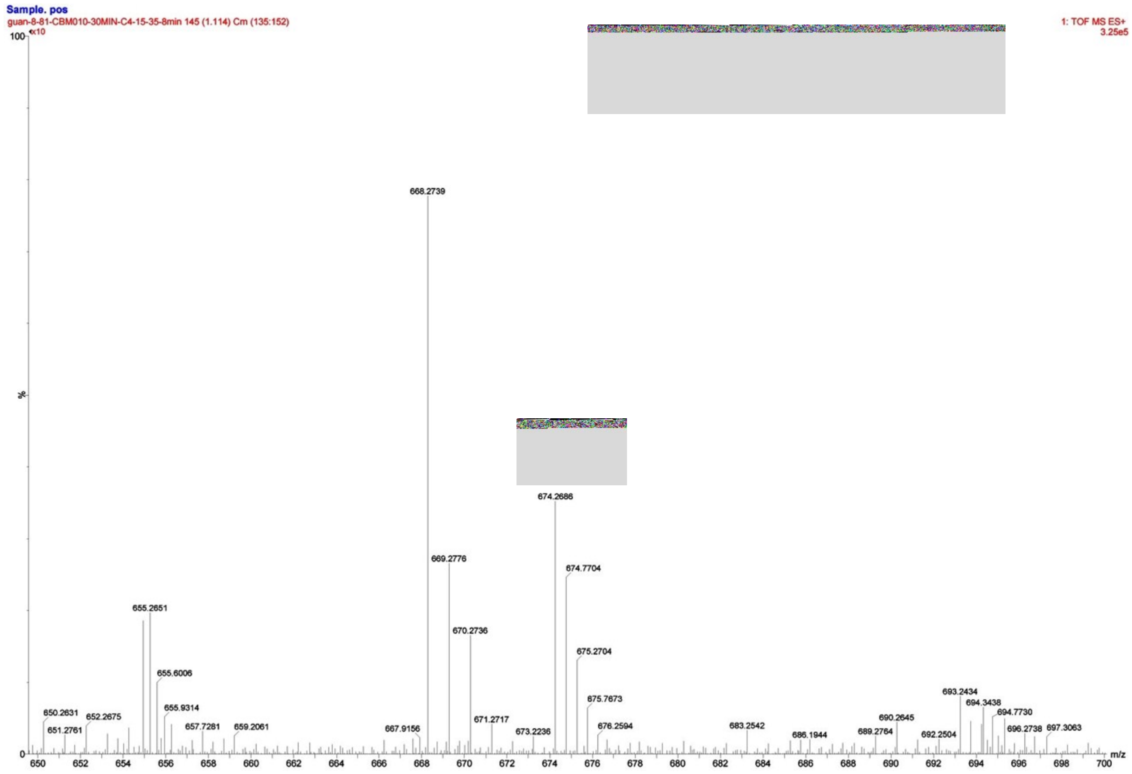
Sample. pos
guan-8-81-CBM010-30MIN-C4-15-35-8min 145 (1.114) Cm (135:152)
1: TOF MS ES+
3.25e5
x10
668.2739
674.2686
669.2776
674.7704
655.2651
670.2736
675.2704
655.6006
675.7673
655.9314
650.2631
651.2761
652.2675
657.7281
659.2061
667.9156
671.2717
673.2236
676.2594
683.2542
686.1944
689.2764
690.2645
692.2504
693.2434
694.3438
694.7730
696.2738
697.3063
m/z

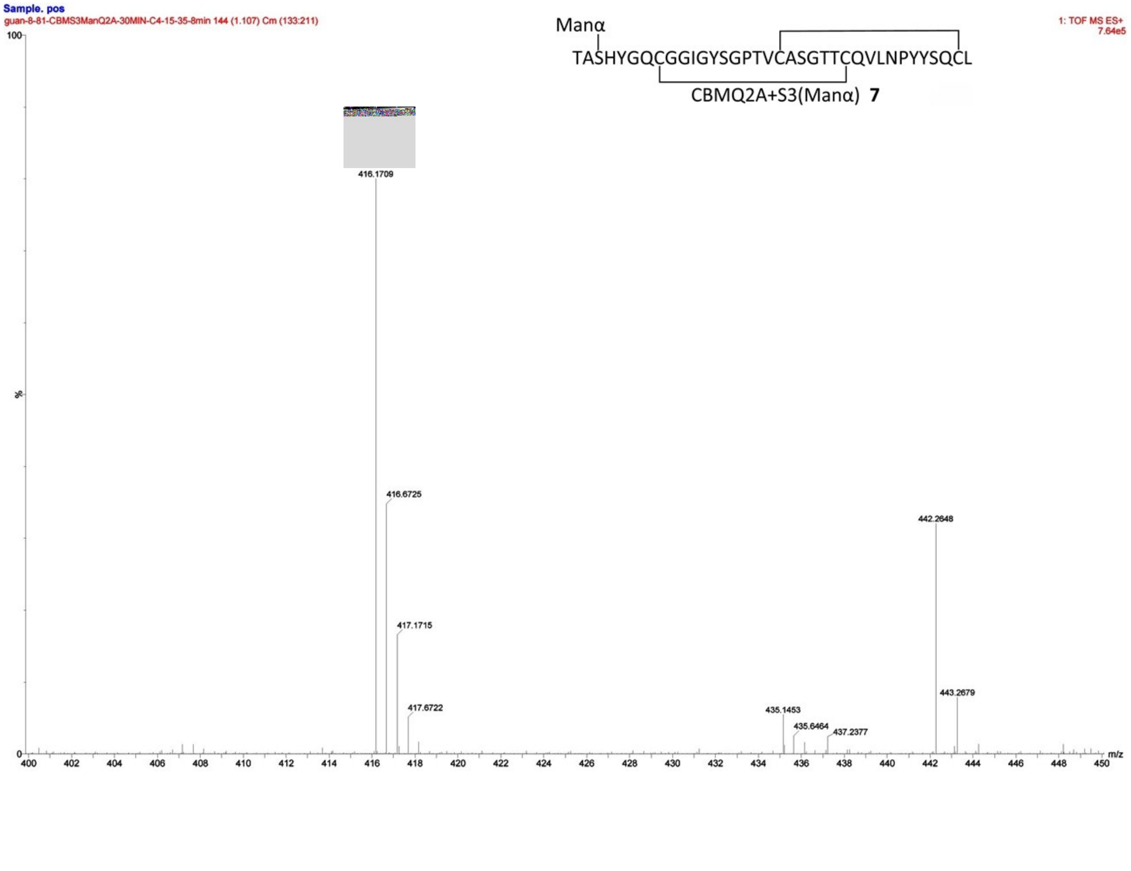
Sample. pos
guan-8-81-CBMS3ManQ2A-30MIN-C4-15-35-8min 144 (1.107) Cm (133:211)
1: TOF MS ES+
7.64e5
Manα
TASHYGQCGGIGYSGPTVCASGTTCQVLNPYYSQCL
CBMQ2A+S3(Manα) 7
416.1709
416.6725
442.2648
417.1715
443.2679
417.6722
435.1453
435.6464
437.2377
m/z

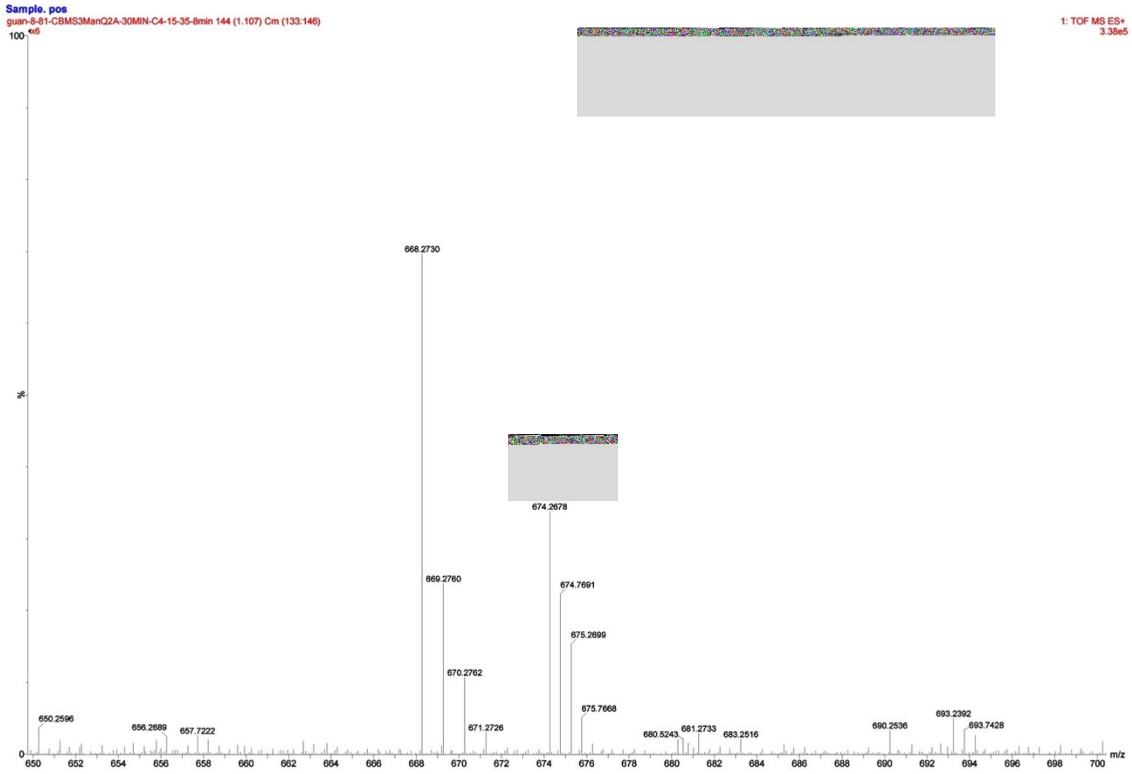
Sample. pos
guan-8-81-CBMS3ManQ2A-30MIN-C4-15-35-8min 144 (1.107) Cm (133:146)
1: TOF MS ES+
3.38e5
668.2730
669.2760
670.2762
671.2726
674.2678
674.7691
675.2699
675.7668
650.2596
656.2689
657.7222
680.5243
681.2733
683.2516
690.2536
693.2392
693.7428
m/z

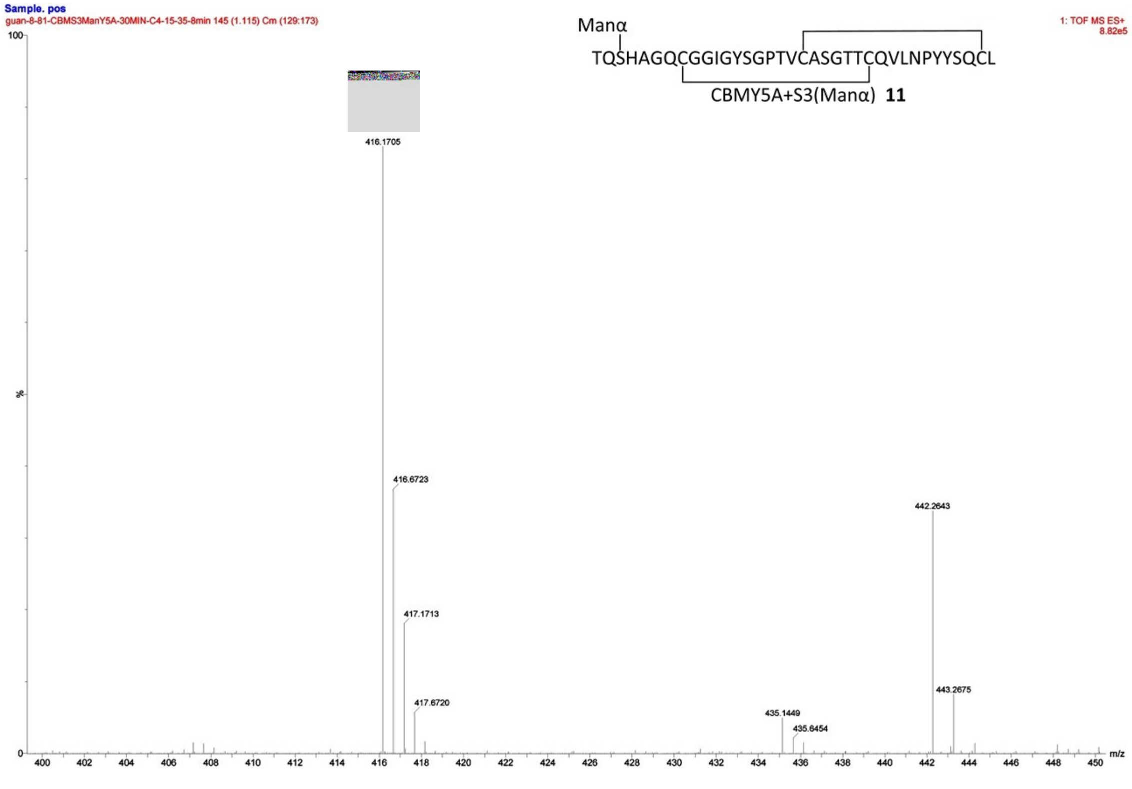
Sample. pos
guan-8-81-CBMS3ManY5A-30MIN-C4-15-35-8min 145 (1.115) Cm (129:173)
1: TOF MS ES+
8.82e5
Manα
TQSHAGQCGGIGYSGPTVCASGTTCQVLNPYYSQCL
CBMY5A+S3(Manα) 11
416.1705
416.6723
417.1713
417.6720
435.1449
435.6454
442.2643
443.2675
m/z

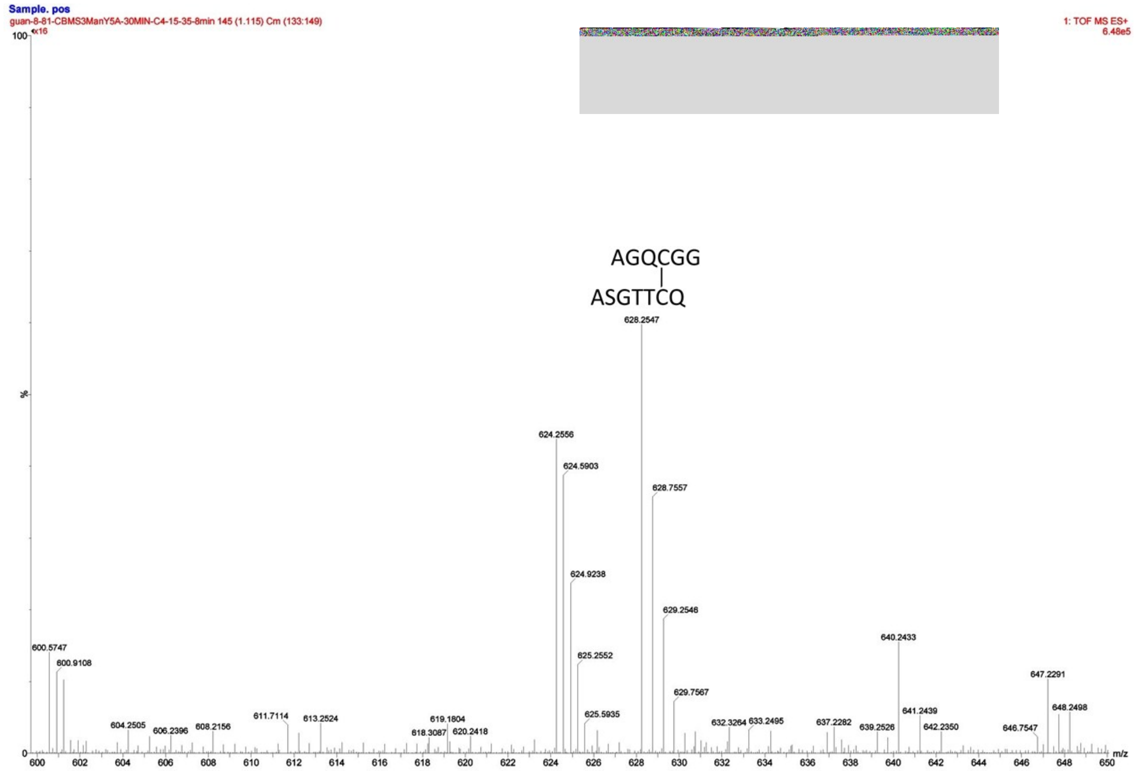
Sample. pos
guan-8-81-CBMS3ManY5A-30MIN-C4-15-35-8min 145 (1.115) Cm (133:149)
1: TOF MS ES+
6.48e5
x16
AGQCGG
ASGTTCQ
628.2547
624.2556
624.5903
628.7557
624.9238
629.2546
640.2433
600.5747
600.9108
625.2552
647.2291
629.7567
648.2498
611.7114
613.2524
619.1804
625.5935
637.2282
641.2439
604.2505
606.2396
608.2156
618.3087
620.2418
632.3264
633.2495
639.2526
642.2350
646.7547
%
m/z

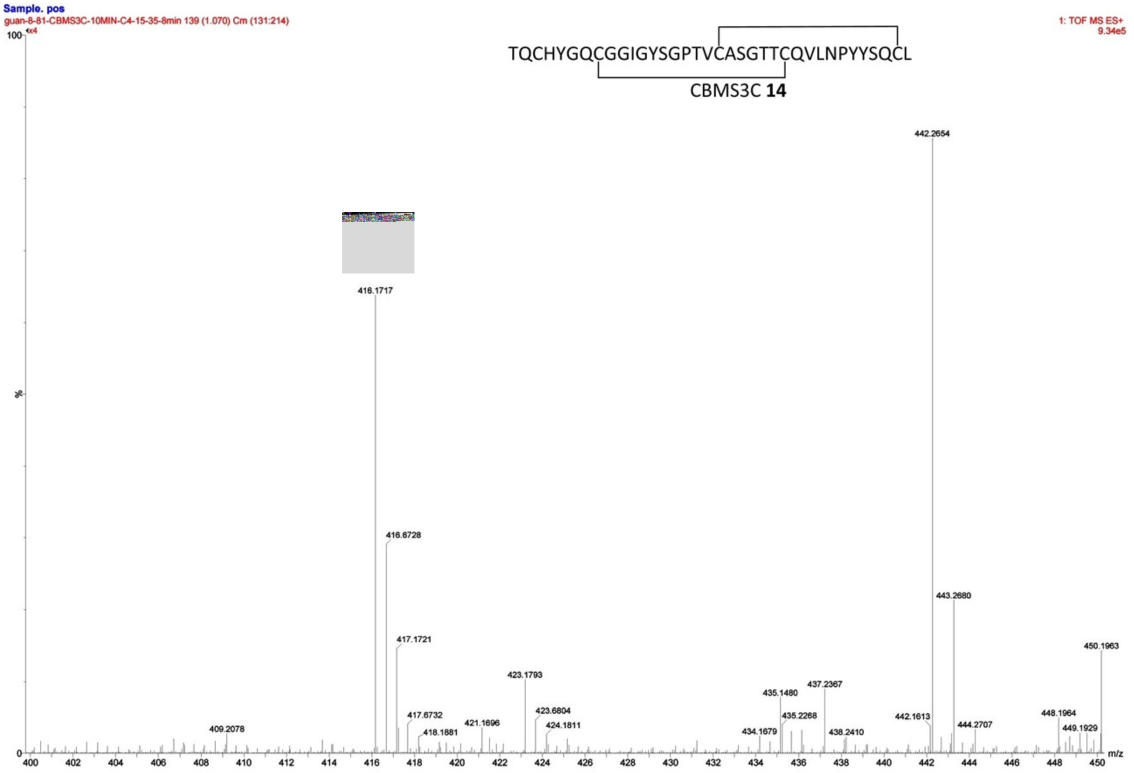
Sample. pos
guan-8-81-CBMS3C-10MIN-C4-15-35-8min 139 (1.070) Cm (131:214)
1: TOF MS ES+
9.34e5
x4
TQCHYGQCGGIGYSGPTVCASGTTCQVLNPYYSQCL
CBMS3C 14
442.2654
416.1717
416.6728
443.2680
417.1721
450.1963
423.1793
437.2367
435.1480
423.6804
417.6732
424.1811
409.2078
418.1881
421.1696
434.1679
435.2268
438.2410
442.1613
444.2707
448.1964
449.1929
%
m/z

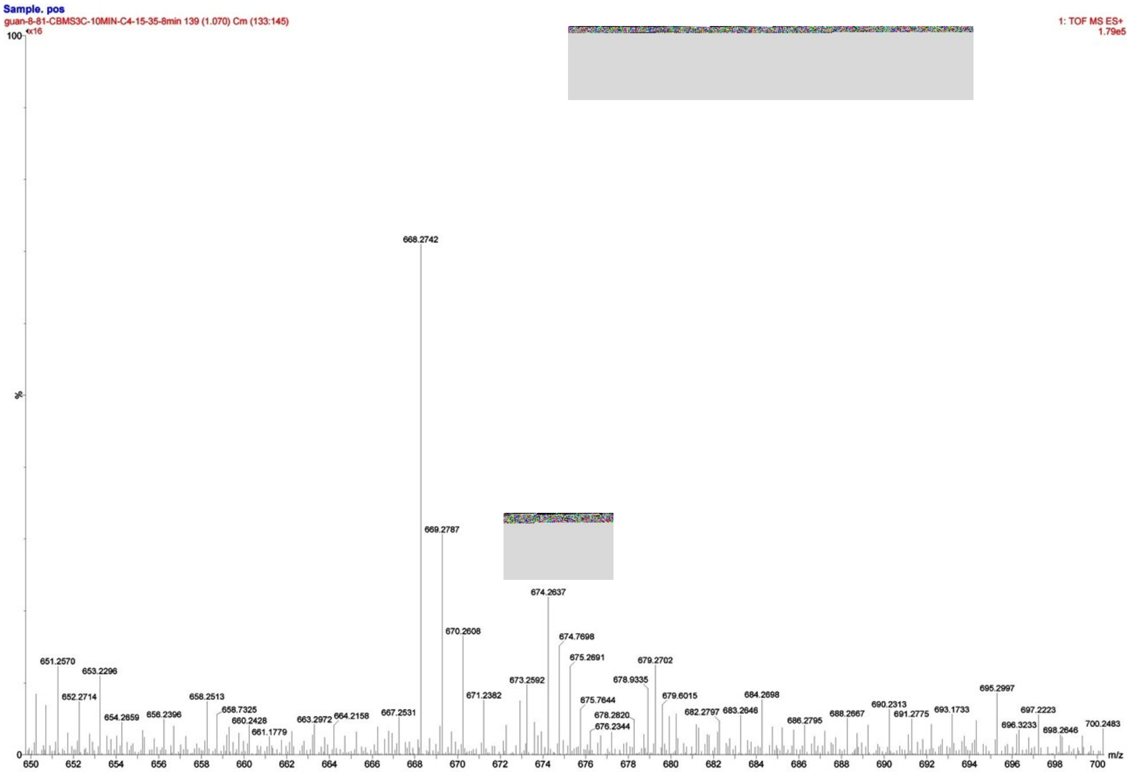
Sample. pos
guan-8-81-CBMS3C-10MIN-C4-15-35-8min 139 (1.070) Cm (133:145)
1: TOF MS ES+
1.79e5
x16
100
%
0
668.2742
669.2787
674.2637
670.2608
674.7698
675.2691
651.2570
653.2296
679.2702
678.9335
673.2592
695.2997
652.2714
658.2513
671.2382
679.6015
684.2698
690.2313
658.7325
675.7644
682.2797
683.2646
691.2775
693.1733
697.2223
654.2659
656.2396
660.2428
663.2972
664.2158
667.2531
678.2820
676.2344
686.2795
688.2667
696.3233
698.2646
700.2483
661.1779
m/z

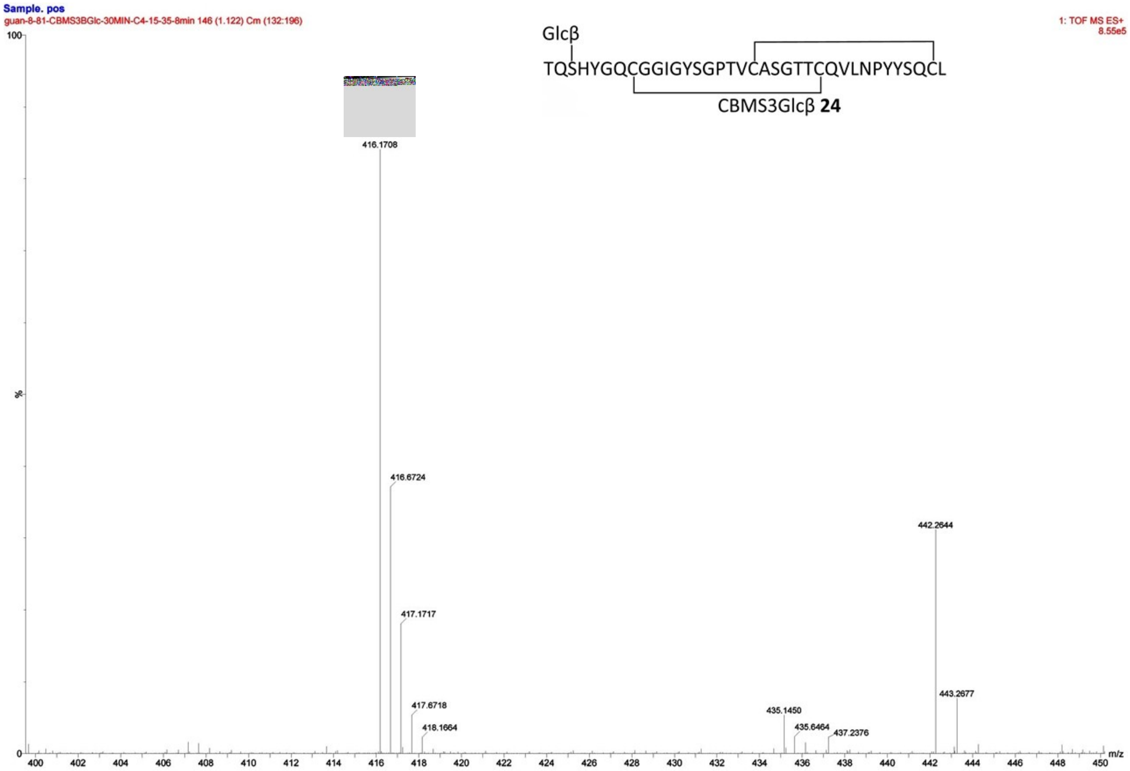
Sample. pos
guan-8-81-CBMS3BGlc-30MIN-C4-15-35-8min 146 (1.122) Cm (132:196)
1: TOF MS ES+
8.55e5
Glcβ
TQSHYGQCGGIGYSGPTVCASGTTCQVLNPYYSQCL
CBMS3Glcβ **24**
100
%
0
416.1708
416.6724
442.2644
417.1717
443.2677
417.6718
435.1450
418.1664
435.6464
437.2376
m/z

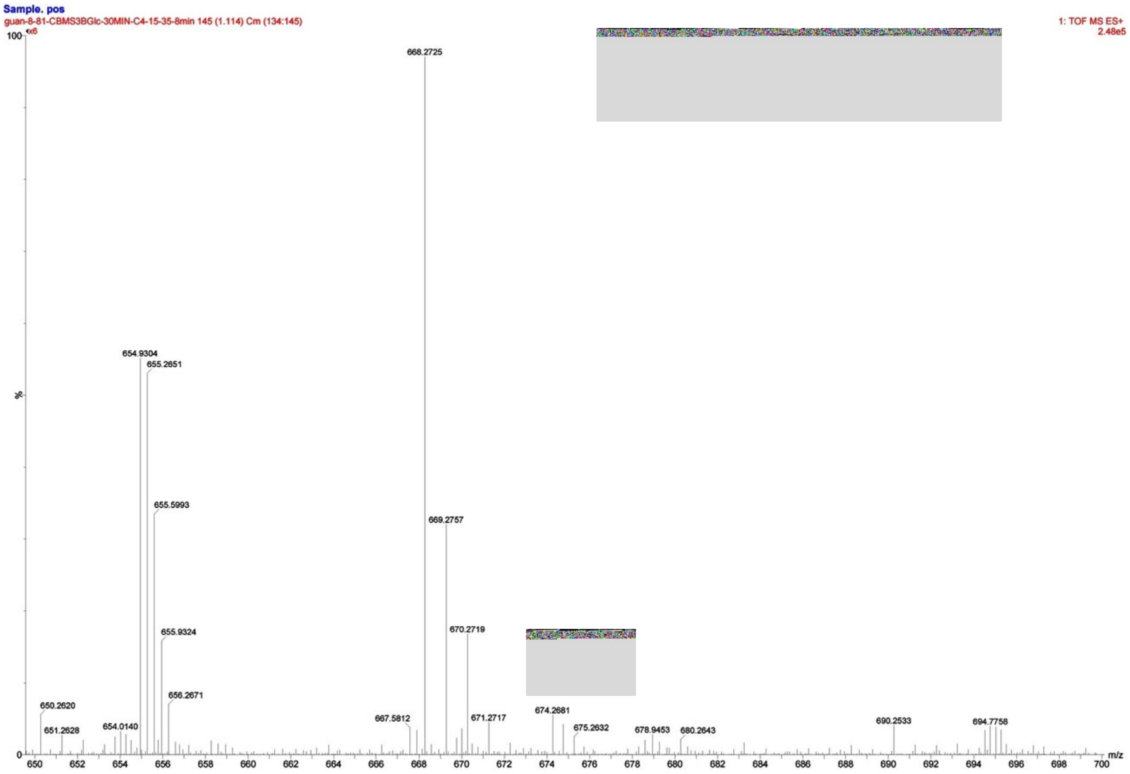

Figure S35. Confirming disulfide linkages of representative CBM variants by thermolysin digestion. MS (ESI) Calcd for VC/YSQCL $C_{34}H_{54}N_8O_{12}S_2$ $[M+2H]^{2+}$ m/z = 416.1730; MS (ESI) Calcd for YGQCGG/ASGTTCQV $C_{52}H_{82}N_{16}O_{22}S_2$ $[M+2H]^{2+}$ m/z = 674.2693; MS (ESI) Calcd for AGQCGG/ASGTTCQV $C_{46}H_{78}N_{16}O_{21}S_2$ $[M+2H]^{2+}$ m/z = 628.2562.

Table S1. Summary of CBM proteolytic stability, thermostability, and adsorption to crystalline cellulose that were determined in accordance with our procedures as described previously[18].

CBM Variant	Half-Life to Thermolysin Degradation (h)	T_m (°C)	K_{ads} (μM^{-1})	B_{max} (µmol/g)
**1**	0.23 ± 0.02	62.2 ± 0.6	0.0894 ± 0.0007*	24 ± 5*
**2**	1.09 ± 0.01	70.1 ± 0.9	0.4 ± 0.2	6 ± 1.3
**3**	2.13 ± 0.06	73.2 ± 0.7	0.21 ± 0.05	3.6 ± 0.8
**4**	0.16 ± 0.02	64.2 ± 0.6	0.20 ± 0.11	8.1 ± 1.1
**5**	0.74 ± 0.12	69.2 ± 0.9	0.20 ± 0.02	13 ± 2
**6**	0.16 ± 0.04	63.4 ± 0.7	0.40 ± 0.2	16 ± 3
**7**	0.27 ± 0.05	63.5± 0.7	0.32 ± 0.18	11 ± 2
**8**	0.22 ± 0.06	61.2 ± 0.3	0.53 ± 0.12	5.7 ± 0.7
**9**	0.65 ± 0.05	65.6 ± 0.3	0.73 ± 0.18	8.7 ± 1.0
**10**	0.08 ± 0.02	61.1 ± 1.0	~0**	~0**
**11**	0.18 ± 0.03	61.3 ± 0.5	~0**	~0**
**12**	0.36 ± 0.02	61.8 ± 1.6	0.18 ± 0.04	14 ± 4
**13**	1.14 ± 0.18	69.3 ± 0.4	0.30 ± 0.19	9.2 ± 1.6
**14**	0.18 ± 0.04	53.2 ± 0.7	0.32 ± 0.15	24 ± 4
**15**	0.20 ± 0.03	61.3 ± 0.9	0.22 ± 0.08	12 ± 1
**16**	0.19 ± 0.04	62.6 ± 1.6	~0**	~0**
**17**	0.17 ± 0.02	63.6 ± 1.3	~0**	~0**
**18**	0.30 ± 0.01	62.7 ± 1.7	0.17 ± 0.04	16 ± 2
**19**	0.34 ± 0.07	63.6 ± 0.7	0.20 ± 0.11	10 ± 1
**20**	0.57 ± 0.06	65.9 ± 0.3	0.28 ± 0.07	16 ± 1
**21**	0.69 ± 0.09	68.4 ± 1.4	0.19 ± 0.04	6.8 ± 1.3
**22**	0.40 ± 0.01	67.5 ± 0.4	0.40 ± 0.27	10 ± 3
**23**	0.32 ± 0.01	63.0 ± 0.3	0.12 ± 0.01	13 ± 1
**24**	0.28 ± 0.03	64.2 ± 0.1	0.14 ± 0.08	7.5 ± 2.4
**25**	0.38 ± 0.05	67.3 ± 1.7	0.52 ± 0.13	18 ± 2
**26**	0.50 ± 0.05	68.2 ± 0.9	0.17 ± 0.08	14 ± 4
**27**	0.29 ± 0.02	63.8 ± 0.5	0.18 ± 0.01	10 ± 1
**28**	0.62 ± 0.11	66.5 ± 0.2	0.25 ± 0.04	11 ± 1
**29**	0.86 ± 0.03	68.5 ± 1.2	0.25 ± 0.04	16 ± 2
**30**	0.78 ± 0.11	68.4 ± 0.7	0.51 ± 0.06	10 ± 1
**31**	0.43 ± 0.05	67.4 ± 0.8	0.25 ± 0.07	11 ± 1
**32**	0.10 ± 0.02	61.5 ± 1.1	~0**	~0**
**33**	1.56 ± 0.40	71.9 ± 0.4	0.51 ± 0.04	4.7 ± 0.2
**34**	0.18 ± 0.02	60.2 ± 1.2	0.15 ± 0.05	18 ± 3

The results are presented as mean of three trials ± SD. *Denotes an averaged value of four trials ± SD. **Weak affinity to cellulose noted, no K_{ads} and B_{max} value could be obtained.

Table S2. The change in half-life and melting temperature from the corresponding unglycosylated CBMs.

Calculation	Change in Half-Life to Thermolysin Degradation (h)	Change in melting temperature (°C)	Change in binding affinity (μM^{-1})
**2-1**	0.86	7.9	0.31
**3-1**	1.90	11.0	0.12
**5-4**	0.58	5.0	0
**7-6**	0.11	0.1	-0.08
**9-8**	0.43	4.4	0.2
**11-10**	0.10	0.2	0
**13-12**	0.78	7.5	0.12
**15-14**	0.02***	8.1***	-0.10***
**17-16**	-0.02	1.0	0
**19-18**	0.04	0.9	0.03
**20-1**	0.34	3.7	0.19
**21-1**	0.46	6.2	0.10
**22-1**	0.17	5.3	0.31
**23-1**	0.09	0.8	0.03
**24-1**	0.05	2.0	0.05
**25-1**	0.15	5.1	0.43
**26-1**	0.27	6.0	0.08
**27-1**	0.06	1.6	0.09
**28-1**	0.39	4.3	0.16
**29-1**	0.63	6.3	0.16
**30-1**	0.55	6.2	0.42
**31-1**	0.20	5.2	0.16
**32-10**	0.02	0.4	0
**33-12**	1.2	10.1	0.33
**34-14**	0***	7.0***	-0.17***

*** Not used in the correlation plot.

REFERENCES:

1. Chen, L. & Tan, Z. A convenient and efficient synthetic approach to mono-, di-, and tri-O-mannosylated Fmoc amino acids. *Tetrahedron Lett.* **54**, 2190-2193, (2013).
2. Cui, H. K. *et al.* Diaminodiacid-based solid-phase synthesis of peptide disulfide bond mimics. *Angew. Chem. Int. Ed. Engl.* **52**, 9558-9562, (2013).
3. Yu, H. & Chen, X. Aldolase-catalyzed synthesis of *β*-D-gal*p*-(1→9)-D-KDN: a novel acceptor for sialyltransferases. *Org. Lett.* **8**, 2393-2396, (2006).
4. Liu, Y., Marshall, J., Li, Q., Edwards, N. & Chen, G. Synthesis of novel bivalent mimetic ligands for mannose-6-phosphate receptors. *Bioorg. Med. Chem. Lett.* **23**, 2328-2331, (2013).
5. Zervas, L. & Dilaris, I. Dealkylation and debenzylation of triesters of phosphoric acid.

Phosphorylation of hydroxy and amino compounds. *J. Am. Chem. Soc.* **77**, 5354-5357, (1955).
6. Plattner, C., Hofener, M. & Sewald, N. One-pot azidochlorination of glycals. *Org. Lett.* **13**, 545-547, (2011).
7. Rothman, D. M., Vazquez, M. E., Vogel, E. M. & Imperiali, B. Caged phospho-amino acid building blocks for solid-phase peptide synthesis. *J. Org. Chem.* **68**, 6795-6798, (2003).
8. Rosen, T., Lico, I. M. & Chu, D. T. W. A convenient and highly chemoselective method for the reductive acetylation of azides. *J.Org. Chem.* **53**, 1580-1582, (1988).
9. Salvador, L. A., Elofsson, M. & Kihlberg, J. Preparation of building-blocks for glycopeptide synthesis by glycosylation of Fmoc amino-acids having unprotected carboxyl groups. *Tetrahedron* **51**, 5643-5656, (1995).
10. Ohlsson, J. & Magnusson, G. Galabiosyl donors; efficient synthesis from 1,2,3,4,6-penta-O-acetyl-β-d-galactopyranose. *Carbohydrate Research* **329**, 49-55, (2000).
11. Nishi, Y. & Tanimoto, T. Preparation and characterization of branched beta-cyclodextrins having alpha-L-fucopyranose and a study of their functions. *Biosci. Biotechnol. Biochem.* **73**, 562-569, (2009).
12. Nicolaou, K. C., Hummel, C. W. & Iwabuchi, Y. Total Synthesis of Sialyl Dimeric Lex. *J. Am. Chem. Soc.* **114**, 3126-3128, (1992).
13. Hiruma-Shimizu, K. *et al.* Chemical synthesis, folding, and structural insights into O-fucosylated epidermal growth factor-like repeat 12 of mouse Notch-1 receptor. *J. Am. Chem. Soc.* **132**, 14857-14865, (2010).
14. Peters, S., Lowary, T. L., Hindsgaul, O., Meldal, M. & Bock, K. Solid-Phase Synthesis of a fucosylated glycopeptide of human factor-IX with a fucose-α-(1→O)-serine linkage. *J. Chem. Soc., Perkin Trans. 1*, 3017-3022, (1995).
15. Wu, L. & Sampson, N. S. Fucose, mannose, and *β*-N-acetylglucosamine glycopolymers initiate the mouse sperm acrosome reaction through convergent signaling pathways. *ACS Chem. Biol.* **9**, 468-475, (2014).
16. Lin, Y. A., Chalker, J. M. & Davis, B. G. Olefin cross-metathesis on proteins: investigation of allylic chalcogen effects and guiding principles in metathesis partner selection. *J. Am. Chem. Soc.* **132**, 16805-16811, (2010).
17. Zeng, J., Vedachalam, S., Xiang, S. & Liu, X. W. Direct C-glycosylation of organotrifluoroborates with glycosyl fluorides and its application to the total synthesis of (+)-varitriol. *Org. Lett.* **13**, 42-45, (2011).
18. Chen, L. *et al.* Specificity of O-glycosylation in enhancing the stability and cellulose binding affinity of Family 1 carbohydrate-binding modules. *Proc. Natl. Acad. Sci. U S A* **111**, 7612-7617, (2014).
